# Mitochondrial Cristae Morphology Reflecting Metabolism, Superoxide Formation, Redox Homeostasis, and Pathology

**DOI:** 10.1089/ars.2022.0173

**Published:** 2023-10-16

**Authors:** Petr Ježek, Martin Jabůrek, Blanka Holendová, Hana Engstová, Andrea Dlasková

**Affiliations:** Department No. 75, Institute of Physiology, Academy of Sciences of the Czech Republic, Prague, Czech Republic.

**Keywords:** mitochondrial cristae, mitochondrial superoxide formation, ATP-synthase dimeric rows, MICOS, OPA1, respiratory chain supercomplexes

## Abstract

**Significance:**

Mitochondrial (mt) reticulum network in the cell possesses amazing ultramorphology of parallel lamellar cristae, formed by the invaginated inner mitochondrial membrane. Its non-invaginated part, the inner boundary membrane (IBM) forms a cylindrical sandwich with the outer mitochondrial membrane (OMM). Crista membranes (CMs) meet IBM at crista junctions (CJs) of mt cristae organizing system (MICOS) complexes connected to OMM sorting and assembly machinery (SAM). Cristae dimensions, shape, and CJs have characteristic patterns for different metabolic regimes, physiological and pathological situations.

**Recent Advances:**

Cristae-shaping proteins were characterized, namely rows of ATP-synthase dimers forming the crista lamella edges, MICOS subunits, optic atrophy 1 (OPA1) isoforms and mitochondrial genome maintenance 1 (MGM1) filaments, prohibitins, and others. Detailed cristae ultramorphology changes were imaged by focused-ion beam/scanning electron microscopy. Dynamics of crista lamellae and mobile CJs were demonstrated by nanoscopy in living cells. With tBID-induced apoptosis a single entirely fused cristae reticulum was observed in a mitochondrial spheroid.

**Critical Issues:**

The mobility and composition of MICOS, OPA1, and ATP-synthase dimeric rows regulated by post-translational modifications might be exclusively responsible for cristae morphology changes, but ion fluxes across CM and resulting osmotic forces might be also involved. Inevitably, cristae ultramorphology should reflect also mitochondrial redox homeostasis, but details are unknown. Disordered cristae typically reflect higher superoxide formation.

**Future Directions:**

To link redox homeostasis to cristae ultramorphology and define markers, recent progress will help in uncovering mechanisms involved in proton-coupled electron transfer *via* the respiratory chain and in regulation of cristae architecture, leading to structural determination of superoxide formation sites and cristae ultramorphology changes in diseases. *Antioxid. Redox Signal.* 39, 635–683.

## I. Introduction

### A. Milestones of mitochondrial research

Mitochondria were named around 170 years ago as organelles that were morphologically described as threads (Greek “mitos”) and grains (Greek “chondros”) (Ernster and Schatz, 1981). Research lasting for about seven decades recognized mitochondria as the metabolic and redox hub; and as an independent but cooperating regulatory center for the cell, indispensably important for physiology as well as being involved in numerous pathological states. One of the research milestones included the discovery of a small, but independent, mitochondrial genome; mitochondrial DNA (mtDNA), which also provided support for the endosymbiotic origin of mitochondria (Lang et al., 1999).

The key milestone, leading to the Nobel Prize, explained the mechanism of oxidative phosphorylation (OXPHOS), based on Peter Mitchell's chemiosmotic theory, demonstrating proton coupling between the respiratory chain (RC) and ATP-synthase (Matlin, 2016; Mitchell and Moyle, 1967).

**FIG. 1.**
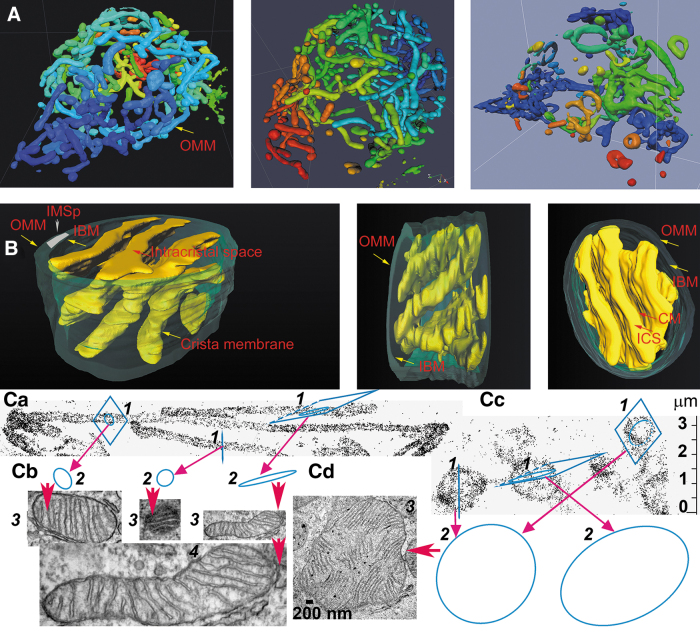
**Hierarchy of mitochondrion structure. (A)** Continuous (*left*), partially (*middle*), and a predominantly fragmented mt network (*right*); **(B)** segment of mitochondrial tubule with crista lamellae; **(C)** origin of TEM images as sections of mt network. For **(A)** 4Pi microscopy images (Plecitá-Hlavatá et al., 2008) of INS-1E cells were taken as examples of intact cells (*left*), cells treated with 20 μ*M* rotenone (*middle*) or 1 μ*M* uncoupler FCCP (*right*). Color coding depicts each individual continuous mt tubules (objects). **(B)** Shows FIB/SEM images of intact HEPG2 cells (Dlasková et al., 2019) and thus illustrates a major topology of mitochondrion: (1) the OMM-IBM cylindrical sandwich with IMS_p_ (its small segment marked with a *white strip*) between the OMM and IBM (*green*); (2) ICS, which is stained together with CMs and proteins residing in them (*yellow*), thus visualizing the crista lamellae; (3) matrix—represented by the free space between ICS, that is, between crista lamellae. **(C)** Shows projections of mitochondrial tubules of HEPG2 cells imaged by 3D PALM **(Ca)** (Plecitá-Hlavatá et al., 2016) or their fragmented spheroids after the treatment with 1 μ*M* FCCP **(Cc)** and explains how TEM sections (*blue*) may arise from them **(Cb, Cc,** respectively**)**. Therefore, when the random sections are perpendicular to the mitochondrial tubule, resulting TEM images of “mitochondria” show nearly *circular objects* **(Ca, Cb)**. When the section to the tubule is tilted, the resulting “mitochondria” are ellipsoidal **(Ca, Cb)**. If the sections are applied to ∼2 μm spheroid fragments, the resulting TEM images display the ∼2 μm “mitochondria” **(Cc, Cd)**. Note that ∼2 μm spheroid fragments can arise from ∼10 μm long mitochondrial tubules due to the instant fission. Within the mt network, such fragments can even be fused with the rest of the mt network. 3D, three-dimensional; CM, crista membrane; FIB/SEM, focused-ion beam/scanning electron microscopy; IBM, inner boundary membrane; ICS, intracristal space; IMS_p_, peripheral intermembrane space; mt, mitochondrial; OMM, outer mitochondrial membrane; TEM, transmission electron microscopy.

Other investigations demonstrated that mitochondria form a *mitochondrion* (Fig. 1A), that is, a nearly entirely connected tubular reticular network in the cell (Bereiter-Hahn et al., 2008; Dlasková et al., 2010; Plecitá-Hlavatá et al., 2008), including skeletal muscle (Glancy et al., 2015) and heart cells (Eisner et al., 2017; Ong et al., 2017).

The mitochondrial (mt)-network dynamics involving fission and fusion is beneficial for maintenance of healthy state, since physiological mitochondria-specific autophagy (mitophagy) is acting on mt-network fragments and eliminates those with low membrane potential (Eisner et al., 2017; Pickles et al., 2018; Twig et al., 2008). The mt-network can be entirely fragmented physiologically (in neuronal axons, upon cell division) or in numerous pathological states. OXPHOS maintenance can protect elongated mitochondria against mitophagy (Gomes et al., 2011).

Last, but not least, when counting milestones in mitochondrial research, recognition of mitochondrion as a redox hub must be mentioned. Superoxide (O_2_^•−^) and/or hydrogen peroxide (H_2_O_2_) formation by RC complexes was revealed to reach a maximum in non-phosphorylating mitochondria (Boveris and Chance, 1973; reviewed in Ježek and Hlavatá, 2005). Superoxide was later found to be produced at more than 11 sites (Brand, 2020; Brand, 2016; Fang et al., 2020; Quinlan et al., 2013). H_2_O_2_ resulting from its dismutation was then implicated in redox signaling (Collins et al., 2012; Diebold and Chandel, 2016; Jezek et al., 2020; Plecitá-Hlavatá and Ježek, 2016).

Typically, the term reactive oxygen species (ROS) is used, when either particular species are unknown, or when dealing with a group of these species. For describing mechanisms, we prefer to name the particular species, that is, mostly we are dealing with either O_2_^•−^ or H_2_O_2_.

### B. From Palade's and Hackenbrock'cristae to mitochondrial cristae dynamics

**FIG. 2.**
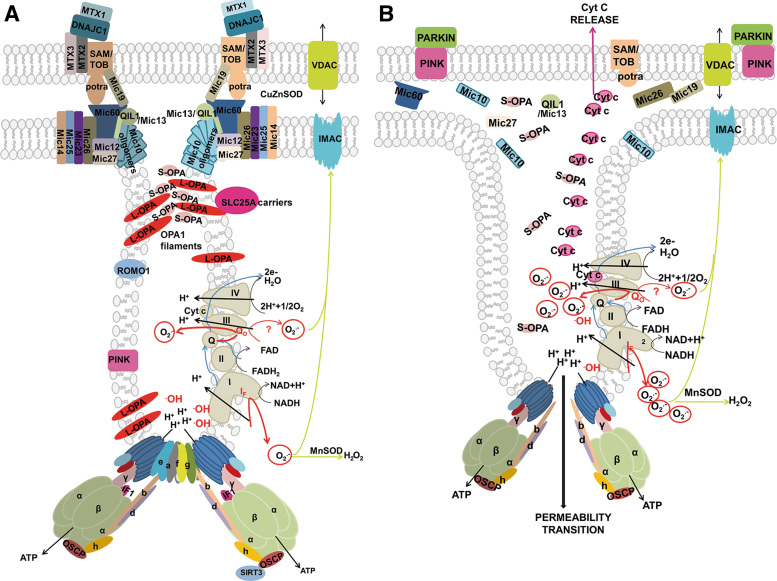
**Schematic 2D view of crista lamela with its forming components. (A)** Proteins of cristae lamella in normal and **(B)** in the apoptotic state (Jezek and Plecita-Hlavata, 2009). The crista junctions are formed by connections of SAM/TOB complex *via* its Potra domain with MICOS complex. The nomenclature of MICOS subunits, abbreviated Mic, includes “Mic” followed by their molecular weight. Various forms of OPA, L-OPA and S-OPA are illustrated, as well as their possible interaction with certain mitochondrial carriers and ROMO1 proteins. RC location in the crista lamela flanks and the position of ATP-synthase dimers in the crista lamela edge is also illustrated, together with the position of PINK kinase. Superoxide diffusion into the cytosol proceeding over the IMAC and porine isoforms (VDAC) is also depicted. On apoptosis initiation **(B)**, the open crista outlets due to disrupted crista junctions (or crista junctions moving apart) cyt ***c*** release is allowed at the parallel PINK relocation. The lack of cyt ***c*** causes increased superoxide formation in the Complex III or other superoxide generation sites and higher diffusion of superoxide/H_2_O_2_ into the cytosol further accellerates apoptotic processes. A hypothesis is depicted, assuming that disordered ATP-synthase dimers and parallel cristae disruption substantiate the phenomenon of permeability transition pore. 2D, two-dimensional; cyt ***c***, cytochrome ***c***; H_2_O_2_, hydrogen peroxide; IMAC, inner membrane anion channel; L-OPA, long OPA; MICOS, mitochondrial cristae organizing system; OPA, optic atrophy; PINK, PTEN-induced putative kinase; RC, respiratory chain; SAM, sorting and assembly machinery; S-OPA, short OPA; VDAC, voltage-dependent anion channel.

The major ultrastructural feature of the mitochondrion is the existence of parallel lamellar cristae, formed by the invaginated inner mitochondrial membrane (IMM) (Fig. 1B, C). Its non-invaginated part, the inner boundary membrane (IBM) forms a cylindrical sandwich with the outer mitochondrial membrane (OMM). Crista membranes (CMs) meet IBM at crista junctions (CJs) formed by the two major complexes: mitochondrial cristae organizing system (MICOS) of IMM connected to sorting and assembly machinery (SAM) complex of OMM (Fig. 2A, B). Cristae dimensions, shape, and CJs are varied with different metabolic regimes and numerous physiological and pathological situations, including apoptosis (Fig. 2B).

The first transmission electron microscopy (TEM) images of isolated mitochondria and the detailed studies of mitochondrial cristae using TEM were reported in the pioneering work of Palade and Sjöstrand in the 1950s (Matlin, 2016; Palade, 1952). The first dynamic status of cristae was observed by Charles Hackenbrock, who noticed changes in cristae folding as a response to the OXPHOS status (Hackenbrock, 1968; Hackenbrock, 1966).

**FIG. 3.**
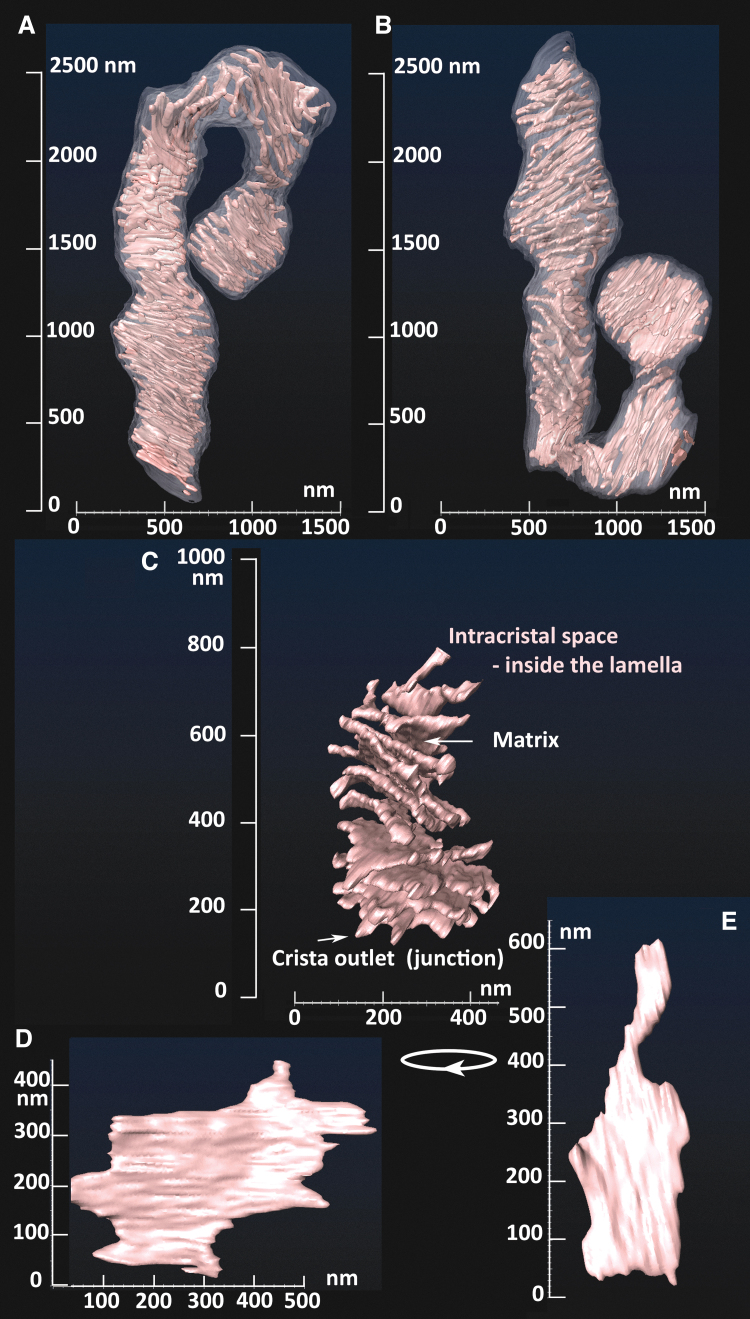
**Crista lamellae in a continuous mitochondrial tubule of INS1E cells.** FIB/SEM 3D images of crista lamellae in ∼4 μm segment of the mitochondrial tubule **(A, B)**, including a selected detail **(C)** and images of a single crista lamella **(D, E)**.

His TEM images showed a condensed cristae conformation upon the transition of isolated rat liver mitochondria to a phosphorylating state 3. In contrast, non-phosphorylating isolated mitochondria (state 4) acquired a so-called orthodox conformation of cristae. The advent of electron microscopy tomography provided three-dimensional (3D) images of cristae (Fig. 3), the lamellar structure of which become apparent in a seminal work by Terry Frey and Carmen Mannella (Frey and Mannella, 2000; Frey et al., 2002; Mannella et al., 1997).

Paradoxically, *in situ* mitochondria exhibit Hackenbrock's orthodox conformation of cristae (Fig. 1C), where a large mitochondrial matrix space pushes apart the IMM up against the OMM with a small (shrunken) intermembrane space (IMS) between them (Frey et al., 2002; Mannella et al., 1997; Perkins et al., 2010; Sun et al., 2007). The IMS portion in cristae (the crista lumen) is then called intracristal space (ICS). The dynamics of cristae and their possible fusion has been suggested (Mannella et al., 2001), and fused cristae have been imaged, whereas in extreme cases of apoptosis (tBID treatment) a single entirely fused cristae reticulum was observed in a 860 nm-mitochondrial spheroid (Mannella, 2008).

Apoptosis initiation accompanied by the cytochrome ***c*** release, indeed, drastically changes cristae morphology (Scorrano et al., 2002; Sun et al., 2007). The lamellar cristae shape was recognized using focused-ion beam/scanning electron microscopy (FIB/SEM) in fixed cells (Kühlbrandt, 2019). The paradigm of dynamic cristae was supported relatively recently (Huang et al., 2018; Kondadi et al., 2020; Stephan et al., 2019; Wang et al., 2019a), demonstrating cristae to be dynamic lamellae by superresolution nanoscopy in living cells, with remarkable results of cristae dynamics with mobile CJs with an OMM travelling up to 50 nm over a time scale of seconds (Kondadi et al., 2020).

### C. Complex topology of mitochondrial network

Scientific tradition still depicts mitochondria as they would be a sample of isolated mitochondria. Since there exists a mt *tubular network in the cell*, nearly entirely interconnected (Fig. 1A) and such a network occurs also in skeletal muscle (Glancy et al., 2015) and heart (Eisner et al., 2017; Ong et al., 2017), one must admit that isolated mitochondria originate from the artificially dissected mt-network. Eventually, a portion could stem from remnants of spheroids already fragmented *in vivo* before cell/tissue homogenization (Dlasková et al., 2019; Tauber et al., 2013).

It is because only upon specific physiological events (cell division, *e.g.*) and pathologies, a complete mt-network fragmentation occurs. A partial mt-network fragmentation reflects shifted balance between network fission (division) and fusion, slightly in favor of fission. Physiologically, mitophagy acts on the mt-network fragments and cannot process long mt network tubules (Twig et al., 2008). Mitophagy acts preferentially on fragments with a low membrane potential, which results mostly from impaired mtDNA-encoded subunits of the RC and ATP-synthase, from severe oxidatively modified proteins with consequently impaired function and from other dysfunction of elements in the particular fragment.

In this case, improvement in mitochondrial quality can be achieved (Twig et al., 2008). When mitophagy and other systems of degradation of mt elements are balanced with mitochondrial biogenesis, a steady state is established. However, both augmented as well as insufficient mitophagy leads to pathological states.

Typically, the predominantly interconnected mt-network has its physiological dynamics, when fusion and fission are in an overall balance (Fig. 1A) (Giacomello et al., 2020). Locally at the given moment, a certain part of the mt-network reticulum undergoes fission; and at the other location, two neighbor mt-tubules just fuse into one. Considering all known aspects of mt-network dynamics, which is beyond the scope of this review, one can recognize as a standard cristae organization the one existing in long linear mt tubules (Figs. 1B and 3). The cristae organization in small spheroid fragments produced by fission is most likely different (Fig. 1Cd) since also the organization of mtDNA in nucleoids therein is different—nucleoids form clusters (Chapman et al., 2020).

Advanced cryo-electron microscopy tomography techniques and FIB/SEM progressed to the visualization of cristae lamellar architecture within rather long segments of cylindrical mitochondrial network tubules (Dlasková et al., 2019). Examples of such FIB/SEM 3D images are seen in Figures 1B and 3. They visualize ICS plus stained intracristal membranes with proteins. Moreover, identical cristae lamellae are recognized by the fluorescence nanoscopy of fixed and even living cells (see Section IV).

The emerging field of cristae dynamics should judge, whether such apparent dynamics in a time scale of seconds is mediated by specific yet unknown proteins; whether it is mediated by different states of optic atrophy 1 (OPA1) filaments (see Section IV), ATP-synthasome, and MICOS modes; or whether it simply occurs as a consequence of molecular dynamics related to mitochondrial biogenesis, being substantiated, for example, by lateral diffusion of incoming nascent synthesized phospholipids, *etc.*

Moreover, cristae biogenesis stems from the influence of both, cristae shaping proteins (see Section IV.B) and spontaneous forces of lipid- and protein-established membrane curvature, acting in concert (Graham and Kozlov, 2010).

The mt-tubules are formed by the OMM, which can be viewed to contain proteins required for mitochondrial integration within cellular signaling (Giacomello et al., 2020). However, this membrane exists in a cylindrical ∼20 nm thick sandwich with the so-called IBM, representing the unfolded portion of IMM (Fig. 1B). The cristae shaping proteins, such as MICOS complexes (Fig. 2A), surround the hollow space, a crista outlet. This is a 12–40 nm pore- or slit-like structure joining the intracristal lumen with the peripheral intermembrane space (IMS_p_) (Perkins et al., 1997). The IMS_p_ is formed by a thin middle aqueous layer between OMM and IBM (green in Fig. 1B).

### D. Significance of mitochondrial ultramorphology and cristae dynamics

**FIG. 4.**
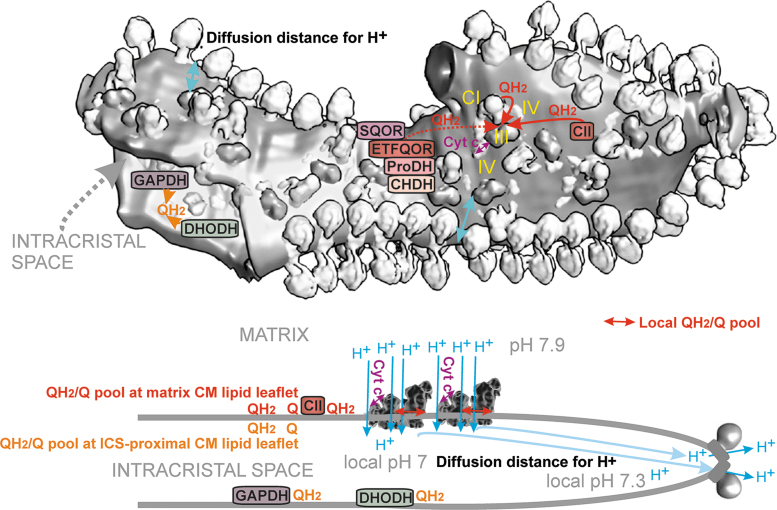
**CoQ diffusion and protonic coupling in single crista lamella.** *Top part*: adopted single crista structure from Nesterov et al. (2021) with structures of RC supercomplexes and ATP-synthase dimeric arrays. The proximal visible surface represents the crista membrane lipid bilayer leaflet oriented toward the matrix (CM_m_). The distances are marked for a minimum path of proton diffusion (*mild blue arrows*) as a coupling entity between the RC proton pumping and the ATP-synthase (see also *bottom part*); and for shuttling of cytochrome ***c*** at the supercomplex surface (*purple arrow*). The distances are also marked for a short QH_2_ diffusion between Complex I and III around supercomplexes (*red arrow*) and much longer QH_2_ diffusion path from Complex II (*red arrow*) to CIII or from CM_m_-located oxidoreductases and DH to CIII (*dashed red arrows*). Inside the broken portion of crista lamella at the CM_ICS_ surface, a QH_2_-diffusion path is indicated by *orange arrows*. Note, that a short QH_2_ diffusion around a supercomplex within the CM_m_-lipid leaflet must be followed by the flip to the CM_ICS_ leaflet to reach the Q-binding site of Complex III (QBS_IIIo_). A simple QH_2_ diffusion within the CM_ICS_ is sufficient for the electron transfer from GAPDH and DHODH to CIII. *The bottom part* illustrates chemiosmotic (proton) coupling inside a single crista lamela. ATP-synthases within dimeric arrays, localized at the edge, receive protons diffusing from the outlets of RC proton pumps, that is, of CI, CIII, and CIV, located within supercomplexes at the flank of crista lamella. Lipid leaflets of the CM are marked. CI, complex I; CIII, complex III; CIV, complex IV; CoQ, coenzyme Q; DH, dehydrogenase; DHODH, dihydroorotate dehydrogenase; GAPDH, glycerol-3-phosphate dehydrogenase; Q, ubiquinone; QBS, Q/QH_2_ (ubiquinone/ubiquinol) binding site; QH_2_, ubiquinol.

The discovered changes in cristae morphology, size, and cristae dynamics should affect not only the efficiency of protonic coupling between the respiratory proton pumping and ATP-synthase (Fig. 4) but also distinct states of superoxide formation since both simultaneously reflect distinct given metabolic fluxes under different physiological and pathological conditions. Coenzyme Q (CoQ), existing in an oxidized form as ubiquinone (Q) and in a reduced form as ubiquinol (QH_2_), provides an essential electron carrier for the mitochondrial RC (alternatively termed the electron transfer chain) and enzymes contributing to or consuming the CoQ pool. Cristae structural organization and the existence of RC supercomplexes or their disassembly affects also superoxide formation.

When simplifying, one can envisage that at longer CoQ diffusion distances (at a delayed diffusion), a higher chance of electron leak to the oxygen exists and hence higher superoxide formation should take place. In general, QH_2_ diffusion from various dehydrogenases (DH) spans longer distances relatively to a short diffusion within/around the RC supercomplexes. Moreover, a single crista (lamella) is specifically organized to contain arrays of the ATP-synthase dimers at its edges, whereas the RC supercomplexes are located in lamella flanks (Fig. 4). Such organization also allows minimum distance for two-dimensional (2D) diffusion of protons within ICS.

Therefore, in this review, we shall discuss first the architecture of RC supercomplexes in relation to the CoQ diffusion and internal electron transfer retarding mechanisms leading to superoxide formation. Enormous recent progress has uncovered mechanisms involved in proton-coupled electron transfer *via* the RC. This calls for reconsideration and more precise determination of the locations of the currently still only phenomenologically defined sites of superoxide formation. Moreover, thresholds for redox burst that substantiates redox signaling should be distinguished from overly excessive superoxide formation that always leads to oxidative stress.

Alternatively, a mild decrease in the active antioxidant mechanism can substantiate redox signaling, whereas a drastic decrease or absence of antioxidant mechanisms produces oxidative stress and its consequences, including the pathologically modified cristae ultramorphology. We need to recognize processes behind a bizarre cristae conformation, such as “onion” like and cristae reticulum and get knowledge, whether such morphology still corresponds to a simple distinct physiological state or not.

In the latter case, we should also recognize when it becomes an origin and when a consequence of pathogenicity. As a physiological one, we can consider situations, when such bizarre morphology could be reversed. We may learn from these investigations, when altered cristae morphology could be a possible marker for pathology and diseases. Mitochondrial science needs more integration, due to the consequences of all known structural and mechanistic details.

The final frontiers in mitochondrial research are concerned with nucleoids of mtDNA and relation of their biology to cristae dynamics. This article aims at reviewing some of these aspects but the research needed to integrate nucleoid biology has not yet been done. It is necessary to stress that due to encoding of the key RC and ATP-synthase subunits by mtDNA, mtDNA mutations and dysregulated mtDNA maintenance and expression typically lead to impaired OXPHOS and enhanced superoxide formation.

In a vicious spiral of events, this leads to oxidative stress and further impairment of mitochondrion components. Since mtDNA encodes subunits forming the entire membrane arm of the RC Complex I (CI), the key subunit of Complex III (CIII), and the key membrane-embedded subunit of the ATP-synthase F_O_ moiety, impaired mtDNA expression leads also to disrupted cristae architecture.

We focus this review on mammalian mitochondria, despite enormous information on mitochondrial structure and function discovered from work with other systems, including fungi, plants, and protozoa. The reason is obvious since it would cover another review article. When mechanistic details concerns with yeast or other lower organisms, we will always note this.

## II. CoQ and RC Supercomplexes

### A. CoQ diffusion within the lipid bilayer membrane

#### 1. CoQ diffusion within phospholipid bilayer

CoQ in an oxidized form as ubiquinone (Q) and in a reduced form as QH_2_ is the prominent essential electron carrier for the mitochondrial RC and enzymes contributing to or consuming the CoQ pool. CoQ is an amphipathic molecule of a modified benzoquinone polar head with a long lipid chain of 6–10 isoprenoid 5-carbon units. In humans, Q10 predominates and contains a 50-carbon tail, consisting of 10 isoprenoid units (Yuan et al., 2021), whereas in rodents Q9 predominates over Q10 (Burger et al., 2020).

The quinone group can be first reduced by a single-electron reduction to a semiquinone radical QH^•^, while acquiring the first proton. Typically, this is followed by the second step, another single-electron reduction, now of QH^•^ to QH_2_, receiving a second proton. The overall two-electron reduction yields QH_2_. The oxidation of QH_2_ reverts it to Q. The precise way in which the overall scheme proceeds is enabled by the specific internal structure of RC complexes, as discussed below.

The location and movement of CoQ within the phospholipid membrane have been extensively studied; however, unequivocal conclusions have not yet been reached. The CoQ-headgroup was reported to be buried at a depth of ∼1.6 nm above the central plane of the lipid bilayer, reaching a position between the third and sixth carbon atom from the carbonyl (Galassi and Arantes, 2015). Its positioning with respect to lateral diffusion at the same level was termed “diving Q” (Hoyo et al., 2017; Söderhäll and Laaksonen, 2001) and was supported by various physical techniques (Afri et al., 2004; Fato et al., 1986; Francisco and Juan, 1985; Jemiola-Rzeminska et al., 1996; Katsikas and Quinn, 1982; Lenaz et al., 1992; Metz et al., 1995; Nerdal et al., 2015; Ondarroa and Quinn, 1986; Samorì et al., 1992).

Alternatively, lateral diffusion was thought to proceed within a bilayer midplane, described by the term “swimming Q” (Hauss et al., 2005; Quirk et al., 2016). The coexistence of both “diving Q” and “swimming Q” was also suggested (Ausili et al., 2008).

Molecular dynamic simulations were performed for single-component phosphatidylcholine (PC) lipid bilayer (Galassi and Arantes, 2015; Söderhäll and Laaksonen, 2001); or accounted for the typical IMM components phosphatidylethanolamine (PE) and cardiolipin [CL; 1,3-bis(sn-3′-phosphatidyl)-sn-glycerol] (Kaurola et al., 2016). Both showed the predominant location of Q/QH_2_-headgroups to be within the plane of phospholipid headgroups and parallel to them. Such a position allows a relatively high hydratation.

The translocation of Q deeper into the hydrophobic bilayer interior was found to be very rapid (Kaurola et al., 2016). The isoprenoid side chain should be extended and packed together with the lipid acyl chains in the bilayer center (Hauss et al., 2005; Metz et al., 1995). For example, in Q10, such a conformation induces an inflexion that bends the terminal part of the side chain. For a “swimming Q” position, its side chain could lay laterally within the membrane plane (Hauss et al., 2005).

Molecular dynamics simulations identified PC to be the major interacting partner for CoQ, which was explained by the bulky character of PC headgroups (Kaurola et al., 2016). However, for the reduced CoQ, the QH_2_-headgroup is thought to locate and migrate much closer to the lipid bilayer surface. This was suggested by studies of the hydrated hexagonal phase of 1-palmitoyl-2-oleoyl-PE, in which QH_2_ headgroups parallelled PE headgroups, whereas Q headgroups were located deeper in the acyl-group region (Wollstein et al., 2015). Detailed knowledge on Q/QH_2_ migration within cristae membranes (CM) is needed to judge from which pool CoQ binding to Q-binding sites of RC complexes occurs since these sites have distinct positional depth in the bilayer.

#### 2. CoQ within cristae membranes

CoQ content in mammalian IMM accounts for 0.5–2 mol% relative to phospholipids (Aberg et al., 1996). The negatively curved inner lipid leaflet in cristae (the one facing ICS) is just enabled by the high overall content of PE (∼49 mol%) and CL (∼6 mol%) (Hovius et al., 1990), and it is scaffolded by specific proteins such as FAM92A1, which binds negatively charged lipids, CL, and phosphatidylinositol 4,5-bisphosphate, stabilizing the negative cristae membrane curvature and hence enabling cristae ultrastructure (Wang et al., 2019b). Thus PE and CL are concentrated in crista tips (Ikon and Ryan, 2017). Their non-bilayer structures formed in the apex of cristae enable and synergize with ATP-synthase dimerization (Gasanoff et al., 2021).

However, the distinction between *in vivo* membranes and experimental phospholipid bilayers lies in the amazingly high protein content of up to ∼80% of IMM dry weight. This accounts for the high content of protein complexes or integral membrane and peripheral membrane proteins. In such membranes, lipids tend to segregate and their dynamics are different, specifically around the proteins in so-called lipid anulli. These are lipids adjacent to integral membrane proteins. Interestingly, the inclusion of CoQ in IMM-mimicking membranes increased lipid packing order and membrane density (Eriksson et al., 2018).

Evaluations using liquid chromatography-tandem mass spectroscopy showed that 90% of CoQ is reduced *in vivo* (Burger et al., 2020). The Q9/Q10 ratio was found to be around 10 in the mouse heart and 42 in the liver. In isolated bovine mitochondrial membranes (predominant Q10) respiring with NADH, the total CoQ pool contained 60% QH_2_. This dropped down to 4% when Complex I was inhibited with rotenone; and down to 12% when succinate was a respiration substrate and the inhibition of Complex II (CII) was induced with malonate (Burger et al., 2020).

In turn, an uncoupler FCCP, which vanishes protonmotive force Δ*p* and stimulates maximum respiration, promoted CoQ oxidation (Burger et al., 2020). The QH_2_/Q ratio was thought to reflect the RC efficiency (Guarás et al., 2016).

CoQ diffusion is not a rate-limiting step for RC electron transfer around/within supercomplexes but is limiting for Complex II and other linked enzymes (Fig. 4). CoQ diffusion is altered upon cristae remodeling and could be affected upon distinct cristae dynamics modes. Electron transfer, such as between Complex I and Complex III, can be regarded as diffusion-coupled. It is not a diffusion-controlled transfer, since CoQ diffusion is probably faster than the RC turnover (Gupte et al., 1984).

Diffusion constants were estimated in the range of 10^−9^ to 10^−6^ cm^2^·s^−1^ by various experimental techniques (Fato et al., 1986; Gupte et al., 1984; Llorente-Garcia et al., 2014) and from molecular dynamics simulations (Galassi and Arantes, 2015; Söderhäll and Laaksonen, 2001). Traveling a distance of 20 nm then requires from 4 μs up to 4 ms. Note that 20 nm is the approximate length of the membrane-buried L-arm of Complex I.

### B. CoQ binding and interaction with RC complexes

#### 1. NADH, CoQ binding, and proton pumping in complex I

**FIG. 5.**
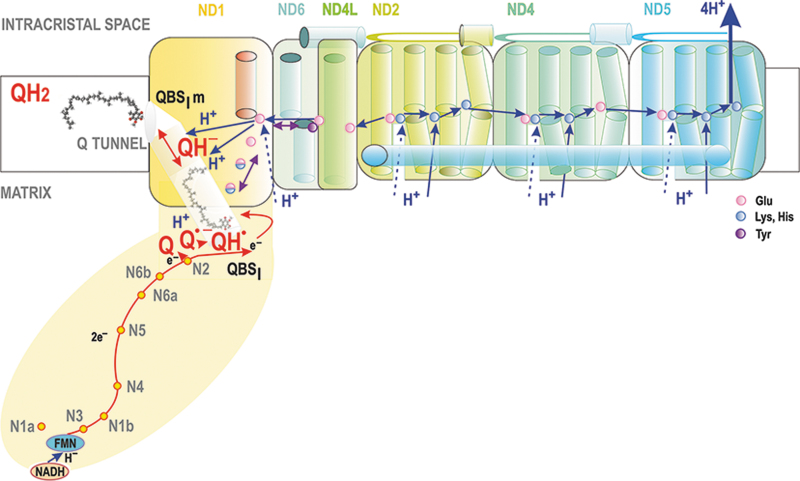
**Coupling between electron transfer and proton pumping in Complex I.** Scheme depicts the Complex I membrane arm, containing ND subunits with major helices and the matrix-exposed hydrophilic arm (*yellow ellipse*) with the indicated flavin FMN site and a chain of iron-sulfur clusters (*red/yellow points*). The *red path* illustrates the electron transfer from the first (N3) to the last iron-sulfur cluster N2 located at the proximity of the CoQ binding site QBS_I_. The latter exists at the end of the Q-tunnel, which spans up to the middle of the cristae membrane (up to the CM_ICS_ phospholipid leaflet). The *blue path* indicates a possible path of protonation/charge propagation within the membrane arm. The Q reduction simultaneously transduces the free energy to proton pumping by the ND5 subunit, while anionic Q^•−^ semiquinone is produced (Kaila, 2021). A *half-blue half-red points* represent HisH^+^ of His38, which was suggested to form an ion pair with proximal Asp160 (Fedor et al., 2017; Wright et al., 2020) or Glu (Nuber et al., 2021) in other resolved structures. Subsequently, the second electron from N2 is supposed to reduce Q^•−^, while Tyr87 and His38 should serve to donate protons to Q, thus forming QH2 (Bridges et al., 2020; Kaila, 2021). A proton transfer from HisH^+^ is predicted to interrupt ion pairing with Asp160, and thus induce conformational changes propagated along a chain of charged AA residues to ND1 (Kaila, 2018; Warnau et al., 2018). Next, the most proximal ND6 undergoes rotation inducing an H-bonded water array between a chain of carboxylates. In this way, protonation is lateraly transferred *via* S-shaped H-bonded water arrays along the whole 20 nm membrane-buried L-arm (Grba and Hirst, 2020; Kaila, 2021; Kampjut and Sazanov, 2020; Röpke et al., 2021). This is enabled by conserved ion-pairs of ND2, ND4, and ND5 “antiporter” subunits, lowering free energy barrier (Grba and Hirst, 2020; Kampjut and Sazanov, 2020). Protonation allows ion-pairing in the neighbour subunit. When this lateral charge propagation reaches the last subunit ND5, a proton is released to the ICS lumen. AA, amino acid; FMN, flavin mononucleotide; HisH^+^, imidazolium. For other details see the Section II.B.1.

Complex I is NADH:ubiquinone oxidoreductase consisting of 45 subunits (∼1 MDa), (Bridges et al., 2020; Fiedorczuk et al., 2016; Galemou Yoga et al., 2019; Jussupow et al., 2019; Kaila, 2021; Röpke et al., 2021; Vercellino and Sazanov, 2022). It has a typical L-shape with the longer 20 nm arm entirely embedded in the membrane and the shorter hydrophilic arm exposed 10 nm into the matrix hydrophilic space (Fig. 5).

The 4 nm-thick membrane arm is formed by ND subunits. The Q-binding site QBS_I_, where the reduction of Q proceeds, is lifted 0.8 nm above the membrane surface still within the hydrophilic arm, therefore it lies ∼2 nm above the ND1 subunit of the membrane arm. (Bridges et al., 2020; Fiedorczuk et al., 2016; Galemou Yoga et al., 2019). QBS_I_ is formed of PSST and 49 kDa subunits (Bridges et al., 2020; Hirst and Roessler, 2016). Notably, CL was found to promote the function of Complex I (Jussupow et al., 2019).

The main branch of the RC electron transfer begins at Complex I. Initially, NADH binds to a cavity containing flavin mononucleotide (FMN) cofactor (Fig. 5). Nicotinamide and flavin rings are oriented to allow a direct hydride (H^−^) transfer (Birrell and Hirst, 2013). The cavity is formed on the hydrophilic arm, exposed to the aqueous space of the mitochondrial matrix. Electrons from NADH *via* FMNH^−^ are transferred over ∼90 μs (Verkhovskaya et al., 2008) *via* the chain of seven FeS clusters throughout the hydrophilic arm to Q at its binding site QBS_I_. The last of the clusters, N2, is positioned 2 nm above the membrane surface and 1.2 nm from QBS_I_ and it donates the electron directly to Q (Fiedorczuk et al., 2016; Kaila, 2021).

Various models, some disputed, were developed for a link between electron transfer and proton pumping. We describe those as cited. First, when anionic Q^•−^ semiquinone is produced (Kaila, 2021), imidazolium (HisH^+^) of His38 was suggested to form an ion pair with proximal Asp160 (Fedor et al., 2017; Wright et al., 2020). Subsequently, the second electron from N2 should reduce Q^•−^, whereas Tyr87 and His38 should serve to donate protons to Q, thus forming QH_2_ (Bridges et al., 2020; Kaila, 2021). Next, proton transfer from HisH^+^ might interrupt ion pairing with Asp160, which would induce conformational changes propagated along a chain of charged amino acid (AA) residues to ND1 (Kaila, 2018; Warnau et al., 2018).

In this suggested way, the Q reduction simultaneously transduces the free energy to proton pumping by ND subunits, encoded by mtDNA (Kaila, 2021; Röpke et al., 2021). Among them, ND2, ND4, and ND5 are phylogenetically derived from ancient Na^+^/H^+^ antiporters. As a result, they can pump, synergically and electrostatically, four H^+^ to the ICS lumen of cristae (Kaila, 2018; Verkhovskaya and Bloch, 2013).

However, the H^+^ output was suggested to only occur *via* ND5, which is the most distant from the hydrophilic arm, whereas ND2 and ND4 redistribute protons toward ND5 (Kampjut and Sazanov, 2020; Vercellino and Sazanov, 2022). The internal coupling is reversible, since QH_2_ can be oxidized, losing two electrons whereas Δ*p* is consumed by the H^+^ backflow (Lambert and Brand, 2004b).

For a long time, it was not known how CoQ, being one of the most hydrophobic biomolecules, can reach its binding site within the Complex I hydrophilic arm, which is raised above the membrane. This has been explained for *Thermus thermophilus* Complex I by the discovery of a Q tunnel, spanning from at least the “diving Q” position in the membrane to the reducing QBS_I_ (Warnau et al., 2018) (Fig. 5). Molecular simulations showed that the CoQ molecule migrates 3 nm in a round trip between the membrane location and the hydrophilic location of QBS_I_ inside this tight tunnel (Warnau et al., 2018).

The Q tunnel is aligned with AA residues that allow motion similar to a piston. Moreover, this piston motion is exergonic, hypothetically continuously providing the energy transduction step from Q reduction toward H^+^-pumping within the 20 nm-long hydrophobic membrane-buried arm. Mechanistically, Q reduction thus ejects QH_2_ from the QBS_I_ to a second Q-binding site buried in the membrane, hereafter termed QBS_Im_, which is formed of aromatic and charged AA residues. They interact with the Q/QH_2_ headgroup while leaving the polyisoprenoid tail within the lipid bilayer (Warnau et al., 2018). Indeed, substitution mutations of certain AA residues of this Q tunnel turned out to block its function (Galemou Yoga et al., 2019).

Importantly, this is the diffusion of CoQ within the Q tunnel that is linked to subsequent conformational changes (Röpke et al., 2021) (Fig. 5). The second Q-binding site QBS_Im_ stabilizes anionic QH^−^ by proton transfer to a glutamate residue (Nuber et al., 2021), which should initiate the long-range proton pumping mechanism *via* S-shaped H-bonded water arrays, laterally along the whole 20 nm membrane-buried L-arm (Grba and Hirst, 2020; Kaila, 2021; Kampjut and Sazanov, 2020; Röpke et al., 2021).

The ND6 membrane α-helix proximal to QBS_Im_ was suggested to undergo rotation inducing an H-bonded water array between a chain of carboxylates to such a chain of ND2, ND4, and ND5 “antiporter” subunits, containing conserved ion pairs, that lower the free energy barrier (Grba and Hirst, 2020; Kampjut and Sazanov, 2020). Protonation of the last element in each subunit would allow ion pairing in the neighbor subunit. In a “closed” state, this is blocked by “middle” Lys residues (Röpke et al., 2021).

Finally, when such lateral charge propagation reaches the last subunit, which is ND5, a proton is released to the ICS lumen and, simultaneously, “closed” states are propagated from ND5 back *via* ND4, then back up to ND2 and QBS_Im_, from which QH_2_ is released. More investigations are still required to confirm the exact structure and dynamic, as well as definitively establish whether the above concepts of a “closed” and “open” state are relevant.

#### 2. Supercomplexes do not enhance CoQ channeling

The RC supercomplexes were predicted, and their structure was subsequently identified in mammalian mitochondria (Lenaz et al., 2016; Letts et al., 2016; Lobo-Jarne and Ugalde, 2018). They may acquire different stochiometries, namely in different organisms, in which they have also distinct structures. Mammalian Complex I associates with and is stabilized by two Complex III structures and one or two Complex IV (CIV; cytochrome ***c*** oxidase, COX) structures into the CI_1_:CIII_2_:CIV_1_ supercomplex or respirasome.

Two Complexes III are attached to the membrane arm of Complex I, at positions proximal to ND1, whereas CIV attaches to the distal part of the CI membrane arm (Lenaz et al., 2016; Letts et al., 2016; Lobo-Jarne and Ugalde, 2018). In the overall supercomplex dimensions, the QBS_I_ lies ∼13 nm from Q-binding sites of Complex III, the “outer” QBS_IIIo_, proximal to the cristae membrane surface facing ICS; and the “inner” QBS_IIIi_, proximal to the cristae membrane surface facing the matrix. Taking the estimated range of diffusion constants, CoQ diffusion over 13 nm would take from 3.6 μs up to 3.6 ms (Fig. 4). Note that QBS_IIIo_ could be identical to or overlap with the site of superoxide formation termed III_Qo_ (see Section II.B.3), whereas, at the QBS_IIIi_, superoxide should not be formed, hence the analogous site III_Qi_ is questionable.

**FIG. 6.**
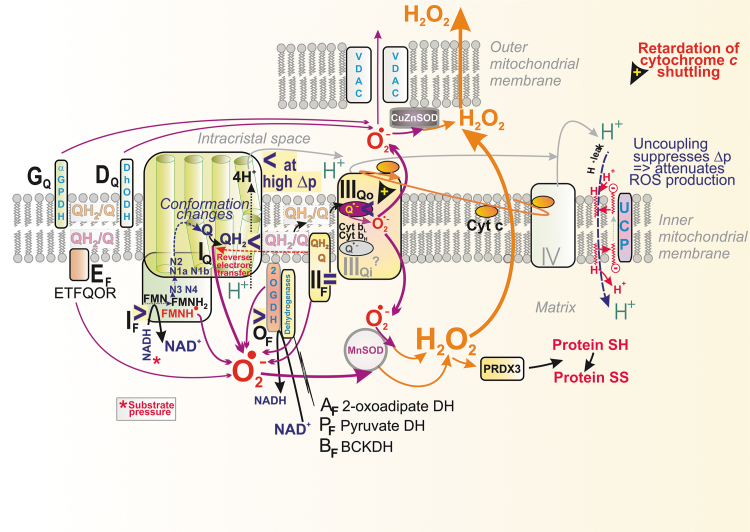
**Superoxide formation within the RC and DH.** Sites of mitochondrial superoxide production named according to the nomenclature of Brand (2016) are illustrated (*black capital fonts*). Predicted relative contribution (relative fluxes) under typical conditions is expressed by the *arrow* thickness for superoxide (*purple arrows*) and for H_2_O_2_ (*orange arrows*). For specification of these conditions in physiology and pathology see Sections II.A, II.B, and VI. Attenuation of superoxide formation is also illustrated when proceeds by uncoupling, enabled by the FA cycling facilitated by the UCP. Also, the RET is depicted, proceeding toward and within Complex I. Another case is shown, when hypoxia, apoptosis, ferroptosis, or certain pathology retards cytochrome ***c*** shuttling (*orange elliptic arrow*), which induces a major superoxide formation at the Complex III site IIIQo. Sites marked as “>” illustrate situations when the excessive forward electron transfer and/or substrate (NADH/NAD^+^) pressure causes superoxide formation, whereas sites marked as “<“depict the opposite, that is, when RET occurs or H^+^ backflow or local retardation within the downstream electron transfer pathway leads to the enhanced superoxide formation. For site II_F_ a mark “ = ” is used, since the maximum superoxide formation proceeds at Km, that is, when succinate concentration approximately equals fumarate concentration. Matrix DH complexes OGDH, PDH, BCKADH, and OADH were suggested to produce superoxide in their forward reactions, when producing NADH, for example, in sites denoted as A_F_ (2-oxoadipate DH), O_F_ (2-oxoglutarate DH), P_F_, (PDH), and B_F_ (branched-chain ketoacid DH, BCKDH) (Brand, 2016; Quinlan et al., 2014). Within each DH complex, distinct E1 subunits ensure specific decarboxylation of the particular oxoacid, and acetylate lipoamide in a thiaminediphosphate-dependent manner. E2 subunits are acyltransferases generating dihydrolipoamide and acyl-CoA. E3 subunits are dihydrolipoamide DH subunits, transferring reducing equivalents of E2-bound dihydrolipoate to FAD of E2 and next to NAD^+^. A charge transfer is established between the protein thiolate anion and FAD (*i.e*., the oxidized form). One may speculate that superoxide might be formed analogously to Complex I I_F_ situation, nevertheless numerous experiments with isolated subunits and assembler complexes, which produce less superoxide, still did not provide a definitive mechanism ( Bunik, 2019; Bunik and Brand, 2018). BCKADH, BCKDH, branched-chain 2-oxoacid dehydrogenase; FA, fatty acid; OGDH, 2-oxoglutarate dehydrogenase; PDH, pyruvate dehydrogenase; RET, reverse electron transport; UCP, uncoupling protein.

**FIG. 7.**
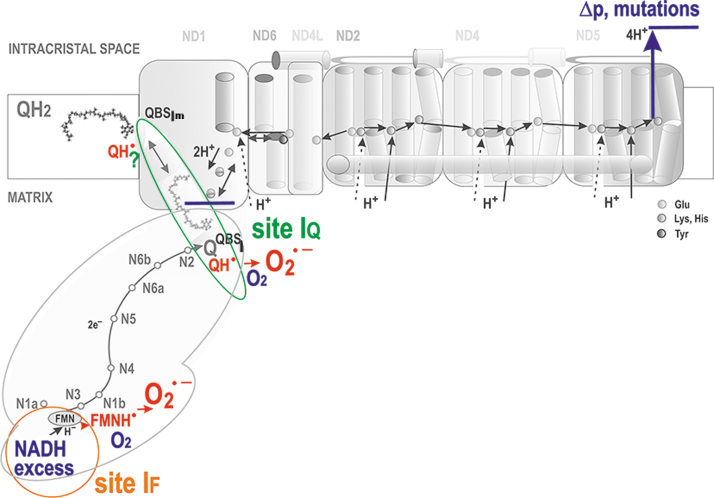
**Major situations of superoxide formation within Complex I.** Probable localizations of site I_F_ (*orange circle*) and site IQ (*green ellipse*) of superoxide formation are shown within the Complex I structure. At a relative NADH excess, even a forward electron transfer allows superoxide formation at the site I_F_, probably by reaction of FMNH^•^ radical with oxygen upon the retarded hydride transfer from NADH. The site I_F_ has also been implicated during the RET (Chouchani et al., 2014; Robb et al., 2018), when the whole Complex I runs backward, and hence superoxide should be formed owing to incoming electron from the N3 iron-sulfur cluster. However, the site I_Q_ was also implicated in RET, as inferred from the inhibitory effect of a specific S1QEL (Brand et al., 2016). Because of the internal coupling mechanisms (see Section II.B.1), retardation of proton pumping (depicted as a *dark blue line*) at high protonmotive force or due to mutant ND subunits inevitably retards the inner charge transfer within the Complex I and therefore sets conditions for superoxide formation, probably at the site I_Q_ (Dlasková et al., 2008). S1QEL, suppressor of site I_Q_ electron leak.

Since the CI Q tunnel ejects QH_2_ to the membrane (to the “diving” level, closer to the matrix surface) (Figs. 5–7), diffusion toward the closely attached CIII should proceed over a similar distance, but terminating with a flip, which must take place from the matrix-proximal lipid bilayer leaflet of crista membrane (CM_m_) to the opposing ICS-exposed leaflet of the bilayer (CM_ICS_). This flip is required to reach the CM_ICS_-positioned QBS_IIIo_ (violet in Fig. 6) within the CIII structure.

The existence of the Q tunnel within CI excluded the original view of CoQ-channeling inside the supercomplex. This was also excluded by experiments using alternative oxidase incorporation (Fedor and Hirst, 2018): CoQ diffusion within or close to annulli lipids, within or in the close vicinity of the supercomplex, was assumed to not exchange with the external distant membrane CoQ pool. This assumption turned out to be incorrect.

Nevertheless, the advantage of supercomplex formation probably lies in the claimed prevention of enhanced superoxide formation (Maranzana et al., 2013) and in the channeling of cytochrome ***c***. Also, the homogenous distribution of complexes in the cristae membrane could be viewed as an advantage (Fedor and Hirst, 2018).

Excluding the above-described “swimming position” for CoQ diffusion, there are two Q/QH_2_ pools, the first one in CM_m_ and the second one in CM_ICS_. In each of these opposing parallel phospholipid leaflets, one may envisage the local Q/QH_2_-pool within and in a proximity to each supercomplex. Definitively, QH_2_ arrives from the Q tunnel right into CM_m_ and somewhere, probably at the lipid/CIII interface, a flip takes place from CM_m_ to CM_ICS_. During so-called reverse electron transport (RET), Complex II, which is distant from supercomplexes, supplies the CM_m_ with QH_2_, which subsequently enters the Q tunnel, initiating reverse Complex I processes, if allowed by the metabolic conditions.

The experiments with alternative oxidase (Fedor and Hirst, 2018) confirmed that neither long-distance diffusion is required nor the inter-supercomplex CoQ migration is essential, but they indicated that an alternative oxidase, after its ectopic expression, can reach the local CoQ CM_m_ pool around supercomplexes.

Recently, even more complex sophisticated supra-structures have been suggested and supported by the obtained cryo-electron microscopy 3D-visualization of a nearly entire crista (Fig. 4). A relatively ordered positioning of supercomplexes in the crista lamella flank was observed to be parallel and positioned below the crista edges, formed by the visualized arrays of ATP-synthase dimers (Nesterov et al., 2021). Distances between the outmost surfaces of F_1_-heads of ATP-synthase and surfaces of CIV or CI were about 5 nm.

This means that proton coupling within the ICS lumen does not need to exceed this distance. These results suggest that in thin crista lamellae, protons do not need to diffuse further than 5 nm when pumped into the ICS lumen by RC supercomplexes and return back *via* the ATP-synthase ***c***-ring, which rotates driven purely by the energy of the H^+^ flux. When cristae inflate to prolong this distance, or even when they form bizarre structures such as a crista network on apoptosis, the energy coupling is less efficient (see Section IV.B.1).

#### 3. Interaction of complex II and complex III with CoQ

**FIG. 8.**
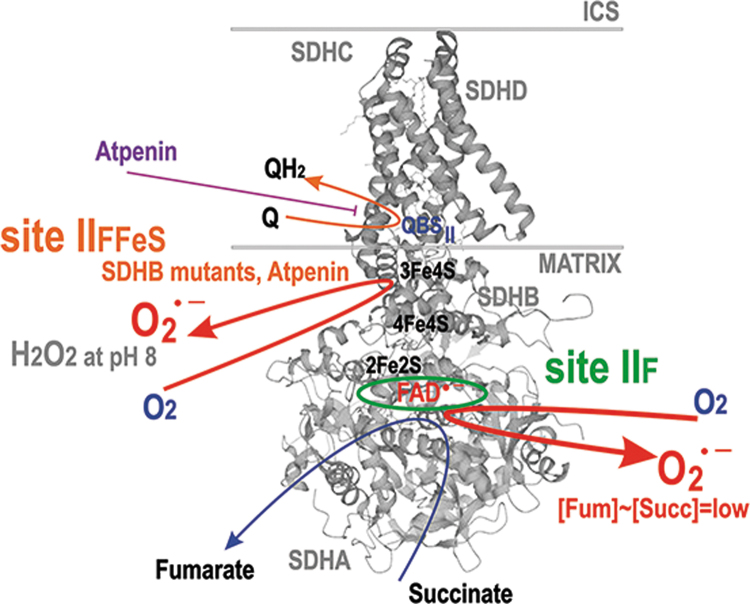
**Major situations of superoxide formation within Complex II.** Probable localizations of site II_FFeS_ (at the 3Fe-4S iron-sulfur cluster) and site II_F_ (*green ellipse*) of superoxide formation are shown within the Complex II/SDH structure. The flavin site II_F_ of the SDHA subunit allows maximum superoxide formation at an optimum lowered succinate next to Km of 100–500 μ*M* for SDH, that is, when this flavin site is less occupied. SDH, succinate dehydrogenase. For details see the Section II.B.3.

The second major route for CoQ is between Complex II and III (Fig. 6). Complex II is succinate:Q reductase, performing oxidation of succinate from the Krebs cycle, coupled to Q reduction *via* FAD with the help of an [3Fe–4S] cluster. Interestingly, no QH^•^ intermediate has been identified for this CII reaction. Complex II or succinate dehydrogenase (SDH) consists of four subunits (Fig. 8). Of the two exposed to the matrix space, SDHA carries the succinate binding site and an FAD-bound flavoprotein, whereas SDHB contains three FeS clusters (Bandara et al., 2021; Bezawork-Geleta et al., 2017).

The hydrophobic subunits SDHC and SDHD anchor the complex to the membrane and contain heme ***b*** at the subunit C/D interface. SDHD forms the CoQ binding site QBS_II_ at the level of the CM_m_ leaflet in the vicinity of the third FeS cluster and heme ***b***. The overall CII reaction oxidizes succinate to fumarate, while electrons from the succinate are transferred to Q. Complex II, thus, acts as the second electron entry point to the RC.

**FIG. 9.**
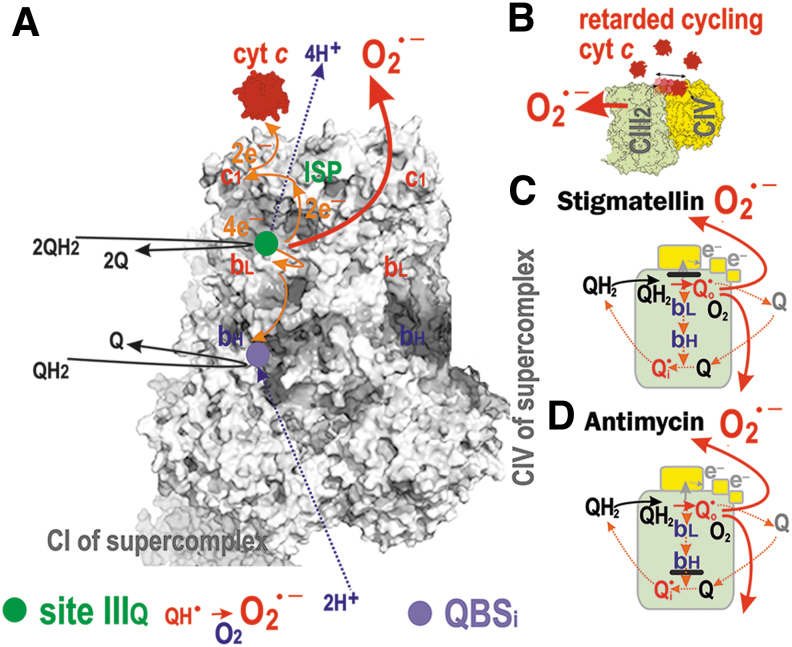
**Major situations of superoxide formation within Complex III. (A)** Localizations of site III_Qo_ (“IIIQ” *green circle*) at the proximity of (or identical with) the Q binding site QBS_IIIo_ (not depicted) and the internal (matrix, CM_m_-located) Q binding site QBS_i_ (*violet circle*) are depicted in the Complex III structure taken as a part of supercomplex. The *gray-scale* dimeric CIII structure including CI and CIV interfaces was adapted from Brzezinski et al. (2021). **(B)** Scheme of retarded cytochrome ***c*** shuttling is illustrated, which leads to superoxide formation at site III_Qo_. **(C)** Situation arising with stigmatellin inhibition, when electron transfer to ISP is blocked, leads also to superoxide formation at site III_Qo_, similarly as in **(D)**, that is, situation with the blocked electron transfer after cytochome ***b***_H_ by antimycin. Antimycin bound close to ***b***_H_, blocks the electron transfer *via* ***b***_L_ plus ***b***_H_ between QB_SIIIo_ and QBS_IIIi_; and therefore stabilizes QH^•^ at QBS_IIIo_. This induces fast superoxide formation at the site III_Qo_ **(D)**. Myxothiazol binds at the entrance of the QBS_IIIo_ pocket interacting with hydrophobic residues and hence prevents the access of Q. Stigmatellin binds to the distal position of QBS_IIIo_ plus interacts by hydrogen bonding with ISP and thus restricts the essential movement of its head domain required for electron transfer to CYC1 **(C)**. Myxothiazol and stigmatellin, each alone stimulate faster superoxide formation by locally slowing the electron flow at the low potential heme ***b***_L_ and toward ISP, respectively. Paradoxically, when added on top of antimycin, they block the antimycin-induced superoxide formation. This is because no QH^•^ can be formed, since the withdrawal of both electrons at incoming QH_2_ is then blocked. Similarly, a S3QEL is acting. It has been developed by chemical biology screens, providing the elegant tool to ascribe the S3QEL-sensitive superoxide formation to the Q_IIIo_ site (Goncalves et al., 2019; Orr et al., 2015). Despite the location of Q_IIIo_ site at the CM_ICS_ level, superoxide was considered to be released about equally to the ICS and matrix aqueous space (Muller et al., 2004; Treberg et al., 2010). The simulation using stigmatellin resembles the enhancement of CIII superoxide formation, which occurs due to retardation of the cytochrome ***c*** cycling between the CIII and CIV (Bleier and Dröse, 2013; Quinlan et al., 2013; Quinlan et al., 2011; Sarewicz et al., 2010) **(B, C)**. CYC1, cytochrome ***c***1 subunit; ISP, iron-sulfur protein; S3QEL, suppressor of CIII Q_IIIo_ site electron leak.

Complex III is QH_2_:cytochrome ***c*** reductase, allowing the so-called Q-cycle (Banerjee et al., 2021). CIII is an obligatory homodimer formed by nine subunits plus one cytochrome ***b*** subunit, encoded by mtDNA (Brzezinski et al., 2021; Fernandez-Vizarra and Zeviani, 2018) (Fig. 9). CIII cytochrome ***b*** harbors two Q-binding sites (hereafter abbreviated QBS_IIIo_ and QBS_IIIi_), each located at the level of the opposing lipid leaflets of the cristae membrane, CM_ICS_ and CM_m_, respectively. Initiating the Q-cycle, QH_2_ is first bound to QBS_IIIo_ close to the ***b***_L_ heme, after its flip from CM_m_ to CM_ICS_.

The conserved histidine and high redox potential 2Fe-2S cluster of the ICS-exposed Rieske iron-sulfur protein (ISP or UQCRFS1) receives the first electron from this incoming QH_2_ and simultaneously passes this electron on to cytochrome ***c*** *via* the c-type heme of the cytochrome ***c***1 subunit (CYC1). This reaction is only possible in the CIII homodimer. Cytochrome ***c*** subsequently diffuses at the cristae membrane surface in the ICS interior toward Complex IV within the same supercomplex, traveling a distance of 10–20 nm.

During the Q-cycle, the second electron from the first QH_2_ molecule is passed through the low redox potential hemes ***b***_L_ (***b***_562_) and ***b***_H_ (***b***_566_) of CIII cytochrome ***b*** (contrary to a high redox potential of ISP) and reaches the second QH_2_ molecule at QBS_IIIi_, forming QH^•^ at QBS_IIIi_. Simultaneously, this is coupled with the H^+^-pumping to the ICS. After the first turn of the Q-cycle, there is a semiquinone radical QH^•^ established at QBS_IIIo_. A proximal site of superoxide formation near to QBS_IIIo_ was termed III_Qo_.

However, the structure surrounding QBS_IIIi_ ensures that QH^•^ is stabilized there without access to oxygen. That is why superoxide formation ascribed to QBS_IIIi_ is still hypothetical. Then, the second round of the Q-cycle recycles QH^•^ at QBS_IIIi_ to QH_2_, which is released to the CM_m_.

Complex II does not provide H^+^-pumping. The character of Complex II as a peripheral membrane protein suggests that Q/QH_2_ diffuses to and from it at the CM_m_, probably at the “diving” depth level. In contrast, in forward RC electron transfer, due to the Complex III Q-cycle, the CM_ICS_ CoQ-pool is drained of QH_2_ and supplied with Q, whereas the CM_m_ CoQ-pool receives QH_2_. It is not known how fast the flipping between CM_m_ and CM_ICS_ is in the native IMM. This flip is relatively fast in model membranes (Kaurola et al., 2016).

#### 4. Cytochrome ***c*** shuttling

Beyond the CYC1 subunit of CIII, electron transfer is not ensured by CoQ, but by another carrier, cytochrome ***c***, a small, water-soluble 12 kDa protein (Nesci and Lenaz, 2021). In the regular crista conformation containing RC supercomplexes, cytochrome ***c*** molecules do not undergo normal 3D diffusion, but instead, 2D diffusion exists inside the ICS lumen, despite it comprising a narrow space of crista lamella interior. Cytochrome ***c*** “slides” on the protein surface, while being attracted by the negatively charged AA residues at the interface between CIII and CIV, as found in yeast (Berndtsson et al., 2020; Moe et al., 2021).

The negative charge then promotes the affinity of the positively charged cytochrome ***c*** for this interface, which allows 2D diffusion. Also, the anionic lipids of CM_ICS_ can provide the required attraction (Chan et al., 2022).

#### 5. Interaction of other enzymes with CoQ

The CM_m_ CoQ-pool is also supplied/used by other proteins (Fig. 4) (Quinlan et al., 2013), such as the electron-transferring flavoprotein ubiquinone oxidoreductase (ETFQOR) (Banerjee et al., 2021; Barbosa et al., 2013; Husen et al., 2019; James et al., 2007; Missaglia et al., 2021; Quinlan et al., 2013; Vergeade et al., 2016; Zhang et al., 2006), proline dehydrogenase (PRODH) (Huynh et al., 2020; Liu et al., 2021), choline dehydrogenase (CHDH) (Salvi and Gadda, 2013), and sulfite:quinone oxidoreductase (SQOR) (Quinzii et al., 2017).

Also, the CM_ICS_ CoQ-pool is employed by glycerol-3-phosphate dehydrogenase (GAPDH) (McDonald et al., 2018) and dihydroorotate dehydrogenase (DHODH) (Bajzikova et al., 2019; Boukalova et al., 2020). Since none of these enzymes were found to cluster with supercomplexes (Burger et al., 2020), one may assume their isotropic distribution within the crista lamellae CM_m_ and CM_ICS_, respectively. Consequently, the lateral CoQ diffusion toward and from these proteins must span much longer distances than CoQ diffusion around/within the supercomplex.

## III. Sites of Mitochondrial Superoxide Formation

### A. Superoxide formation within RC complexes

#### 1. Conditions for superoxide formation in RC sites

Recent progress uncovering mechanisms involved in proton-coupled electron transfer *via* the RC may elucidate some experimental results concerning superoxide formation sites. Detailed knowledge of the steps in RC electron transfer helps to understand the molecular mechanisms of superoxide formation at each type of site. First, let us summarize certain general rules for a superoxide formation site. The classification of superoxide-forming sites is based on the redox isopotential pools that are associated with the reactions, either of NAD^+^/NADH or of Q/QH_2_ (Brand, 2016).

A delay in the electron transfer in a particular segment or site allows electrons to leak to oxygen and form superoxide; or in rare cases, when two electrons subsequently react with oxygen, H_2_O_2_ is directly formed together with superoxide, such as at site II_F_ under specific conditions (Brand, 2016). Besides the Q-linked enzymes (Section II.B.5), matrix DH complexes of 2-oxoglutarate DH (OGDH), pyruvate DH (PDH), branched-chain 2-oxoacid DH (BCKADH), and 2-oxoadipate (OADH) were also reported to produce superoxide in a forward but not reverse reactions (consuming NADH), as evidenced experimentally by studies of isolated mitochondria (Brand, 2016; Quinlan et al., 2014).

There is a local delay in electron transfer during the following three situations at least: (1) *where there is an excessive input* (signs “>” in Fig. 6). This occurs for Complex I when the local concentration of NADH molecules is higher than usual, exceeding the need for the ongoing direct H^−^ transfer. Superoxide is then hypothetically formed at the so-called flavin site I_F_, located in the vicinity of the FMN binding site (Fig. 7).

**FIG. 10.**
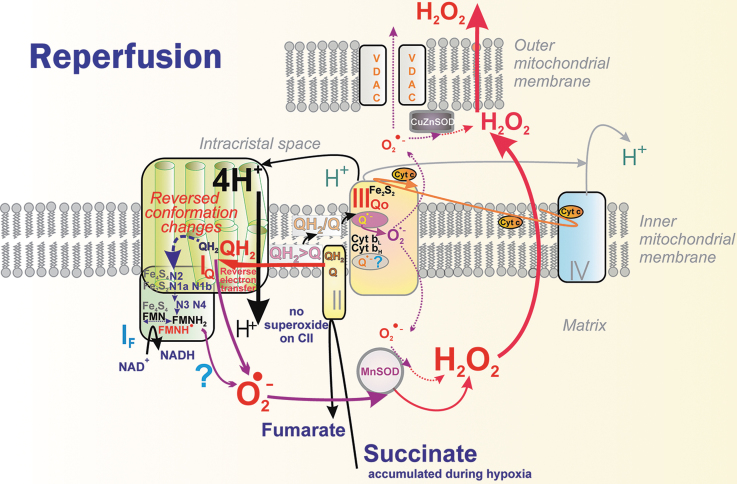
**Superoxide formation on reperfusion following ischemia.** Reperfusion after ischemia in sensitive organs such as the heart causes ischemia/reperfusion injury due to the extremely high superoxide formation during reperfusion, owing to preceding succinate accumulation at ischemia. On reperfusion, succinate is immediately oxidized by CII/SDH while the resulting excessive QH_2_ population causes RET and the entirely reversed Complex I reactions, leading to superoxide formation at either site I_Q_ (Brand et al., 2016) or site I_F_ (Chouchani et al., 2014); or hypothetically at both. CII, complex II.

The principle of an excessive input can also be recognized for superoxide formation in DH. (2) *when output is hindered*, meaning a product-inhibition slows down the preceding electron transfer. This should notably occur in the vicinity of QBS_I_ and QBS_IIIo_ in the superoxide formation sites termed I_Q_ and III_Qo_ (signs “<” in Fig. 6 and Fig. 9A, C, D), respectively (Hirst and Roessler, 2016; Orr et al., 2015; Quinlan et al., 2013; Vinogradov and Grivennikova, 2016). For example, an ongoing inhibition by a product (QH_2_), perhaps already acting within the above-described Q tunnel, was suggested to occur (Brand, 2016) in RET from OH_2_ (the product), back to Q, and in the interior of the electron-transfer pathway within Complex I, which runs backward, including the reversed proton pumping from ICS back to the matrix (Fig. 10).

A specific situation arises in Complex II when at optimum, but relatively low succinate, the site termed II_F_ induces superoxide. But it is not formed at high or lower concentrations (Fig. 8). Finally, there is a delay in electron transfer (3) when proton pumping is initially retarded, such as by Complex I (Figs. 6 and 7). Because of the internal coupling mechanisms (see Section II.B.1), this inevitably retards the inner electron transfer within Complex I (Dlasková et al., 2008). Vice versa, when directed forward, the electron transfer essentially drives the H^+^ pumping (Hirst and Roessler, 2016).

Nevertheless, as for specific physiological or pathological situations, there is no consensus on which sites *in vivo* superoxide/H_2_O_2_ is generated, and further research is required. No consensus has been found, for example, even for the site of superoxide/H_2_O_2_ generation by RET during reperfusion after heart ischemia, which causes ischemia/reperfusion (IR) injury. Either site I_F_ (Chouchani et al., 2014; Robb et al., 2018) or I_Q_ (Brand et al., 2016) is implicated, though the participation of site I_Q_ is supported by the action of specific suppressor of site I_Q_ electron leak (S1QEL). Site I_F_ is suspicious since reversed proton pumping occurs.

#### 2. Superoxide formation dependence on proton pumping

The internal coupling mechanism (Section II.B.1) predicts that the overall rate of electron transfer must be feedback regulated by the protonmotive force (Δ*p*), established by proton pumping by CI, CIII, and CIV (Δ*p* = −Δ*Ψ*_m_ + 2.3*RT*Δ*pH*/*F*) (Jezek et al., 2018; Jezek et al., 2014). This is also implied by ND5 subunit mutations, which, by disabling proton pumping, also lead to increased superoxide formation and subsequent ROS-induced apoptosis (Singh et al., 2017).

In three situations, outlined earlier, there is faster superoxide formation by RC in a non-phosphorylating state, in which no ATP synthesis takes place. This state was historically termed state 4 for isolated mitochondria in an ADP excess. In cells, one can consider the existence of dormant or semi-phosphorylating states with a zero or low intensity of ATP-synthesis. In state-4 or similar, the maximum protonmotive force Δ*p* is established, as well as the maximum IMM electrical potential, Δ*Ψ*_m_ (Jezek et al., 2018; Jezek et al., 2014). This maximum is established because the ATP-synthase does not consume Δ*p* (Δ*Ψ*_m_) under these conditions.

In contrast, in the phosphorylating state, termed state 3 for isolated mitochondria, Δ*p* (Δ*Ψ*_m_) is consumed and used by the ATP-synthase to rotate its membrane subunit ***c***-ring, providing the synthesis of ATP (Davies et al., 2012; Davies et al., 2011; Gu et al., 2019; Guo et al., 2017; He et al., 2018; Kühlbrandt, 2019; Paumard et al., 2002; Rieger et al., 2021; Spikes et al., 2021; Spikes et al., 2020). As a result, the H^+^-pumping is faster, also enabling faster electron transfer and faster respiration, which consequently determines the slower superoxide formation.

In other words, the phenomenon of so-called respiratory control, describing feedback retardation of H^+^-pumping at higher Δ*p* (Δ*Ψ*_m_), slows down the electron transfer and therefore allows faster superoxide formation.

Since a small shortcircuiting of Δ*p* (Δ*Ψ*_m_) is inevitably also provided by an H^+^-leak, considered as a general H^+^-backflow from ICS to the matrix, respiration during the state-4 in isolated mitochondria and dormant low semi-phosphorylating states in the cell is just as fast as allowed by the H^+^-leak. This basal H^+^ conductance exists in proteinaceous lipid membranes.

Nature has designed proteins during evolution, which provide the additional regulated H^+^-leak enabled by free fatty acids (FAs). The FAs serve as cycling co-factors of mitochondrial uncoupling proteins (UCPs, five isoforms) (Jezek et al., 2018), adenine nucleotide translocases (ANTs, ADP/ATP-carrier 3 isoforms) (Bertholet et al., 2019), and some other proteins of the SLC25 family of mitochondrial anion carriers. The FA-dependent activity providing the additional regulated H^+^-leak is termed mild uncoupling.

It is typically activated, when nascent FAs are suddenly recruited to IBM or CM (Fedorenko et al., 2012), such as after their cleavage by mitochondrial phospholipases PNPLA8 and PNPLA9 (Jabůrek et al., 2021; Ježek et al., 2015; Průchová et al., 2022). Mild uncoupling allows fractionally faster proton pumping, inducing slightly faster electron transfer and respiration; and hence slower superoxide formation. Unless a crippled Complex I exists in the mitochondrion, as in pathologies when Complex I subunits encoded by mtDNA are mutated, mild uncoupling attenuates mitochondrial superoxide formation (Jezek et al., 2018).

Since PNPLA8 is redox-activated, a feedback loop exists: a transient burst of elevated superoxide formation (transferred by superoxide dismutase MnSOD into the H_2_O_2_ burst) activates PNPLA8, which induces mild uncoupling in such an antioxidant synergy with ANTs or UCPs (Jabůrek et al., 2021; Ježek et al., 2015; Průchová et al., 2022). This synergy, thus, may return the originally elevated superoxide formation to a “normal” steady state.

#### 3. Superoxide formation at flavin site I_F_ and site I_Q_ of complex I

The I_F_ site is located close to iron-sulfur clusters N1a and N3. Reduction of the N1a FeS cluster increases the affinity for NAD^+^ binding, which persists until electrons reach N2 and QBS_I_ (Saura and Kaila, 2019). This fact itself would predict that the “normal” forward Complex I function should not produce any significant superoxide. In contrast, analysis of model simulations suggested that sites I_F_ and I_Q_ should produce similar amounts of superoxide during RET (Bazil et al., 2014), as confirmed experimentally, though in isolated mitochondria (Treberg et al., 2011). These results determined that there must be two distinct sites of superoxide formation in Complex I (Fig. 7).

It was observed with the isolated Complex I that the I_F_ site provides more superoxide at a higher NADH/NAD^+^ ratio (Hirst et al., 2008; Kussmaul and Hirst, 2006), meaning at a high substrate pressure. Only Complex I molecules with reduced flavin and without any NADH and NAD^+^ were found to form superoxide (Hirst et al., 2008; Kussmaul and Hirst, 2006). To introduce Δ*p* into studies, submitochondrial particles were employed (King et al., 2009; Pryde and Hirst, 2011). When Δ*p* was set to zero and Q reduction was prevented, results were equal to those with the isolated Complex I.

When Q reduction was permitted, superoxide formation was slower relative to acting Q reduction, but inhibitors of QBS_I_ did not stimulate it (King et al., 2009; Pryde and Hirst, 2011).

In conditions of higher NADH/NAD^+^ ratio, after the direct H^−^ transfer between NADH and FMN, superoxide can be formed at I_F_ site as follows. Without passing the electron to the FeS chain, the NAD^+^ binding is not persistent, and thus the paired FMNH^−^ and NADH form flavosemiquinone radical FMNH^•^ (Ohnishi et al., 2010). Its reaction with oxygen allows superoxide to be produced at I_F_, unlike at low NADH when the pairing of NAD^+^and FMNH^•^ occurs (Hirst and Roessler, 2016).

It is not known how often the above conditions exist in the mitochondrion. Experiments with isolated skeletal muscle mitochondria rather showed that the majority of superoxide was formed by OGDH and PDH (Goncalves et al., 2015; Quinlan et al., 2014). A rather low contribution of I_F_ to superoxide formation was also suggested by experiments when Complex I was depleted and the resulting drop in superoxide formation was negligible (Chinta et al., 2009).

Phenomenologically, product-inhibition of DH by NADH is equivalent to an excessive input of NADH acting at Complex I. Moreover, an optimum substrate pressure, defined as NADH/NAD^+^ ratio, must exist, hypothetically determining the minimum superoxide formation. This is because the order of magnitude for NADH/NAD^+^ ratio was found to be as low as ∼0.01. Ratios were determined from mitochondrial metabolomics studies, showing a remarkable “shortage” of NADH due to its utilization.

In isolated mitochondria, a clear identification of two sites I_F_ and I_Q_ within Complex I during RET has been reported (Treberg et al., 2011). In contrast, for forward electron transport, it is more difficult to recognize the contribution of site I_Q_ to superoxide formation (Lambert and Brand, 2004a). This is now possible after the development of specific suppressor(s) of site I_Q_ electron leak, S1QELs, which do not affect the regular electron transfer *via* RC and inhibit neither ATP synthesis nor metabolism (Goncalves et al., 2019).

In any case, superoxide formation at loci of phenomenologically defined site I_Q_ must depend on Δ*p* since any Q/QH_2_ movement within the Q-tunnel is coupled to proton pumping (Dlasková et al., 2008). On RET, Δ*p* is established by CIII and CIV, whereas CI dissipates this Δ*p*, so superoxide formation depends on this specifically set Δ*p* even more strongly.

#### 4. Conditions for superoxide formation within complex III

The situation (2) of a *hindered output* is also established when cytochrome ***c*** turnover is delayed (Fig. 9B), causing feedback-inhibition of the Q-cycle within CIII, with inevitable superoxide formation at site III_Qo_ (Brand et al., 2016). This proceeds because of the longer transient existence of QH^•^ at the “outer” Q-binding site QBS_IIIo_ (CM_ICS_-exposed) and oxygen diffusion into this site (Fig. 9A) (Husen et al., 2019). Despite the superoxide formation rate slightly increasing, the percentage of produced superoxide decreased with increasing activity of the isolated bovine CIII (Pagacz et al., 2021).

When cytochrome ***c*** was lowered, increasing superoxide resulted from the incomplete or delayed reaction at QBS_IIIo_ given by the established dynamic equilibrium between QH^•^ at QBS_IIIo_ and ***b***_H_, so also relative to ***b***_L_. The *Cytbc*1 mutants from *Rhodobacter capsulatus* were found, where spin-spin interaction between QH^•^ and FeS of ISP was blocked and exhibited an enhanced superoxide release. In mutants, for which such spin-spin interaction existed, there was negligible superoxide generation (Bujnowicz et al., 2019). A role of such a charge transfer was also theoretically simulated (Salo et al., 2017).

Artificially, superoxide is induced by antimycin (Fig. 9D), myxothiazol, and stigmatellin (Fig. 9C). The delayed electron transfer from ISP toward cytochrome ***c*** results in the elevated superoxide formation at the site III_Qo_. Importantly, this production is not attenuated by uncoupling. *In vivo*, such a natural slowdown of the Q-cycle occurs at hypoxia (see Section V.B.2) and was found in mice with Complex IV mutations (Reichart et al., 2019).

**FIG. 11.**
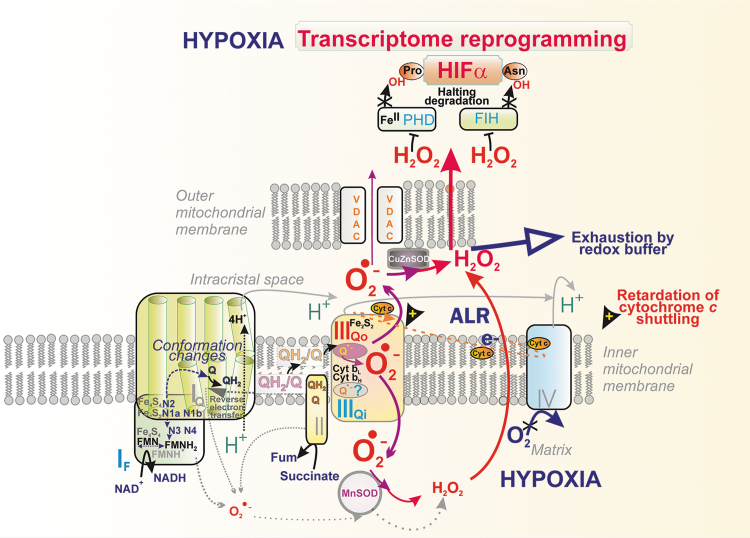
**Superoxide formation in hypoxia.** Scheme depicts steps following the initiation of hypoxic superoxide burst at Complex III site III_Qo_. Such redox signaling leads to or contributes partially to hypoxic transcriptional reprogramming. The site III_Qo_ participation was inferred from the effects of the ablation of Complex III subunits (Bell et al., 2007; Chandel et al., 2000; Chandel et al., 1998; Comito et al., 2011; Guzy et al., 2005; Sabharwal et al., 2013; Schroedl et al., 2002; Waypa et al., 2010) and could be mimicked as a normoxic HIF activation (Patten et al., 2010). Thus, the ablation of Rieske ISP stabilized HIF (Brunelle et al., 2005) and, in turn, suppressors of S3QELs prevented the HIF response (Orr et al., 2015). One may also speculate on theoretical components of a redox buffer that is being dissipated during up to 4-h lag between the onset of hypoxia and redox signaling (maximum HIF stabilization). Thus, MIA40 (not depicted) and the ALR (Jezek and Plecita-Hlavata, 2009) can ensure this. Oxidized ALR has been reported to be regenerated simply by oxygen, but at hypoxia, when this is not possible, ALR may donate electrons to cytochrome ***c***. This would effectively retard the electron transfer from Complex III to Complex IV, and so elevate the site III_Qo_ superoxide formation. Alternatively, due to a partition coefficient of O_2_ in the lipid bilayer of ∼4, the IMM-dissolved oxygen should be exhausted first before a slowdown of the Complex IV (cytochrome ***c*** oxidase) reaction. Again, the resulting slowdown of the cytochrome ***c*** cycling would inevitably elevate site III_Qo_ superoxide formation. The situation can be even more complex due to Complex I inactivation by acute hypoxia (Hernansanz-Agustin et al., 2017). ALR, augmenter of liver regeneration; FIH, factor inhibiting HIF; HIF, hypoxia-inducible factor; IMM, inner mitochondrial membrane; MIA40, mitochondrial intermembrane space import and assembly protein 40; PHD, prolyl hydroxylase domain.

Speculatively, at hypoxia, an IMS protein termed an augmenter of liver regeneration (ALR) (Gandhi et al., 2015) donates electrons to the reduced cytochrome ***c***, which competes at Complex IV with that one produced by Complex III. Again, this can be regarded as inhibition of Complex III by its product, that is, by the reduced cytochrome ***c*** (Fig. 11). Another type of retardation of the cytochrome ***c*** cycling exists at any partial escape of cytochrome ***c*** from ICS, such as occurs on apoptosis (Section VI.A.1).

#### 5. Conditions for superoxide formation within complex II

In isolated mitochondria at high succinate concentrations, Complex II or SDH does not form superoxide at any significant rate (Grivennikova et al., 2017; St-Pierre et al., 2002). At lowered succinate concentration to K_m_ of 100–500 μ*M* for SDH, a flavin site of the SDHA subunit is less occupied, consequently allowing maximum superoxide formation rate at the site termed II_F_, whereas further succinate decrease again diminishes superoxide production (Perevoshchikova et al., 2013; Quinlan et al., 2012a; Trewin et al., 2019).

When the SDHD subunit is blocked to prevent electron transfer to Q, SDH switches to produce ∼70% H_2_O_2_ at pH 8, since three FeS clusters provide a chance for the two-electron transfer to oxygen (Fig. 8) (Siebels and Dröse, 2013). Thus, H_2_O_2_ and superoxide are formed by SDH, when FAD is reduced, but the succinate binding site is not occupied (Hadrava Vanova et al., 2020). Also, an elaborated theoretical model showed that 3Fe-4S iron-sulfur cluster is the primary superoxide source (here arbitrarily ascribed to site II_FFeS_) (Manhas et al., 2020).

### B. Mitochondrial superoxide formation and cell metabolism

#### 1. Dependence of superoxide formation on metabolism

Unfortunately, there is no general rule for the metabolic dependence of superoxide/H_2_O_2_ formation on metabolism and vice versa. Nevertheless, certain common features are likely shared by cells relying on OXPHOS, whereas distinct relationships exist for dormant OXPHOS, such as in cancer cells with specific metabolism. The unifying factor for RC and OXPHOS regulation is likely the ADP/ATP ratio that corresponds to metabolic needs for ATP synthesis (Küster et al., 1976; Meyrat and von Ballmoos, 2019). Less ATP reciprocally requires higher requirements.

Consequently, respiration is higher at higher ADP/ATP, which is manifested as a phosphorylating state (state-3 in isolated mitochondria). The other two most important RC regulators are NADH and succinate availability. Upstream of these two electron donors is the availability of nutrients. OXPHOS and glycolysis are mutually regulated. Typically, higher mitochondrial superoxide/H_2_O_2_ production exists in glycolytic cells (Plecitá-Hlavatá et al., 2015).

To access maximum superoxide formation when conditions of excessive particular substrates for given DHs were set in respiring isolated skeletal muscle mitochondria, OGDH (site O_F_), PDH (site P_F_), and branched-chain 2-oxoacid dehydrogenase (BCKDH; site B_F_) released H_2_O_2_ in rates eightfold, fourfold, and twofold higher, respectively, than the site I_F_ (Quinlan et al., 2014). See Figure 6 for site nomenclature.

Mitochondria respiring with glycerol 3-phosphate produce superoxide at sites I_F_, III_Qo_, II_F_, and G_Q_ (Orr et al., 2015; Quinlan et al., 2013), but when myxothiazol was additionally present, site III_Qo_ cannot produce superoxide hence the major contribution to its formation comes from sites G_Q_ and II_F_ (Orr et al., 2012).

With malate plus rotenone, both sites I_F_ and OGDH (site O_F_) contributed to superoxide formation (Quinlan et al., 2014). With glutamate plus malate, superoxide formation at site I_Q_ was nearly zero, whereas site I_F_, site III_Qo_, and site O_F_ were major contributors (Quinlan et al., 2013; Quinlan et al., 2012b; Slade et al., 2017). This was confirmed when mimicking resting *versus* exercise conditions for skeletal muscle, while sites I_Q_ and II_F_ accounted for 50% of superoxide/H_2_O_2_ formation at rest, the site I_F_ dominated in exercise conditions (Goncalves et al., 2015). About 0.1%–0.5% of the total electron transfer reacted with oxygen to form superoxide (Goncalves et al., 2015). In C2C12 myoblasts, site I_Q_ accounted for 12% and site III_Qo_ for 30% of superoxide/H_2_O_2_ formation, but after differentiation into myotubes site I_Q_ fraction was 25% and contributed to much higher superoxide release (Goncalves et al., 2019).

In model cultured cells, mitochondrial H_2_O_2_ formation (∼30% of total cell H_2_O_2_ production, of which ∼60% was by NADPH oxidase [NOX]) originates about equally from sites I_Q_ and III_Qo_, whereas superoxide release to the matrix accounted for ∼70% contribution from site I_Q_, whereas the remaining 30% was mostly from site III_Qo_ (Fang et al., 2020). These figures stem from the fact that the tested cell lines are derived from cancer cells having specific metabolism.

In contrast, in INS-1E cells, capable of maximum OXPHOS and insulin release at 25 m*M* glucose, on average 60% of overall superoxide released to the matrix comes from the site III_Qo_, whereas site I_Q_ had a negligible contribution (Plecitá-Hlavatá et al., 2020). Moreover, at resting state with low OXPHOS and no insulin release at 3 m*M* glucose, when overal superoxide formation was ∼2.5 higher, site I_Q_ accounted for 25% and site III_Qo_ for 20%. In *Drosophilla* and mice, experiments with suppressor of site III_Qo_ electron leak (S3QEL) revealed site III_Qo_ as a cause for diet-induced intestinal barrier disruption (Watson et al., 2021).

Simple predictions of redox homeostasis are also complicated by the redox-inactivations at higher superoxide burst, the aconitase being well well-known for this. For example, SDH is also inactivated at excessive redox stress in the heart or hepatic steatosis, both induced by SOD2 ablation in 6-day-old mice. These effects were prevented by S1QEL derivatives S1QEL352 and S1QEL712, but not by S3QEL941, RET was implicated as the main superoxide source (Wong et al., 2021). A similar effect was observed in clinical settings (Piao et al., 2020).

Another refinement of rules for *in vivo* redox homeostases should take into account so-called substrate channeling, enabled, in fact, by the cristae. Exemplified for the heart, it was suggested that the 2OGDH complex channels NADH directly toward nicotinamide nucleotide translocase (NNT) and thus does not contribute to excessive superoxide formation at site I_F_, while the resulting NADPH formation by NNT rather contributes to antioxidant mechanisms (Wagner et al., 2020).

#### 2. Excessive superoxide formation induced by reactions of other oxidoreductases

**FIG. 12.**
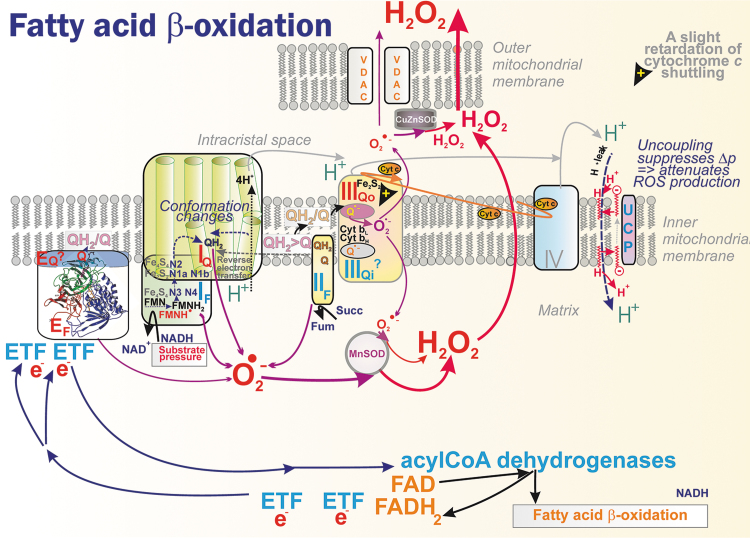
**Hypothetical superoxide formation on FA β-oxidation.** Key sites considered to contribute to the increased superoxide formation. Products of FA β-oxidation are FADH_2_, as reduced FAD by acyl-CoA dehydrogenases; NADH, as reduced NAD^+^, produced by 3-hydroxyacyl-CoA dehydrogenase; and acyl-CoA, shorter by 2 carbons in each cycle. Two ETF with their FADH_2_ cofactors migrate by diffusion in the matrix to the CM surface and transfer electrons to ETF-ubiquinone oxidoreductase, ETFQOR, a protein peripheral to CM_m_. ETFQOR subsequently reduces Q. Theoretically, all these inputs might contribute to likely excessive superoxide formation upon FA β-oxidation. Of course, this depends also on a type of parallel metabolism. ETF, electron-transferring flavoproteins; ETFQOR, electron-transferring flavoprotein ubiquinone oxidoreductase.

The CoQ pools within cristae lamellae CM_m_ and CM_ICS_ integrate the other enzymes with RC (Section II.B.5). Consequently, not only metabolism affecting the Krebs cycle turnover and NADH plus succinate supply to RC, but also metabolic pathways, in which these IMM oxidoreductases are involved, affect mitochondrial superoxide formation. We first illustrate this using the example of FA β-oxidation (Fig. 12).

In isolated skeletal muscle mitochondria slowly respiring with palmitoylcarnitine 44% of rather low H_2_O_2_ production was ascribed to site II_F_ and 34% to site I_F_ (Perevoshchikova et al., 2013). When carnitine was present, accelerating palmitoylcarnitine uptake, a 33% share was found for each of the sites I_F_, II_F_, and III_Qo_. When malate was also added, the contribution of site III_Qo_ increased to 75%, site I_F_ share was 25%, and II_F_ share zero.

However, with carnitine plus myxothiazol, inhibiting Q-cycle and thus keeping CoQ reduced as QH_2_, 50% of H_2_O_2_ production was from site II_F_ due to reversible reaction but the remaining 50% was ascribed to ETFQOR or electron-transferring flavoproteins (ETF) system, to flavin site E_F_, but not to a theoretical site E_Q_. It is necessary to estimate these contributions *in vivo*.

One may analogically expect a complex analysis of sites forming superoxide when significant metabolic fluxes exist in parallel such as for all other enzymes contributing to/consuming QH_2_/Q pool together with RC. For example, CHDH may contribute to superoxide formation as shown by choline induction of superoxide formation in liver mitochondria (Mailloux et al., 2016).

Similar considerations could be done for other oxidoreductases being peripheral to CM_m_, as well as for those peripheral to CM_ICS_, such as GAPDH and DHODH. Indeed, there are numerous reports evidencing increased superoxide formation ascribed to GAPDH (Brand, 2016; McDonald et al., 2018; Orr et al., 2012; Perevoshchikova et al., 2013; Quinlan et al., 2013) and DHODH (Boukalova et al., 2020; Brand, 2016; Orr et al., 2012; Quinlan et al., 2013).

#### 3. Reverse electron transport

RET was found to be a driver of mitochondrial superoxide formation on reperfusion after preceding ischemia, which does accumulate succinate in ischaemic murine brain, kidney, liver, and heart in an n-butylmalonate-sensitive manner (Fig. 10) (Chouchani et al., 2014). Already during 5 min of reperfusion, the accumulated succinate was consumed back to ordinary levels. ^13^C-glucose and ^13^C-palmitate follow-ups had shown that their contribution in the ischemic heart was low, unlike ^13^C-aspartate, which incorporated substantially into succinate during ischemia.

Hence, the reverse SDH/CII reaction was found as a major contributor to ischemic succinate accumulation. This reaction keeps mitochondrial RC proton pumping and ATP production dependent on fumarate, aspartate, and malate since the malate/aspartate shuttle maintains a high NADH/NAD^+^ ratio during ischemia and AMP-dependent activation of the purine nucleotide cycle provides fumarate.

At the onset of reperfusion, SDH/CII instantly oxidizes accumulated succinate (or added dimethylsuccinate) and this causes n-butylmalonate-sensitive RET including the RET throughout the entire Complex I electron path up to site I_F_ (Fig. 10) (Chouchani et al., 2014). This was reflected by the suppression of NADH oxidation. RET was also proven *in vivo* using MitoB probe and *via* aconitase sensitive to damage by superoxide (Chouchani et al., 2014).

A follow-up study in isolated heart mitochondria demonstrated an exponentially increasing RET dependence on increasing Δ*p*, and overall IMM redox states (but not local), that is, S-shape increasing QH_2_/Q and linearly increasing NADH/NAD^+^. RET was also proportional to oxygen concentration (Robb et al., 2018). Authors of this study preferred the interpretation that the site I_F_ is the superoxide source during RET, since they did not admit direct oxygen access to the I_Q_ site.

The development of suppressors of electron leak, particularly at site I_Q_, brought evidence that the site I_Q_ should also produce superoxide upon RET (Brand et al., 2016). Thus, in the Langendorff-perfused mouse heart subjected to ischemia-reperfusion injury, S1QEL1.1 mediated the post-ischemic recovery of cardiac function. S1QEL also improved cardiogenic shock following asystolic cardiac arrest and was suggested for use in practical therapy for improving sudden cardiac arrest (Piao et al., 2020).

Besides heart pathology (Dambrova et al., 2021; Park et al., 2016), RET after succinate pre-accumulation was implicated in cold activation of thermogenesis by UCP1 function in brown adipose tissue (BAT) (Mills et al., 2018a). Succinate is sequestered by BAT because of cold and its intake-enhanced superoxide formation implicated in UCP1 functional activation. Even sole succinate administration in mice (but not in UCP1-KO [knockout] mice) initiated thermogenesis independently of β-adrenergic stimulation.

#### 4. Superoxide formation at hypoxia

Oxygen sensing involves a central mechanism by the action of prolyl hydroxylase domain/2OG-dependent-prolyl hydroxylases (PHDs/EGLN) (Appelhoff et al., 2004; Fuhrmann and Brune, 2017; Lu et al., 2005), providing a ferrous iron- (Fe^II^-) plus O_2_-dependent plus 2-oxoglutarate-dependent hydroxylation of hypoxia-inducible factor (HIF)-1α/HIF-2α, to mark them for constant degradation. Either the oxygen decrease, or lowering of PHD co-factors or H_2_O_2_/superoxide increase, oxidizing Fe^II^ to Fe^III^ of PHDs thus enabling stabilization of HIF-1α/HIF-2α. As a result, the HIF system is activated, inducing over 400 genes (Brocato et al., 2014; Schödel et al., 2011; Zepeda et al., 2013) (Fig. 11).

Also, factor inhibiting HIF (FIH) hydroxylating HIFα at different sites is affected by ROS H_2_O_2_/superoxide. Recently, also oxidation of reactive cysteines in PHD2 was recognized to initiate HIF-response (Briggs et al., 2016), probably due to the redox-induced formation of inactive PHD homodimers crosslinked by disulfide bridges (Chowdhury et al., 2011; Lee et al., 2016).

Despite PHDs being able to sense oxygen independently of mitochondria, mitochondrial metabolism and redox signaling represent an additional key player. PHDs are inhibited at normoxia also by the lack of fumarate, succinate, malate, isocitrate, and lactate (Hewitson et al., 2007; Koivunen et al., 2007; Plecitá-Hlavatá et al., 2017). Mitochondrial redox signaling due to reestablishing superoxide formation on restoration of Δ*Ψ*_m_ also evoked hypoxic HIF-1α stabilization in cells with deleted mtDNA polymerase having abolished respiration (and Krebs cycle turnover) (Martinez-Reyes et al., 2016).

On hypoxia onset, a hypoxic H_2_O_2_/superoxide burst was observed (Bell et al., 2007), but delayed by several hours (Nguyen et al., 2013; Plecitá-Hlavatá et al., 2015) (Fig. 11). In contrast, a hypoxic H_2_O_2_/superoxide burst occurred immediately in a minute in endothelial, HeLa, and HK2 cells (Hernansanz-Agustin et al., 2017). The Complex III site III_Qo_ was ascribed to form excessive superoxide at hypoxia, as inferred from the effects of ablation of Complex III subunits (Bell et al., 2007; Chandel et al., 2000; Chandel et al., 1998; Comito et al., 2011; Guzy et al., 2005; Sabharwal et al., 2013; Schroedl et al., 2002; Waypa et al., 2010), and such redox signal was observed even during normoxic HIF activation (Patten et al., 2010).

For example, the ablation of Rieske ISP stabilized HIF (Brunelle et al., 2005) and, in turn, S3QELs prevented the HIF response (Orr et al., 2015). Peroxiredoxin-5 overexpression in IMS exhibited attenuation of hypoxic redox signaling (Sabharwal et al., 2013), which was also indicated by IMS/ICS-addressed redox-sensitive GFP (Waypa et al., 2010). Speculations were made on why it takes several hours for the maximum HIF-1α stabilization to occur (Nguyen et al., 2013; Plecitá-Hlavatá et al., 2015), assuming a certain ICS redox buffer to be overcome during the several-hr period.

Interestingly, on acute hypoxia, Na^+^ import into the matrix is promoted *via* the Ca^2+^/Na^+^ antiporter, enabling an interaction of Na^+^ with phospholipids that reduces membrane fluidity and CoQ diffusion between Complex II and Complex III, but not in supercomplexes (see below) (Hernansanz-Agustín et al., 2020). This is just an example of the general rule depicted in Figure 4.

## IV. Architecture of Mitochondrion

### A. Compartments of complex mitochondrial topology

#### 1. Specific features of cristae architecture

**FIG. 13.**
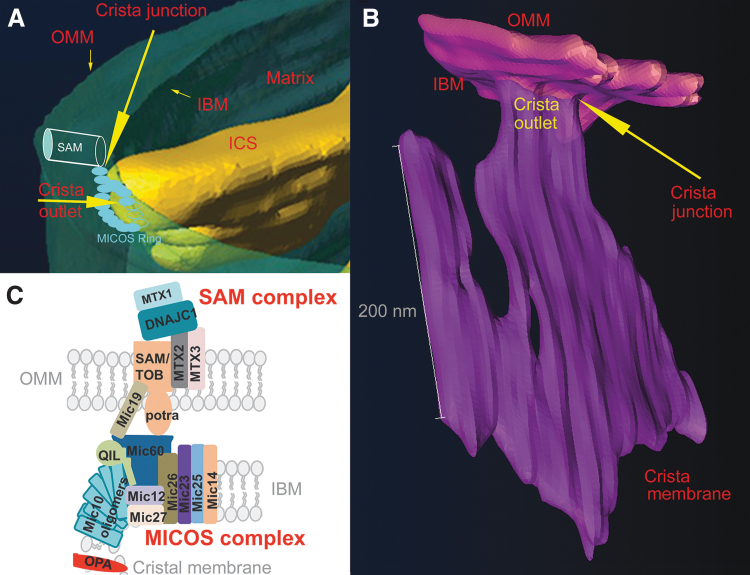
**MICOS complexes around crista outlets form crista junctions by interaction with OMM SAM complexes. (A, B)** FIB/SEM 3D images of crista junctions with indicated positions of MICOS complexes [*mild blue ellipsoids* in **(A)** which was taken as a detail of Fig. 1B] and a SAM complex for illustration. **(B)** Shows a detailed FIB/SEM image of INS-1E cell mt crista lamella (from Fig. 3A) together with a portion of OBM/OMM. The schema in **(C)** shows major subunits of MICOS and SAM complexes.

**FIG. 14.**
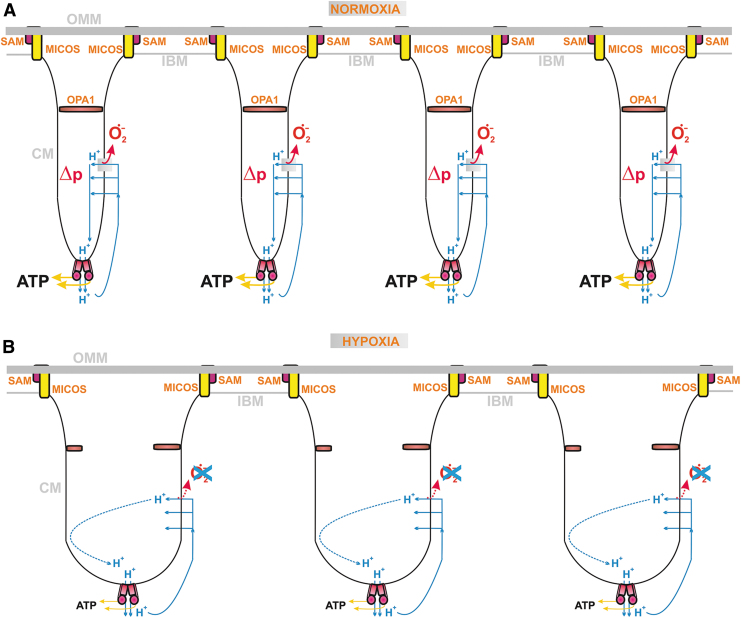
**Hypoxic relocation of MICOS complexes. (A**, **B)** Represent 2D scheme of crista outlet widening after partial MIC60 degradation at hypoxia in HepG2 cells (Plecitá-Hlavatá et al., 2016). The concomitant crista lamellae widening **(B)** is supposed to disrupt an efficient proton coupling existing at normoxia in narrow crista lamellae **(A)**.

Location of a *crista outlet* refers to an interior space, the hollow or slit-like connection of ICS with the IMS_p_ (Fig. 13A, B). Thus, the term “outlet” describes connections of spaces, whereas CJ refers to the additional connection between IBM and OMM (Fig. 13B), established by MICOS complexes (surrounding proteins of the crista outlet) attached to the SAM complexes of OMM (counterparts within OMM) (Fig. 14).

The other real physical connections of IMM and OMM are established by translocase of the outer membrane (TOM), translocase of the inner membrane (TIM), and other complexes for mt protein import (Baker et al., 2019; Iovine et al., 2021; Kondadi et al., 2020; Pernas and Scorrano, 2016). A reader might rather imagine the crista outlet as a “road junction,” emphasizing the connection of a hollow space of the crista lamellae narrowed tip with the thin intercylindrical space of peripheral IMS (Fig. 13A, B).

The *crista outlet* thus describes a hollow space, though filled and surrounded by high protein density, which is actually forming it. In any case, crista outlets (junctions) are bottlenecks for the diffusion of solutes into and out of ICS and IMS. We can speculate that if MICOS-SAM complexes form columns ordered around an ellipsoidal or slit-like shape of the outlet (Fig. 13B), there are still internal spaces between neighbor complexes for the diffusion of small molecules from ICS to IMS_p_ *via* such “sieves.”

Dynamic changes by 2D diffusion of MICOS-SAM complexes leading to emptying position around the slit subsequently allow also 2D diffusion of peripheral proteins of CMs (diffusing within the CM_ICS_ lipid bilayer leaflet) to reach IBM membrane in its leaflet facing IMS_p_. Note that peripheral membrane proteins diffusing/moving within the CM_m_ leaflet remain facing the matrix when they “jump” to the IBM matrix facing lipid leaflet.

Another term, *mitochondria-associated membranes*, was introduced for OMM proximity contacts to other cell membranes, mostly endoplasmic reticulum (ER) (Anastasia et al., 2021), but also contacts with the nuclear envelope, plasma membrane, or other cell organnelles/structures were identified (Scorrano et al., 2019). Notable are OMM connections to the cytoskeleton and ribosomes.

Proteins residing in proximity of crista outlets and forming them, such as MICOS, hypothetical OPA1 filament lattices (or as considered previously OPA1 heterotrimers), represent a barrier for aqueous diffusion between the ICS interior (lumen) and IMS_p_ (Frezza et al., 2006; Giacomello et al., 2020; Quintana-Cabrera et al., 2018b). The crista outlets, thus, also prevent leakage of cytochrome ***c*** from ICS (Cogliati et al., 2016; Pernas and Scorrano, 2016).

**FIG. 15.**
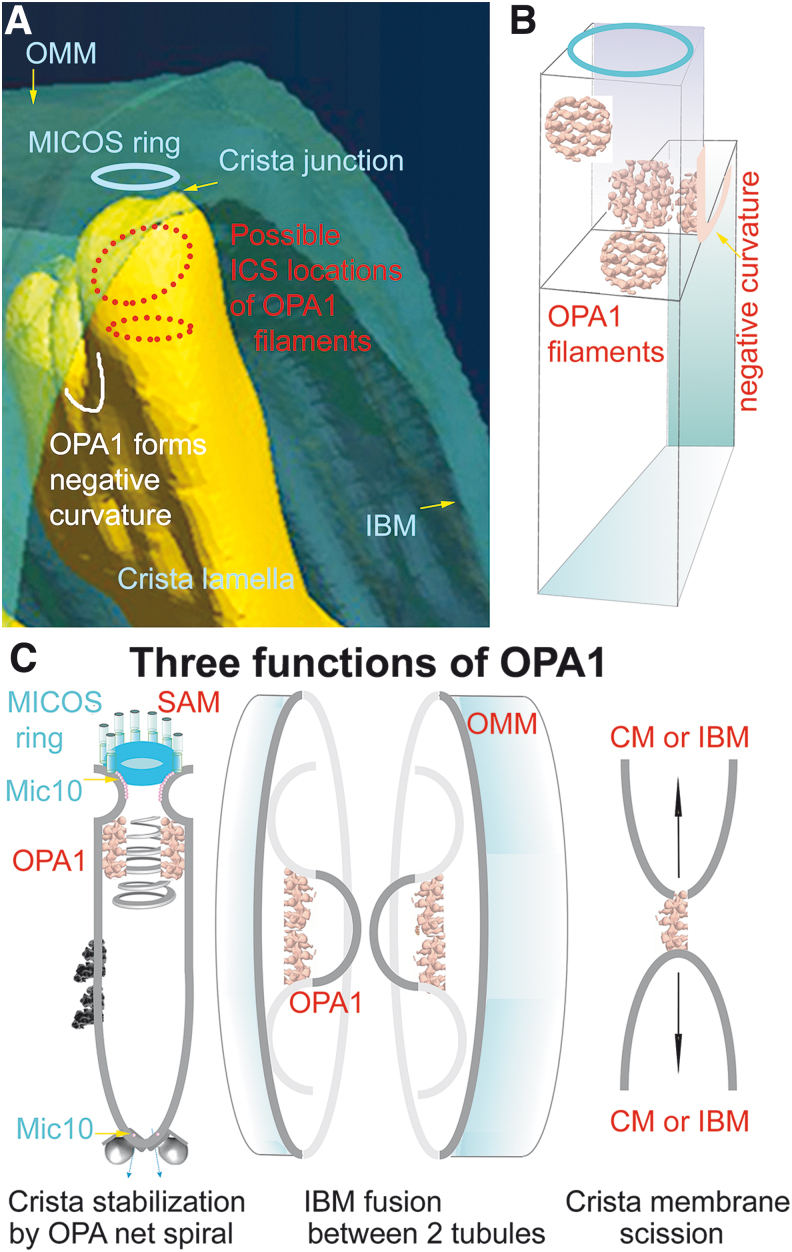
**Hypothetical locations of OPA1 in crista lamella bottlenecks and three functions of OPA1. (A, B)** Possible locations of various isoforms or their oligomers (*red dotted circles*) or, speculatively, filaments analogous to those described for fungal MGM1 **(B)** (Faelber et al., 2019). **(C)** Three functions of OPA1 summarized according to the molecular details revealed for MGM1 (Faelber et al., 2019): *Left*: crista stabilization at the bottleneck of crista lamellae; *middle*: OPA1 power stroke energized by GTP enables fusion of two proximal IMM (perhaps IBM) originating from two open ends of adjacent mitochondrial tubules; *right*: similarly the power stroke allows scission of CM or the IBM. CM, crista membrane; MGM1, mitochondrial genome maintenance 1.

The real contacts (Hessenberger et al., 2017; Jans et al., 2013; Stoldt et al., 2019) are formed by the interconnections between IBM MICOS and OMM SAM complexes (Figs. 2 and 13C), organized around the crista outlets (Bohnert et al., 2012; Pfanner et al., 2014; Plecitá-Hlavatá and Ježek, 2016; Zerbes et al., 2012). A chain of Mic10 subunits of MICOS ensures nearly 90% membrane bending at the loci where IBM becomes the crista membrane (Figs. 13B and 15A).

This is enabled by Mic10 ability to homo-oligomerize due to the GxGxGxG motif in its structure (Bohnert et al., 2015). The OMM and IBM are also interconnected by TOM-TIM complexes, protruding across IMS and ensuring protein import to the matrix, ICS, IMS, and all membrane loci. Note that various import proteins have a sorting ability for such addressing.

Immunogold EM studies identified TIM subunits and mt pro-fusion proteins in IBM, whereas, besides RC and ATP-synthase subunits, cristae membranes (CM) were found to contain proteins of Fe-S cluster biogenesis and attached subunits of mt-ribosomes (Vogel et al., 2006).

Within 400–1000 nm in diameter of mt-network tubules, cristae form lamellae typically perpendicular to the OMM cylindrical surface (Figs. 1B, 3, and 13A, B). Crista outlets are, thus, tips of irregular lamellae protrusions and it is not known yet what forms the negative CM curvature in these “bottlenecks.” One can reasonably expect that the hypothetical OPA1 filaments, prohibitin (PHB) scaffolds, or FAM92A1 (see Section IV.B.4) reside therein and support such negative curvature or even the whole shape of “bottlenecks” (Fig. 15B). Also, rather cylindrical tubular cristae were recognized, but it is not clear, whether they exist in non-pathological states (Kukat et al., 2015).

**FIG. 16.**
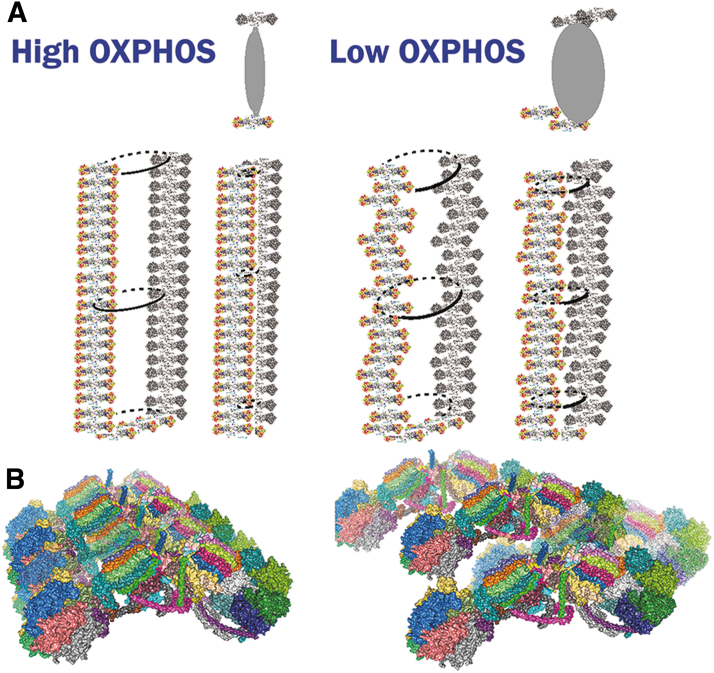
**Hypothetical mechanism of cristae narrowing by ordering/strengthening of ATP-synthase dimeric rows—and cristae widening due to weakened inter-dimeric interactions and disordered ATP-synthase dimers (see Dlasková et al., 2019). (A)** Profile of crista lamellae at high and low OXPHOS as well as ordered and disordered rows of ATP-synthase dimers, respectively. **(B)** Models of four ATP-synthase dimers in ordered and disordered rows (Dlasková et al., 2019). OXPHOS, oxidative phosphorylation.

In 3D space, cristae lamellae are not always parallel with each other (Fig. 3). The lamellae edges are formed by the array of the ATP-synthase dimers (Fig. 16), whereas other IMM proteins, including RC supercomplexes, reside in flanks of these lamellae (Fig. 4) (Davies et al., 2011). A single crista lamella is formed by the two parallel crista membranes (consisting of CM_m_ and CM_ICS_ lipid leaflets), spaced by the ICS, thus forming another sandwich, now typically perpendicular to that one formed by OMM/IBM (Pernas and Scorrano, 2016; Plecitá-Hlavatá and Ježek, 2016).

The ICS lumen width was previously reported to be 25–30 nm (Quintana-Cabrera et al., 2018a). Under optimum conditions, two CMs, each ∼6–8 nm thick, form a rather thin (10–30 nm) lamella with other two dimensions of ∼100 and 300–900 nm (Figs. 1B, 3, and 13B). Thus, any cristae lamela having a high-aspect ratio is extended nearly toward the opposite wall of the IBM/OMM cylinder. The surface area of IMM (IBM plus CM) is more than 10-fold that of the OMM in cells with high OXPHOS demand, such as cardiomyocytes (Mannella, 2020).

Traditionally, TEM visualizes random sections through the IBM/OBM cylinder (Fig. 1C), hence the shortest thin dimension of crista lamellae is visualized as a comb of typical cristae (Zick et al., 2009). That is why, 2D TEM-imaged mitochondrial cristae, represent long protrusions (Kukat et al., 2015; Sun et al., 2007) with rather sharp edges at metabolit-rich conditions (see below). We recall again that this canonical IMM morphology determines three important compartments: At first, the *matrix* has a structure resembling “an infinite octopus,” since its topology is reciprocal to cristae.

There are crista-free spaces within the length of the tubule, typically occupied with mt-nucleoids, harboring densely packed mtDNA by TFAM and containing other proteins of mtDNA maintenance, replication, and transcription machinery (Chapman et al., 2020; Ježek and Dlasková, 2019).

Second, the ICS comprised the (intra)cristal membrane (CM_m_ and CM_ICS_ phospholipid leaflets) along the crista. ICS is the major site of chemiosmotic protonic coupling of the mitochondrion (Jezek et al., 2014; Plecitá-Hlavatá and Ježek, 2016). The RC proton pumping is directed to the ICS, from where the subsequent H^+^-backflux *via* the ***c***-ring rotor of ATP-synthase provides the ultimate energy for the ATP synthesis (Rieger et al., 2021; Rieger et al., 2014) (Fig. 4).

ICS serves also as an important source of redox signaling originating from the RC Complex III site III_Qo_, either during initiation of hypoxic transcriptional reprograming by HIF system (McElroy and Chandel, 2017) or in lymphocytes (Weinberg et al., 2019) and other immune cells inducing inflammation.

Third, the IMS_p_ is a middle part of the OMM/IBM sandwich. The IMS_p_ is supposed to be established as a highly oxidative milieu where disulfide (S-S) bonds are stabilized in certain proteins. Disulfide stabilization is provided namely by the mitochondrial intermembrane space import and assembly protein 40 (MIA40)-ALR system, therein (Jezek and Plecita-Hlavata, 2009).

#### 2. Changes in cristae morphology on fusion/fission of mt network

We can predict that a different cristae morphology should exist in fragments from the continuous mt-network. Resulting spheroids could possess a size of up to ∼2 μm, since sphere fragments of 2 μm in diameter have equal OMM surface as the 10 μm long 400 nm diameter mt-tubule (Tauber et al., 2013). However, the exact characteristics of cristae morphology specific to these fragments are yet to be determined.

When “mitochondria” appear in TEM images as objects exceeding the diameter of normal mt-tubules (at least one of two dimensions in ellipsoid sections should be smaller than the OMM diameter), these particular TEM sections represent just the sections of the spheroids fragmented from the mt-network (Fig. 1C) (Dlasková et al., 2019). We still need to investigate their 3D topology, including the observed clustering of mt-nucleoids in them. The clustering of nucleoids of mtDNA and sphere character of space does not allow at least part of crista lamellae to be parallel with each other. Rather they orient perpendicularly to the spherical surface.

### B. Cristae-shaping proteins

#### 1. Arrays of ATP-synthase dimers

The ATP-synthase consists of dimers, which are organized into rows (arrays) along the sharp rims of cristae (Figs. 4 and 16) and naturally determine the cristae lamellar morphology (Fig. 3) (Davies et al., 2012; Davies et al., 2011; Dlasková et al., 2019; Dudkina et al., 2010; Gu et al., 2019; Guo et al., 2017). Cryo-EM tomography showed zig-zag localization, lateral bending, perpendicular curvature, and local non-uniformity of rows of dimers of ATP-synthase (Blum et al., 2019; Daum et al., 2013; Davies et al., 2011), reflecting perhaps distinct local oligomerization following the non-uniform crista edges (Spikes et al., 2021).

Such flexibility is enabled by variation of superpositions of the two wedges between the monomers, withstanding rotatory motion of F_1_-moiety as well as translations. We hypothesized that the order state and/or stiffness of ATP-synthase rows determines the sharpness of the crista lamellae edges (Fig. 16) (Dlasková et al., 2019).

When the ATP-synthase dimerization subunits ***e*** and ***g*** were deleted in yeast, IMM invaginations were absent and IMM was inflated into spheroids (Davies et al., 2012; Paumard et al., 2002). The IMM bending itself is ensured by numerous structural features of the membrane F_O_-moiety of the ATP-synthase within dimers (Blum et al., 2019). It was previously suggested that the ATP-synthase subunits 6.8PL (Spikes et al., 2020) and DAPIT (He et al., 2018) and the inhibitory factor IF1 (Cabezón et al., 2000) are indispensable for the dimerization; however, this was later questioned (Spikes et al., 2021).

Six sites were identified to bind together two dimers within a tetramer (Gu et al., 2019). Among them, sites *1* and *6* are formed by IF1 dimeric bridges, lifted above the membrane. Site *2* is given by interactions between subunits ***b*** and ***k***; site *4* is defined by interactions between subunits ***e*** and ***g***; and site *5* is exclusively given by mutual interactions between subunits ***e***. Ablation of either one of human subunits ***e***, ***f***, or ***g*** led to the lack of subunits ATP6/***a*** and ATP8/A6L, that is, those encoded by mtDNA. Sole ***e*** subunit ablation led also to missing subunit DAPIT (He et al., 2018).

Moreover, Mic10 (see Section IV.B.2) was reported to crosslink subunits ***e*** of the neighboring dimers within a row at the crista rim (Rampelt et al., 2017). It has to be determined whether these sites and Mic10-crosslinks stabilize crista rims *in vivo*. Also, Mic27 was found to interact with the ATP-synthase (Eydt et al., 2017). Note that the disrupted ATP-synthase dimerization due to the mutant DAPIT subunit drastically reduced cristae in fibroblasts (Siegmund et al., 2018).

We hypothesized that when the order is weakened for the ATP-synthase dimers along the rim of the crista (lamella edges), the sharp crista edge is transformed into a flatter edge or rim (Dlasková et al., 2019). Consequently, a flatter rim allows more inflated ICS and a higher distance of parallel flanks of cristae lamellae at least in their centers (Fig. 16). This would be apparent in TEM sections as widened cristae. Note also that tubular cristae, that is, those without edges, would hardly possess ordered ATP-synthase dimeric arrays.

As FIB/SEM images demonstrate, indeed the widened cristae lamellae exist, which are more inflated and without sharp edges. In the other words, the sharp edge of cristae lamela exists when longitudinally ordered ATP-synthase dimers are tightly packed along the lamela edge or tip. Such a nearly one-dimensional crystal structure allows only bended cristal membrane. Its negative curvature is also determined by prevailing CL and phosphatidyletanolamine.

In contrast, when the longitudinal arrangement of ATP-synthase is loosened so that individual dimers could even slightly move transversally to the crista lamela edge, then the membrane under these dimers can no longer stay sharp which consequently allows the widening of the lamellae (Fig. 16).

It should be further studied, whether a recruitment exists for “glue” proteins, such as Mic10, to intercalate between ATP-synthase dimers, and whether this is the only force and action required for strengthening these dimeric arrays. Alternatively, it should be determined, whether certain ion efflux from ICS (cation plus anion followed by water) accompanies or even initiates the mechanistic force, to shrink the ICS. Of course, both the above processes can participate.

Vice versa, the hypothetical loss of “glue” proteins, for example due to their post-translational modifications, may weaken the dimeric rows. Alternatively, signaling can initiate some ion uptake into ICS and the concomitant water influx then inflates the crista. Thus, de-sharpening of crista lamela edges and hypothetical water influx into the ICS may transfer the “thin cristae” into bulky ones. The third alternative process would involve a simple fusion of two adjacent cristae lamellae into a single bulky lamella. The problem with this hypothesis lies in the way, how the double adjacent membranes would be rearranged or merged into the single one.

#### 2. MICOS complexes

Surrounding the crista outlet pores or slits, actual connections between IMM and OMM are substantiated by the IMM complexes of mitochondrial contact site and cristae organizing system (MICOS) (Bohnert et al., 2012; Harner et al., 2014; Harner et al., 2011; Jans et al., 2013; Ott et al., 2015; Pfanner et al., 2014; Yang et al., 2015; Zerbes et al., 2012), which is attached to the OMM SAM/TOB complex (Höhr et al., 2015; Körner et al., 2012) (Fig. 13).

Mitofilin/Mic60, as a major subunit of MICOS, interacts with the POTRA domain of Samm50 (Höhr et al., 2015; Ott et al., 2015) and joins directly the MICOS complex with the SAM complex providing real CJs (Fig. 13C). After ablation of mitofilin/Mic60, Mic10, and partly Mic27, ICS detached from OMM forms isolated inner compartments (cristae vesicles or lamellae parallel to longitudinal axis of mt tubules) within the matrix space, which became adjacent to OMM, whereas Mic19 silencing led also to cristae branching (Harner et al., 2011).

Mic60 phosphorylation by Ser/Thr kinase PTEN-induced putative kinase-1 (PINK1) was found to stabilize Mic60 oligomerization in *Drosophilla* (Tsai et al., 2018). Superresolution imaging has shown that Mic60 is arranged in helical arrays along a mt tubule (Stoldt et al., 2019) and so CJs (outlets) should be as well.

Mic10 oligomers (Fig. 13C) are essential for cristae formation by organizing phospholipids so as to form hairpins enabling the 90 degrees bending of IBM around the hollow crista outlet (Barbot et al., 2015; Bohnert et al., 2015). Thus, Mic60 and Mic10 are key subunits of the MICOS complex for forming membrane curvature, as also inferred from the ability of Mic60 to reshape liposomes into thin tubes (Hessenberger et al., 2017) and from the *de novo* formation of CJs by controlled Mic60 expression in HeLa cells with ablated Mic60 or Mic10 expression in Mic10-deficient cells (Stephan et al., 2020). Mic13 seems to be an assembly factor for MICOS, connecting *via* conserved GxxxG motif two adjacent MICOS complexes (Urbach et al., 2021).

#### 3. Optic atrophy 1

OPA1 is a GTPase, the various forms of which are involved in both mt-network fusion as well as in cristae formation (Giacomello et al., 2020; Pernas and Scorrano, 2016). Alternative splicing provides eight OPA1 isoforms expressed in distinct patterns in different tissues. The long-form splice variant L-OPA1 is attached *via* its transmembrane loops to IMM. Proteases cleave L-OPA1 into short forms S-OPA1. These are namely OMA1, inhibiting mt-network fusion, and constitutively active YME1L, required for fusion (Anand et al., 2014).

Proteolysis by another protease, PARL, contributes to a soluble S-OPA1 pool in ICS, from where the hypothetical OPA1 trimers would be formed to guard the crista outlets (Frezza et al., 2006). OPA1 is also post-translationally modified, so that acetylation (and SIRT3-mediated deacetylation) (Samant et al., 2014), nitrosylation (Bossy et al., 2010), and O-linked N-acetylglucosamine glycosylation (Makino et al., 2011) were found, namely under stress conditions.

A balance between soluble S-OPA1 and L-OPA1 controls cristae junctions, as suggested, for example, by rescue from apoptosis after OPA1 expression (Frezza et al., 2006). In turn, OPA1 deficiency leads to inflated cristae and widening of crista outlets (Frezza et al., 2006; Olichon et al., 2003), loss of RC supercomplexes, and increased crista width (Cogliati et al., 2013). Independently of its GTP-ase function, S-OPA1 transformed liposomes into tubules (Zhang et al., 2020a). Moreover, OPA1 interacts with Mic60 of MICOS (Barrera et al., 2016; Glytsou et al., 2016) as well as with Mic19 (Darshi et al., 2011).

OPA1 ortholog mitochondrial genome maintenance 1 (MGM1) from a thermophilic fungus was crystallized and it was demonstrated that tetrameric MGM1 forms polymeric helical coating lattice on the outside of lipid tubes (prepared with the aid of optical tweezers) and also possesses the ability to constrict their diameters (Faelber et al., 2019). Moreover, in the apo form, Mgm1 tetramers decorated the inner surface of lipid tubes, thus forming a lattice inside and again with the ability to constrict or expand such lipid tubes.

Then, a GTP-driven conformation change within a dimer shortens the dimer dimension. Termed, a *dynamin-like power stroke*, such conformation change enables constriction of membrane tubes. Consequently, the G-domain dimerization may link neighboring MGM1 lattice filaments when a power stroke happens, enabling two membranes to be pushed toward each other if each membrane harbors such a filament. Note also, that the GTPase activity of OPA1 is required to sustain cristae morphology (Frezza et al., 2006) (Fig. 15).

Analyzing all mechanistic behavior of MGM1 filaments, Faelber et al. (2019) suggested that it explains all three MGM1/OPA1 molecular functions (Fig. 15C): (1) stabilization of mitochondrial cristae, as originally suggested; (2) IBM fusion inside of two adjacent open OMM tubules when the two MGM1/OPA1 lattices residing on proximal membranes adhere externally pushing these two adjacent membranes to join after the power stroke; (3) IMM (CM) scission when the MGM1/OPA1 lattice pulls apart two originally adjacent membranes (Meeusen et al., 2006). For the cristae stabilization, the MGM1/OPA1 lattice joins zig-zag the opposite parallel CMs within the crista lamella in the proximity of crista outlets (junctions), where MGM1/OPA1 may also interact with MICOS complexes.

OPA1 sustains mitochondrial ATP levels and homeostasis during starvation (Gomes et al., 2011), limited respiratory substrate availability (Patten et al., 2014), or compromised electron transfer (Quintana-Cabrera et al., 2018b), whereas improved bioenergetics and electron transfer by RC may prevent excessive superoxide generation (Lopez-Fabuel et al., 2016). OPA1 also adjusts the mitochondrial uptake of calcium *via* the ER mitochondrial contacts (Cartes-Saavedra et al., 2021).

We can conclude that the intact OPA1 function and regulation, which contributes to cristae narrowing, enables stability of cristae junctions, which further allows stability of RC supercomplexes and optimum OXPHOS (Civiletto et al., 2015; Cogliati et al., 2013; Varanita et al., 2015). This stabilization role is inferred from experiments when OPA1 or its correct regulation is deficient, which leads to impairment of the ATP-synthase dimeric arrays (Amutha et al., 2004; Patten et al., 2014; Quintana-Cabrera et al., 2018b).

#### 4. Other cristae-shaping proteins

High-molecular-weight scaffolding proteins PHBs PHB1 and PHB2 form hetero-oligomeric rings of ∼20 × 27 nm in IMM (Tatsuta et al., 2005), providing a scaffold for various protein complexes together with lipids enabling negative membrane curvatures, such as PE and CL (Osman et al., 2009; Tatsuta and Langer, 2017b).

Since cristae outlet diameter is similar to PHB oligomeric ring sizes (cf. Fig. 13B), PHBs likely provide a stabilizing structural basis for cristae with a perfect fit. When PHB decrease was induced by a deficiency of sphingosine-1-phosphate (Hong et al., 2018), also a decreased mtDNA copy number was observed, which caused deficiency of mtDNA encoded subunits for RC and ATP-synthase (Supale et al., 2013). A scaffold breakdown affects also OPA1, since PHB2 depletion led to L-OPA1 loss and perturbed cristae morphology so that cristae formed vesicles (Merkwirth et al., 2008).

The changes were partly rescued by the addition of L-OPA1. PHB2-KO mice in the forebrain together with OMA1 deletion maintained L-OPA1 but did not rescue cristae morphology or RC supercomplexes assembly and activity, despite increasing lifespan and rescue of mtDNA content and neuronal death (Korwitz et al., 2016). Hence, PHB2 influences cristae remodeling *via* its lipid-scaffolding function and not directly *via* OPA1.

PHB benefits to correct crista morphology were observed even in human sperm, despite the lowest mtDNA copy number of all cell types present. A lowered energy caused poor sperm motility when PHB expression was low, associated with oxidative stress and lipid peroxidation due to excessive superoxide produced at CI (Chai et al., 2017).

CJs were also lost on depletion of FAM92A1 protein. FAM92A1 was found to locate to the CM_m_ lipid leaflet and to contain a Bin/amphiphysin/Rvs (BAR) domain, typical for endophilin and amphiphysin, proteins that anchor to plasma membrane clathrin triskelion units at the neck of endocytic vesicles. It was therefore suggested that FAM92A1 binds negatively charged lipids such as CL and phosphatidylinositol 4,5-bisphosphate, so as to stabilize the negative cristae membrane curvature and maintain cristae ultrastructure (Wang et al., 2019b). Purified FAM92A1 induced tubulation of unilamellar vesicles. FAM92A1 depletion resulted in formation of crista vesicles and/or lamellae sheets separated from the IBM. Simultaneously, the altered cristae caused lower oxygen consumption and RC activity (Wang et al., 2019b).

Depletion of mitochondrial calcium uptake 1 (MICU1) protein resulted in widened CJs, hence crista width was suggested to be controlled by MICU1 hexamers (Gottschalk et al., 2019). Increasing Ca^2+^ around MICU1/MICU2 otherwise results in control of mitochondrial calcium uniporter (MCU), thus affecting mt calcium homeostasis (De Stefani et al., 2016). MICU1 depletion also increased the release of cytochrome ***c*** from ICS, and diminished Δ*Ψ*_m_ (Gottschalk et al., 2019). Note also that overexpression of leucine zipper and EF-hand containing transmembrane protein 1 (LETM1) together with carboxyl-terminal modulator protein in hepatocellular carcinoma led to a loss of cristae and antitumor effects in H-ras12V mice (Shin et al., 2013).

Among the BCL-2 family proteins, full-length BID, BCL-X (McNally et al., 2013), and MCL-1 (Perciavalle et al., 2012) aid in the maintenance of cristae independently of apoptotic initiation and caspase-8 cleavage. Thus, full-length BID localizes to mitochondria even without apoptotic stimulus. When *Bid* is ablated in mice, left ventricular cardiomyocytes possess almost no cristae or have abnormal cristae due to decreased RC complexes and ATP-synthase (Salisbury-Ruf et al., 2018). In wt cardiomyocytes. The intact BID, but not mutated BID^M148T^, associates with MCL-1 in the matrix, hypothetically strengthening cristae organization, whereas cBID interaction with BAX opens cristae to release cytochrome ***c***.

### C. Morphology and dynamics of cristae

#### 1. Changes in cristae morphology reflect metabolic states

A common impulse for physiological changes in cristae morphology is, for example, a sudden increase in respiration substrate (Dlasková et al., 2019; Dlasková et al., 2018; Plecitá-Hlavatá et al., 2016). The common outcome observed in reported cases was that at higher substrate (and respiration) levels, cristae became narrower in TEM images and exhibited 3D morphology with thinner parallel lamellae in FIB/SEM (Fig. 3).

For example, a higher substrate and respiration is represented in pancreatic β cells by the switch from low glucose concentration at the resting state, when insulin is not secreted, to high glucose, that is, to the active state, when glucose-stimulated insulin secretion (GSIS) proceeds (Dlasková et al., 2018).

The cristae morphology in pancreatic β cells at high glucose (on GSIS) is apparent in TEM as typically ordered narrow “textbook cristae.” This jargon, expressing the apparent width of stained crista in TEM sections, actually describes the minimum shortest dimension of cristae lamellae (Fig. 1C). Despite similar heavy metal staining, 3D images of FIB/SEM provide more detailed topology of cristae than TEM. Rather sharp edges of cristae lamellae were found at high glucose in both INS-1E cells in culture and β cells of isolated pancreatic islets (Fig. 3).

The lamellae were nearly parallel with the short-longitudinal length segments of mt tubules. A few contacts with IBM could be identified, being much narrower than the overall crista lamela (Fig. 13B). At low glucose concentration, that is, with less intensive OXPHOS, where no insulin is secreted, both INS-1E cells and pancreatic islet β cells exhibited widened cristae in TEM sections (Dlasková et al., 2019).

**FIG. 17.**
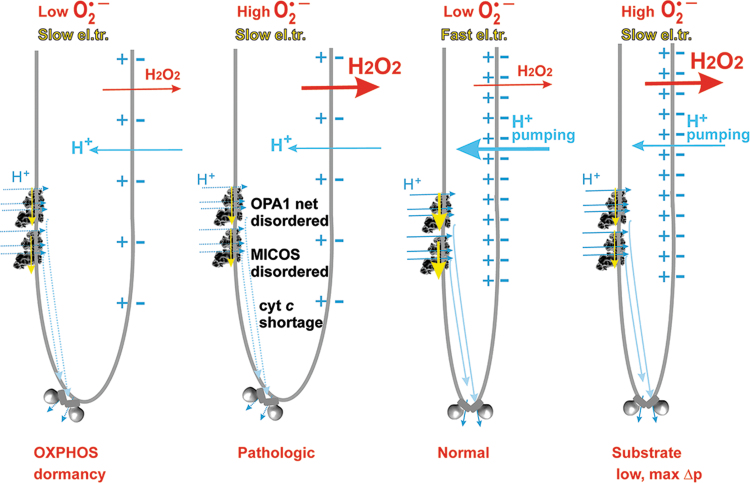
**Superoxide formation *versus* electron transfer rate—all four possible combinations of low/high superoxide formation rate and low/high electron transfer rate are depicted and illustrated by the existing examples.** OXPHOS dormancy, existing for example, in certain cancer cells or hypoxia-adapted cells, exhibits minimum respiration and hence low electron transfer, but also minimum substrate pressure not inducing any significant superoxide formation. Pathological conditions typically exhibit defects leading to high superoxide formation and low respiration (electron transfer). Normal conditions in contrast exhibit unretarded but fast electron transport due to optimum, presumably high, OXPHOS. The unretarded character allows relatively low superoxide formation at faster respiration. At low substrate but unobstructed electron transfer and proportionally lowered OXPHOS, still sufficient to create a high Δ*p*, a higher superoxide formation exists due to feedback inhibition of proton pumping (respiratory control).

In parallel studies, we demonstrated that despite faster respiration, β cells diminish mitochondrial superoxide formation on GSIS, whereas rather a high superoxide release into the matrix was observed at low glucose, associated with lower respiration (Plecitá-Hlavatá et al., 2020). Thus, one can correlate higher respiration, higher ATP synthesis, lower mt superoxide formation, and sharper and more ordered cristae morphology on one hand; and low(er) respiration, low ATP synthesis, higher mt superoxide formation, and unordered bulky cristae existence, on the other hand (Fig. 17, panel “normal”).

This is similar to hypoxic adaptation (Dlasková et al., 2019; Plecitá-Hlavatá et al., 2016). In other words, narrower cristae correspond to a more intensive OXPHOS and slower mitochondrial superoxide formation, whereas inflated cristae exist at states of low OXPHOS and higher superoxide formation.

One cannot yet make a general statement on cristae morphology relations to OXPHOS and mt superoxide formation until more examples are characterized. Nevertheless, the above relationships are fulfilled also in hypoxia-adapted hepatocellular carcinoma HepG2 cells (Dlasková et al., 2019; Plecitá-Hlavatá et al., 2016) Cristae widening occurred after the hypoxic adaptation of HepG2 cells when HIF transcriptome reprogramming sets OXPHOS rather dormant and low-rate Krebs cycle turnover establishes a lower substrate pressure (NADH/NAD^+^ ratio).

However, a sudden addition of cell-permeant substrate to such hypoxia-adapted cells instantly restored high NADH/NAD^+^, and higher respiration and resulted in cristae narrowing (Dlasková et al., 2019). We summarize these changes in Figure 17.

The cristae morphology changes occurred also due to the Δ*Ψ*_m_-sensitivity of OPA1 cleavage (Pernas and Scorrano, 2016) or on PINK1-mediated protein degradation, in conjunction with Parkin and the ubiquitin-proteasome system (Fallaize et al., 2015). Cristae widening has been also linked to Mic60 and OPA1 downregulation during placental trophoblast differentiation into syncytiotrophoblasts, which maintains progesterone production (Wasilewski et al., 2012).

#### 2. Dynamics of cristae morphology and possible fusion/fission of cristae

A view that also cristae morphology changes are frequently varied within rather short time intervals and may change at a rather fast pace of seconds was suggested by recent experiments (Huang et al., 2018; Kondadi et al., 2020; Stephan et al., 2019; Wang et al., 2019a). The advent of superresolution microscopy/nanoscopy has brought modest (Fiolka et al., 2012; Shim et al., 2012) and convincing images of mitochondrial cristae (Huang et al., 2018; Jans et al., 2013; Kondadi et al., 2020; Schmidt et al., 2009; Stephan et al., 2019; Stoldt et al., 2019; Wang et al., 2019a), actually resembling lamellae identified by FIB/SEM.

Recently, fluorescence nanoscopy techniques were applied to living cells, using PHB2 fused with mScarlet dye or employing SNAP-tags stained with STED-active fluorophores after the expression of tagged proteins and revealed remarkable results of cristae dynamics (Kondadi et al., 2020). Cristae junction dynamics was followed using subunits of MICOS complex as markers visualizing dynamics, such as Mic10 and Mic60 apparent as punctate structures in 2D projections (Jans et al., 2013; Kondadi et al., 2020; Stoldt et al., 2019).

CJs moved toward each other or apart and this mobility turned out to span even 50 nm distance in a time scale of seconds (Kondadi et al., 2020). However, please note that approximately such a distance exists between the neighbor cristae lamellae (Fig. 3C). The importance of MICOS for cristae architecture and dynamics was deduced from resulting changes when Mic60 or other MICOS subunits were ablated, for example, Mic13 ablation decreased the number of merging/splitting cristae lamellae.

Cristae membranes were also visualized by SNAP-tagged subunit ***e*** of membrane F_O_-moiety of ATP-synthase or SNAP-tagged COX8A subunit of Complex III, and stained with STED fluorophores (Kondadi et al., 2020). Such nanoscopy indicated the lamellae movement not only toward each other but also resembling the letter X or Y in side views. The reader has to relate these finding to findings of cristae reticulum observed on apoptosis (Mannella, 2008). Corresponding, but static, similar 3D images were previously described for neuronal mitochondria (Perkins et al., 1997) or apoptotic mitochondria (Sun et al., 2007).

When using a very photostable dye MitoPB yellow, the merging of individual crista was confirmed (Wang et al., 2019a). Using polyethylene glycol cell fusion and cells with crista stained with different dyes, subsequent dye mixing suggested cristae fusion (Kondadi et al., 2020). These authors alternatively employed a photoactivable GFP fused to ATP-synthase subunit ***e***. When locally activated, even neighboring cristae were fluorescent.

Hence, excluding fast 2D-diffusion within IMM, this result could serve to support cristae fusion. Also, experiments of Busch and colleagues directly tracking trajectories of ATP-synthase could support such a conclusion (Appelhans and Busch, 2017; Busch, 2020; Weissert et al., 2021; Wilkens et al., 2013). Nevertheless, the dynamic cristae fusion and fission still can be considered hypothetical. Apoptotic changes could proceed as a single irreversible event. However, the autonomy of individual cristae is supported by findings of individual bioenergetics properties of single cristae (Wilkens et al., 2013; Wolf et al., 2019).

## V. Molecular Physiology of Cristae

### A. Morphology changes of mitochondrial cristae

#### 1. RC and protonic coupling relations to size and shape of crista lamellae

We can assume a single crista as protruding 300 nm into the 400 nm diameter OMM tubule and having a second dimension of 200 nm and thickness of 20 nm (distance between the parallel crista membranes). Then, we can predict that a 300 × 200 nm flank of the lamella may accommodate up to 200 (10 times 20) Complex I structures, when assuming 10 nm spacing between them in both dimensions (Figs. 3 and 4). In other words, the 300 nm protruding crista could contain up to 400 supercomplex structures, 200 in each of its flank.

However, the 800 nm long edge of such lamella may accommodate approximately only 67 dimers of ATP-synthase at its edge, if assuming their 12 nm thickness. Considering 1:1 stoichiometry of Complex I to ATP synthase F1-moiety, instead of 400 supercomplexes, there would be only 134 of them, 67 in each lamella flank. These theoretical considerations are supported by experiments (Fig. 4), showing 30 ATP synthase dimers on the edge and 26 (twice 13) supercomplexes on both flanks on a crista lamella of a similar size (Nesterov et al., 2021).

However, migration of Q between complexes proceeds in limited dimensions around the supercomplexes and if the crista is not completely aborted, it should stay so when the originally sharper ATP-synthase-ensured edges become flatter. This theoretically happens on the observed physiological cristae widening. We predict that unless cytochrome ***c*** and CoQ are depleted, electron transfer and respiration should be preserved.

Note that only the QH_2_ diffusion from the Complex II, ETFQOR, and other Q-linked CM-residing DH should proceed by longer distances in bulky crista (Fig. 4). Actually, this principle would hypothetically provide a direct relationship between the CII superoxide formation and wider (bulkier) or disordered cristae morphology. Longer QH_2_ diffusion means delayed diffusion with a higher chance to form superoxide in the relevant sites.

Also, the protonic coupling should be weaker under “flat bulky cristae” or vesicular cristae conditions, when compared with sharper lamelar cristae (Fig. 4 bottom and Fig. 14A, B). Protons should diffuse within the sharpest crista up to 5 nm to travel a distance between the ATP-dimeric arrays and a neighbor array of supercomplexes (Nesterov et al., 2021). A bulkier crista with flatter edges would increase these distances for H^+^ diffusion and protonic coupling. Of course, this should correlate with lower intensity OXPHOS and even dormant OXPHOS, plus also with higher superoxide formation.

#### 2. Cristae morphology in relation to potassium homeostasis

The mitochondrial K^+^ transport cycle has been predicted (Mitchell and Moyle, 1967), which provides a key role in maintaining mitochondrial volume homeostasis. This cycle should prevent the excess matrix swelling and thus maintain the structural integrity of the organelle, as well as prevent the excess matrix contraction when K^+^ influx declines due to IMM depolarization (Checchetto et al., 2021; Garlid and Paucek, 2003).

The mitochondrial K^+^ transport cycle consists of influx and efflux pathways for K^+^, H^+^, and anions and is highly regulated to respond to changing conditions *via* signals from both the mitochondrion and the cytosol (Garlid and Paucek, 2003). Electrogenic proton ejection (pumping) by RC generates protonmotive force Δ*p*, which drives K^+^ influx from ICS into the matrix by diffusion (termed “K^+^ leak”) and also *via* distinct mitochondrial K^+^ channels (Kravenska et al., 2021). Such a continuous diffusive K^+^ influx, accompanied by the influx of anions and osmotically obligated water, threatens the integrity of the organelle.

This imbalance is countered by the electroneutral K^+^/H^+^ antiporter, whose activity is inhibited by matrix Mg^2+^ and other divalent cations (Jezek et al., 1990; Li et al., 1990). In isolated mitochondria, the activity of the K^+^/H^+^ antiporter was also stimulated by the increased matrix volume even under conditions when Mg^2+^ is depleted (Brierley et al., 1984), which led to speculations that the K^+^/H^+^ exchange may be regulated by conformational changes induced by membrane stretching (Garlid and Paucek, 2003).

The molecular identity of the K^+^/H^+^ antiporter is not known, although studies show that the IMM protein LETM1 participates in mitochondrial K^+^/H^+^ and Na^+^/H^+^ exchange in humans (Natarajan et al., 2021), and decreased LETM1 activity results in K^+^ accumulation in mitochondria in HeLa cells (Austin et al., 2017).

In addition to the K^+^ influx by diffusion, the IMM and IBM also contain a variety of K^+^ channels (Checchetto et al., 2021; Kravenska et al., 2021), although the physiological roles for most of them are still far from being understood. Concerning observed K^+^-channel phenomena (Kravenska et al., 2021), they were characterized in swollen mitoplast using patch-clamp.

The membrane area inspected under the patch-clamp pipette could be composed entirely by IBM or should exist at a more swollen state (of cut mt tubules termed mitochondria, and when devoid partly of OMM, termed mitoplasts). Inevitably, a part of this area comes from the enflipped CM. Consequently, we do not know whether any of these reported channels reside in cristae lamellae flanks or only within IBM. Annotation of those K^+^-channel phenomena fall into two categories, either being identical to the plasma membrane channels of the same function or being mitochondria-specific.

A saga of annotation for the mitochondrial ATP-sensitive K^+^-channel (mtK_ATP_) involved both above-mentioned categories during previous decades of research. However, the recent most probable annotation prefers a coiled-coil domain-containing protein 51, CCDC51, acting in synergy with a mitochondrial ABC protein, ABCB8 (Paggio et al., 2019).

The reconstituted CCDC51-ABCB8 complex exhibited 57 pS conductance and its K^+^-uniport, also affecting mitochondrial volume, was blocked by ATP, glibenclamide, and 5-hydroxydecanoate. It was also activated by diazoxide, but only when ABCB8 was present. All these aspects have long been ascribed to the mtK_ATP_ phenomenon participating in cardioprotection (Costa et al., 2006; Garlid et al., 2013).

The mtK_ATP_ was found to be regulated by a variety of biochemical and pharmacological agents (Jabůrek et al., 1998), as well as post-translational modification by protein phosphorylation (Costa and Garlid, 2008; Jabůrek et al., 2006). It should be noted that although the mtK_ATP_ is sensitive to ATP, there is no evidence that mtK_ATP_ activity *in vivo* is regulated by changes in ATP concentration. However, there is strong evidence that mtK_ATP_ is normally closed *in vivo* by physiological concentrations of ATP.

Nevertheless, mtK_ATP_ can be opened in the presence of inhibitory concentrations of ATP by pharmacological and physiological ligands (Jabůrek et al., 1998), and also by a variety of endogenous signaling pathways, including those activated by brief ischemia followed by reperfusion (Garlid et al., 2009). It has been suggested that the main role of mtK_ATP_ in maintaining mitochondrial volume homeostasis is to prevent matrix contraction when K^+^ influx declines due to membrane depolarization (Costa et al., 2006; Garlid and Paucek 2003).

For example, when Δp moderately decreases, such as during high rates of ATP synthesis, K^+^ influx by diffusion is therefore more restricted, and the transient imbalance between K^+^ influx and efflux would cause the matrix volume to shrink or contract to a lower steady-state volume. This could theoretically lead to ICS volume expansion, when OMM volume remains constant. However, opening of mtK_ATP_ by endogenous signaling pathways would create a parallel K^+^ influx pathway so that the K^+^ influx and matrix volume are maintained despite lower thermodynamics driving forces (Garlid and Paucek, 2003).

Although there is a consensus that the regulation of mitochondrial volume homeostasis is fundamental in cellular physiology, and that the mitochondrial K^+^ cycle plays a key part in maintaining the matrix volume, the particular roles and regulations of mitochondrial K^+^ transport in maintaining and rearranging the cristae morphology are still elusive and remain hypothetical as described in the next section.

#### 3. Hypothetical rearrangement of cristae morphology by the mitochondrial potassium transport cycle

**FIG. 18.**
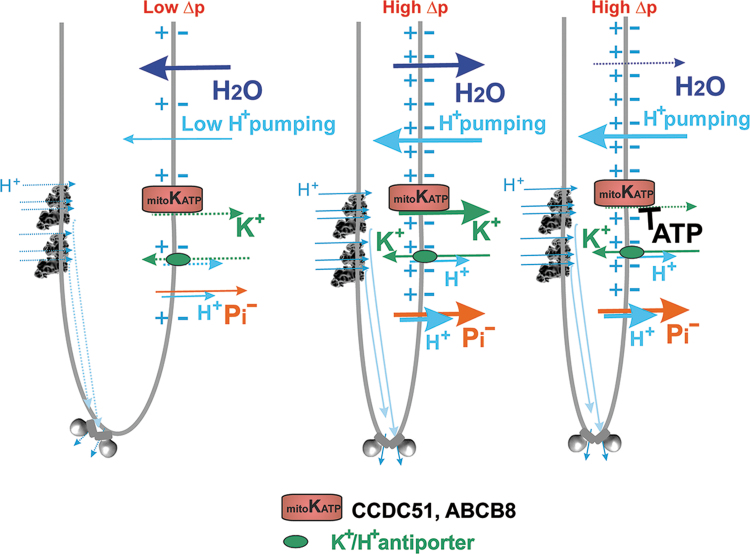
**Hypothetical participation of mitochondrial ATP-sensitive K^+^ channel in cristae narrowing.** *Left*: at low OXPHOS state with bulky crista lamellae at lower ATP levels and low proton pumping rate, mitochondrial ATP-sensitive K^+^channel (“mitoK_ATP_”) might be hypothetically open, allowing a K^+^ efflux from ICS to the matrix, which would balance existing low proton pumping. Concomitant electroneutral phosphate efflux from ICS together with cation efflux would only slightly increase matrix osmolarity, but an electroneutral K^+^ influx into ICS *via* the mitochondrial K^+^/H^+^ antiporter would compensate for osmolarity increase or might even overcome it (in conjunction with some organic anions exported from the matrix), so that rather a water intake into ICS would prevail and hence bulky crista would be preserved. *Middle*: When a sudden substrate intake occurs, then respiration and Δ*p* are instantly increased, but ATP has not yet accumulated. This would allow to keep still opened mitoK_ATP_ to mediate an additional K^+^-efflux from ICS to the matrix, causing the osmotic imbalance due to a suddenly elevated phosphate efflux from ICS. This should induce water efflux from ICS to the matrix with concomitant cristae shrinking (narrowing). Alternatively, various effectors, including phosphorylation, as described in the article text, will open mitoK_ATP_ and/or other K^+^channel. *Right*: During continuing higher OXPHOS ATP will accumulate, leading to the closure of the mitoK_ATP_ ensemble. Thus, water efflux from ICS would stop as would stop ICS shrinking. This would also prevent an infinitive shrinking of cristae lamellae.

In the above-described K^+^ transport cycle, under specific conditions various IMM K^+^-channels allow an influx of K^+^ into the matrix, whereas the IMM K^+^/H^+^-antiporter expels K^+^ to either ICS or IMS_p_ at the expense of Δ*p* (Jezek et al., 1990; Li et al., 1990) (Fig. 18). Topology of mitochondrion restricts even diffusion of ions. Situations were found, where even OMM voltage-dependent anion channel (VDAC) pore did not allow K^+^ or Ca^2+^ uptake, hence one cannot automatically assume that their cytosolic concentrations will equal to those in ICS and IMS (Kravenska et al., 2021).

The matrix concentration of K^+^, [K^+^]_m_, is reported to be 150–180 m*M*. More importantly, when K^+^ influx is concomitant to the influx of anions, for example, phosphate, and overall salt influx to the matrix exceeds that of efflux, osmolarity is imbalanced and water transport to the matrix follows. Unlike in isolated mitochondria (and in spheroids fragmented from the main mt network), we can distinguish where this salt and water uptake proceeds.

If it proceeds as a salt uptake exclusively across IBM, resulting matrix swelling can push onto cristae membranes and one could assume their rearrangement is due to mechanical forces and even possible narrowing. The lateral tension should be increased by matrix swelling that speculatively pushes crista outlets to become smaller. Also, when the water uptake proceeded exclusively across cristal membranes from ICS to the matrix, one would assume a direct cristae shrinkage.

In contrast, when matrix water diffuses *via* CM into the ICS, this would lead to cristae inflation, that is, possibly to bulkier lamellae with shorter protrusions. If simultaneously transport accross the IBM is zero, resulting ICS swelling and concomitant matrix contraction would produce wider cristae outlets (Plecitá-Hlavatá et al., 2016). The opposite situation of water efflux from ICS to the matrix at zero flux accros IBM would always cause cristae shrinkage (Fig. 18). At no flux accross IBM, matrix swelling could accompany such cristae narrowing.

However, it is not known which channels and ion carriers and at what direction act under these conditions. The general rule could be predicted that a K^+^/H^+^-antiporter, independently of its location and annotation, must translocate K^+^ to the ICS or IMS_p_ in the respiring mitochondrion, since Δ*p* drives H^+^ translocation *via* a K^+^/H^+^-antiporter in direction from the ICS (IMS_p_) to the matrix.

The stoichiometric K^+^ flux would go to the ICS (IMS_p_). At the same time, any K^+^-channel might extrude a few K^+^ ions from the ICS, due to the negatively charged CM_m_, established by RC proton pumping. However, if one would consider mtK_ATP_ to promote this, this channel should be closed by a high ATP at higher Δ*p*, so only the K^+^/H^+^-antiport and hence K^+^ uptake into the ICS or IMS_p_ would be functional, despite simultaneously narrow cristae. The above mentioned hypothetical effectors, such as phosphorylation, would cause the mtK_ATP_ opening.

Unfortunately, studies on how activation/inhibition of mtK_ATP_ affects cristae were not performed, obviously due to their complexity and difficulty. Based on the known regulations of mtK_ATP_, we can consider the mtK_ATP_-mediated K^+^-efflux from ICS to the matrix, to keep a narrow crista and predict its opening at higher ATP elevations by any of the effectors (such as GTP) or post-translational modification.

Alternatively, when at a low OXPHOS state with bulky cristae at a lower ATP level, which would still allow mtK_ATP_ opening, a sudden substrate intake occurs, then respiration and Δ*p* are instantly increased, but ATP has not yet accumulated (Fig. 18). This would allow opened mtK_ATP_ to mediate additional K^+^-efflux from ICS to the matrix, which would cause the osmotic imbalance and water efflux from ICS with concomitant cristae shrinking (narrowing), owing to parallel increased electroneutral phosphate efflux from ICS to the matrix. On proceeding OXPHOS after sufficient ATP accumulation, the mtK_ATP_ ensemble would close and water efflux from ICS would stop as would stop ICS shrinking (Fig. 18). This would also prevent an infinitive shrinking of cristae lamellae.

Thus, the high ATP demand and concomitant lowered Δp at high OXPHOS would require a highly regulated opening of mtK_ATP_ to mediate additional K^+^-efflux from ICS to the matrix even at otherwise inhibitory concentrations of ATP to maintain the ICS integrity. This could be achieved by an ICS-localized protein kinase, such as protein kinase Cɛ (Jabůrek et al., 2006).

Also, other ions could participate or regulate ion and water homeostasis. For example, a Ca^2+^/H^+^-antiport would have similar consequences as the K^+^/H^+^-antiport, with additional links to complex phenomena of Ca^2+^-transport and Ca^2+^-regulation, which are beyond the scope of this review. Thus, dormant OXPHOS, with inflated cristae would occur with faster (Ca^2+^)K^+^ influx into ICS. This can be presumably mediated by LETM1, a putative Ca^2+^/H^+^-antiporter, which was reported to be a K^+^/H^+^-antiporter in humans (Natarajan et al., 2021), probably at the inhibited efflux from ICS (and matrix uptake) *via* the Ca^2+^-uniporter or mtK_ATP_, respectively. Following the conditions of lower demand for ATP and a fractional Δp increase due to lower OXPHOS intensity, the reversal of phosphorylation-mediated activation of mtK_ATP_ could be achieved by a putative protein phosphatase inhibiting its activity.

#### 4. Rearrangement of cristae morphology and cristae dynamics

During very short time intervals, only mechanistic plus ionic/osmotic forces may play a role in cristae dynamics. During intermediate intervals, in which post-translational modifications would sufficiently contribute, these may participate as well. In the long term, all components of mitochondrial biogenesis including the action of assembly factors for ATP-synthase and RC complexes, which inherently concerns also with mtDNA expression and mt-ribosomal translation of mtDNA-encoded subunits, all these factors are involved.

Also, the required recruitment of phospholipids and CL for *de novo*-created CM should be considered (Schlattner et al., 2014; Tatsuta and Langer, 2017a). All these aspects to be described would compose another review, so next, we discuss only the short-term events. However, please note that cristae distortions in pathologies originate predominantly from the long-term causes.

Hypothetically, one can predict that (1) *only mechanistic forces*, or (2) *only ionic/osmotic forces* or (3) *both*, mechanistic plus ionic/osmotic forces, participate in short-term cristae dynamics. By mechanistic forces, we mean that CJs are mobile, subjected to a short-range 2D-diffusion or wobbling within IBM/OMM, so to be able to enlarge or decrease the area of the crista outlet. If individual MICOS-SAM complexes are detached, MICOS contributes to the opening of crista outlets.

If there are more such MICOS complexes (even if staying attached to mobile SAM), their movement would enable also longitudinal movement (oscillations) within the IBM cylindrical tubuli. For example, the circular projection of the crista outlet to the IBM surrounded by MICOS could hypothetically change from a slit-like to the more bulky/ellipsoid projection by MICOS recruitment (Plecitá-Hlavatá et al., 2016).

When only mechanistic forces are involved, the elasticity of OPA1 (or its hypothetical filaments) and PHB rings in the ICS lumen might ensure that the ICS and the entire crista lamella is inflated, pushing two parallel CMs of lamella apart from each other. If no ionic fluxes across CM are involved, water must penetrate the ICS lumen simply *via* the more opened crista outlet. In the 2D section perpendicular to IBM, one can imagine this as the sliding of two MICOS complexes apart (Fig. 14A, B), while taking adjacent CM apart as well (Dlasková et al., 2019; Plecitá-Hlavatá et al., 2016).

In this type (1) of cristae lamellae inflation, the necessary widening of the crista edges with ATP-synthase rows could either proceed as a passive mechanistic response on a push of CM apart each other, including the pressure by water uptake into ICS *via* the crista outlet. Additional mechanisms such as detachment of “glue” proteins between ATP-synthase dimers could also occur, hypothetically due to post-translational modifications. Note, that in case (1) Brown motion of MICOS can be the original impulse for cristae dynamics, besides numerous combinations of assembly/recruitment *versus* detachment of various MICOS subunits.

When we speculate on case (2), so that only pure ionic/osmotic forces initiate or drive cristae dynamics, and when MICOS movement is only secondary and passive, we must expect the existence of an original impulse for activation of putative mt-ion-channels. As stated above, ICS inflation would be automatically possible in conditions, when OXPHOS is dormant or when ATP-synthase runs backward in the ATP-ase mode (pumping protons), or on RET, when the Complex I runs backward and depolarizes Δ*p* and Δ*Ψ*_m_. In this case, the CM_m_ matrix surface is positively charged (unlike when the regular proton pumping to the ICS creates the negatively charged CM_m_,), which allows the K^+^ efflux from the matrix to the ICS lumen *via* mt-K^+^-channels, with concomitant anion efflux of phosphate or other anions, mediated by certain SLC25 family carriers or *via* inner membrane anion channel (IMAC) (Borecky et al., 1997). Consequently, the osmolarity imbalance would cause the water uptake into ICS and ICS inflation.

It is, however, plausible that both mechanistic and osmotic forces participate in cristae dynamics, having even two different impulses for initiation. Further studies are required to determine precise localizations of mt-ion-channels, either in CMs or within IBM, as well as studies of dynamics of assembly/recruitment of MICOS, OPA1 filaments, and ATP-synthase arrays.

#### 5. Cristae morphology on uncoupling

Cristae are reshaped also by mild or total uncoupling. For the latter case, when mt-network fragmentation is complete, cristae are rearranged in resulting ∼μm toroids of a distinct morphology, since parts of the OMM then form interior spheroids within a toroid (Ding et al., 2012) This greatly differs not only from the intact but also from the apoptotic morphology. Often the fragments engulfed also a portion of the cytosol. At a lower resolution, this engulfment is reflected by apparent matrix space toroids (Fig. 1A, rightmost panel) (Plecitá-Hlavatá et al., 2008).

Even a mild uncoupling could exert a profound influence on cristae morphology. Hence, mild uncoupling regulated mainly by mt UCPs (Jezek et al., 2018) provides an independent regulatory entity for cristae and mt-network morphology, sacrificing a tiny fraction of less efficient ATP synthesis. Also, on cristae rearrangement, CoQ diffusion could be altered only between Complex II or other oxidoreductases and Complex III. Thus, a higher superoxide formation under “flat cristae” conditions would be also given by longer distances for CoQ diffusion.

#### 6. Other proteins responsible for cristae rearrangement

The rearrangement of cristae morphology in relation to mtDNA, its organization, and its expression machinery represents a topic, which would again cover another review article and hence is beyond the scope of this one. Here, we mention only several aspects concerning relation to CM. The ATPase family AAA domain containing 3A (ATAD3A) protein is regularly distributed along CM or IBM acting in the mtDNA-nucleoid organization, cholesterol metabolism, and translation on mt-ribosomes (Peralta et al., 2018). ATAD3A ablation specifically in neurons led to a severe encephalopathy with aberrant cristae due to a loss of the regular ATAD3A interactions with Complex I, LETM1, and PHB (Arguello et al., 2021).

Also, p66Shc proteins could belong to the other cristae-shaping players. Indeed, p66Shc is phosphorylated by PKC when initiation by cell redox signaling or oxidative stress takes place (Mehta and Mehta, 2014). Phosphorylated p66Shc migrates into the ICS. This likely affects also cristae morphology since it induces mt-network fragmentation (Giorgio et al., 2005; Ulivieri, 2010). Indeed, DRP1-induced mt-network fission together with cristae remodeling could redistribute cytochrome ***c*** from the ICS to IMS (and to the cell cytosol on OMM permeabilization).

This fission results in a higher p66Shc content and increased superoxide/H_2_O_2_ production, but is prevented by p66Shc shRNA silencing, resulting in a decline in matrix-released H_2_O_2_ (Galimov et al., 2014). Also, overexpression of p66Shc in Jurkat T cells partially depleted cristae on pro-apoptotic stimuli, while the remaining ICS volume was inflated (Pellegrini et al., 2007). Note also that the cristae remodeling by p66Shc is independent of the permeability transition pore.

Finally, LETM1 was suggested to be also a cristae-shaping protein, residing at IBM. *In vitro* insertion of LETM1 into membrane bilayers formed membrane invaginations, thus speculations were made that besides its transport function, LETM1 may participate in cristae architecture, but when residing within IBM (Nakamura et al., 2020; Natarajan et al., 2021; Shin et al., 2013).

### B. Cristae dependence on metabolic changes

#### 1. Cristae widening and consequences for bioenergetics and superoxide formation

In general, intact and optimum cristae morphology favors the stability of RC supercomplexes and promotes OXPHOS, enabling mitochondrial-dependent cell growth (Cogliati et al., 2013). Let us theoretically recapitulate that crista widening may occur (1) *after water intake into ICS* promoted by the simultaneous efflux of K^+^ and anions, such as phosphate, from the matrix; (*e.g*., Kravenska et al., 2021). (2) *after decomposing ordered raws of ATP synthase dimers* on crista edges (rims) and relocations of MICOS/OPA1-filaments/PHB (Plecitá-Hlavatá et al., 2016); or (3) *by fusion of two adjacent crista lamellae* (Kondadi et al., 2020).

The event (1) exists typically when Δ*Ψ*_m_ is low or at least local Δ*Ψ*_m_ becomes positive in a single crista, that is, positive at the matrix CM_m_ surface and negative inside the ICS lumen at the CM_ICS_ surface. The latter could result from RET when electrons pass through the Q-tunnel of Complex I up to I_F_ site and Complex I proton pumping reverses so as to pump protons from the ICS to the matrix. Case (2), representing the initial event of decomposition of ordered raws of ATP-synthase dimers, can be hypothetically induced by post-translational modifications of “glue” proteins such as Mic10, which otherwise strengthen these raws. It should also happen when insufficient mtDNA expression and mt ribosome translation do not keep pace with nuclear-encoded and cytosolic OMM-associated ribosomes, that is, when ATP-synthase biogenesis is crippled.

Such a situation frequently occurs in cancer cells and is considered to result from the so-called *mitochondrial unfolded protein response* (UPR) (Smyrnias, 2021; Wang et al., 2022; Zhu et al., 2021). Case (3) needs to be experimentally proven if exists. Neither proteins that would participate in cristae fusion are known.

The excessive cristae widening could frequently implicate severe consequences for bioenergetics *per se* and be a diagnostic for these severe events such as repolarization of cristae membrane or insufficient biogenesis of ATP-synthase. If case (3) exists, one may envisage complete bioenergetics in the resulting bulky crista, however with longer diffusion distances for H^+^ inside the ICS lumen (Rieger et al., 2021).

CoQ diffusion and cytochrome ***c*** sliding within the supercomplexes are not affected in bulky cristae, unless supercomplexes are also disordered or cytochrome ***c*** is released, such as on apoptosis. A general rule could be that a relative delay in H^+^ diffusion (coupling) may relatively delay the electron transport and hence adequately partially increase superoxide formation (Fig. 17). However, due to many superoxide forming sites, each should have been inspected separately to predict consequences.

In the opposite case of thin (thinnest) crista lamellae and maximum ordered morphology, substrate overload governs the intensity or rate of superoxide formation. Assuming an optimum metabolic rate, any overload of either NADH or QH_2_ (such as from succinate, FAs, BCKAs, or specific substrates of Q-linked enzymes) could lead to the excessive superoxide formation. Such formation should not be regarded as oxidative stress, since it simultaneously represents a redox signal.

**FIG. 19.**
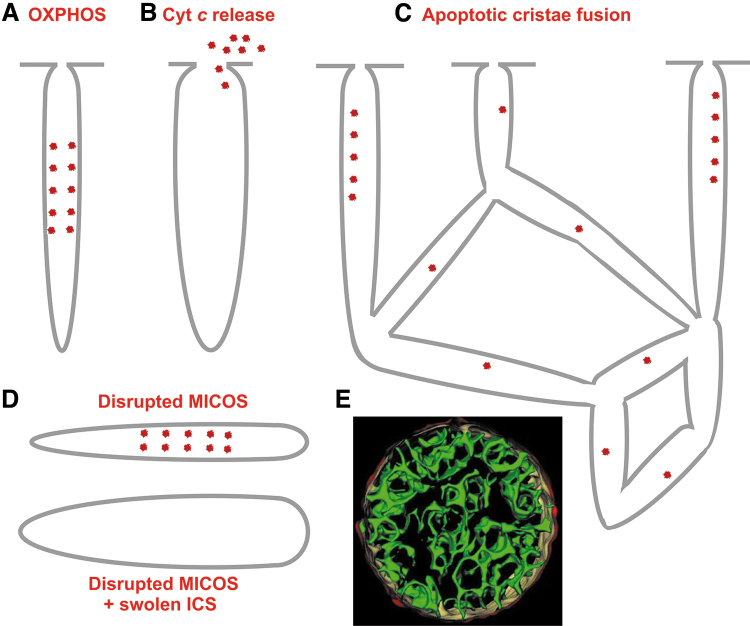
**Cristae opening and fusion on apoptosis. (A)** Cytochrome ***c*** (*red*) in intact “OXPHOS” cells; **(B)** cytochrome ***c*** release at the initiation of apoptosis; **(C)** apoptotic fusion of cristae—scheme and micrograph in **(E)** from Mannella (2008). **(D)** Impaired MICOS unable to hold crista junctions lead to cristae vesicles imide the matrix that had no connections to IBM and intermembrane space. After being swollen, they might also lose cytochrome ***c***.

Besides the cases of apoptotic cristae (Figs. 17 and 19) and partial/complete lack of cristae at pathologies, one can predict four situations as illustrated in Figure 19. These are combinations of wide/bulky cristae with low and high superoxide formation and narrow and ordered cristae with low and high superoxide formation. The wide-low situation occurs at OXPHOS dormancy, such as after hypoxic adaptation or respiratory substrate shortage, but the wide-high situation is common and often related to pathological states. In turn, the narrow-low(er) situation is the most physiological one and can be considered normal, whereas the narrow-high situation occurs at relatively high substrate pressure, that is, excess of respiration substrate.

#### 2. Specific features of substrate overload

Experimentally, forcing cells to respire on galactose could be regarded as a relative substrate overload. Indeed, switching cells from glucose to galactose-containing media led to cristae narrowing (Quintana-Cabrera et al., 2021), similar to the sudden substrate addition to hypoxia-adapted cells (Dlasková et al., 2019; Plecitá-Hlavatá et al., 2016). Unlike with glucose, in galactose-containing medium mouse adult fibroblasts (MAF^Gal^) overexpressing OPA1 however produced less superoxide, monitored with redox probe mt-roGFP1, relatively to wt cells (Quintana-Cabrera et al., 2021).

So, the cristae stabilizing role of OPA1 contributed to the decreased superoxide formation. A similar difference was observed with antimycin-induced superoxide. Surprisingly, silencing of F_O_-ATP-synthase subunit ***e*** prevented the OPA1 effect, indicating cristae stabilizing role now for subunit ***e***, speculatively stabilizing edges of crista lamellae. These data also show that, in the hierarchy, the subunit ***e*** is more important. The reported mild OPA1 overexpression even prevented cell death induced with antimycin.

Having less ATP-synthase and OPA1 oligomers in BN-PAGE, MAF^Gal^ exhibited lower in-gel ATP-hydrolysis, which increased with OPA1 expression (as well as ATP-synthase oligomers), but it did not reach the intensity found in glucose. Vice versa, OPA1 deletion diminished ATP-synthase oligomers. All these results support OPA1 and rows of dimeric ATP-synthase at crista rims as stabilizing and structurally required factors.

In contrast, under high ADP concentrations, cristae were observed as widened (Colina-Tenorio et al., 2020; Stephan et al., 2020). Since high ADP at low ATP and dormant OXPHOS determines low ATP synthesis, the cristae widening would be expected (Fig. 17). Overall increase of mitochondrial biogenesis logically promotes OXPHOS, also by the increasing number or density of cristae. Thus, regular exercise leads to an elevated density of mitochondrial cristae in trained athletes (Nielsen et al., 2017). In contrast, cristae widening (ICS inflation) was observed in differentiating cells (Wasilewski et al., 2012).

Imperfect biogenesis then leads to imperfect or disrupted cristae (see below). Thus, disruption of cristae on severe oxidative stress in mice with cardiomyocyte-specific SOD2 ablation has been described (Sharma et al., 2020). When such oxidative stress is prevented, for example by vitamin D supplementation, the shape of cristae is normalized due to the correction of MFN1/2, OPA1, and DRP1 expression (Ren et al., 2020).

Type of nutrition matters in a long term. Thus, for example, long-term β-hydroxybutyrate supplementation decreased MFN2 levels significantly in murine skeletal muscle mitochondria, reducing cristae (Monsalves-Alvarez et al., 2020). White adipocyte browning was linked to the urea cycle, whereas the cristae morphology maintenance by OPA1 was emphasized (Bean et al., 2021). OPA1 facilitated adipocyte browning, that is, transformation to BAT type mitochondrion with rich narrow cristae filled by cytochrome ***c*** (an origin of brown appearance).

Mature brown adipocytes then dissipate energy by UCP1-mediated thermogenesis, so counteracting obesity. On the contrary, in patients with reduced OPA1, obesity was manifested. Fumarate was one of the factors initiating OPA1-dependent browning (meaning browning dependent on a correct normal cristae shape). Adipocyte-specific OPA1 deletion prevented such browning. The correct OPA1 function was found to be essential also for thymocyte maturation, since OPA1 deletion resulted in the lack of metabolically fit long-term memory T cells (Corrado et al., 2021).

#### 3. Cristae in hypoxic cells

One can consider hypoxia as a state approaching ischemia and hence consider an analogy with heart ischemia. However, supraphysiological hypoxia representing rather a hyperoxia exists in experiments, since with exceptions of lung and few other cell types, cells withstand much lower oxygen tension physiologically. Nevertheless, mechanisms were developed for adaptation to the range of low O_2_, below physiological hypoxia. Only below a threshold, under which the pathological hypoxia exists, these mechanisms can no longer help cells to adapt and cell death mechanisms follow. Note that K_m_ for oxygen of cytochrome ***c*** oxidase (CIV) is around 200 μ*M*.

Within numerous pieces of literature on hypoxia, it is difficult to distinguish pathological changes of cristae morphology from “physiological” ones. We attempted for the latter, observing the cristae widening after hypoxia adaptation of HepG2 cells (Plecitá-Hlavatá et al., 2016). The mechanism involved the MIC60 degradation with concomitant increases in interdistances between MICOS complexes and hence widening or opening of crista outlets, leading mechanistically (or osmotically) toward ICS inflation (Fig. 14A, B).

One can regard the observed changes as physiological due to their reverse character. Indeed, a sudden addition of respiration substrate to hypoxia-adapted HepG2 cells instantly (within a few minutes) led to cristae narrowing (Dlasková et al., 2019). Speculatively, the recruitment of MIC60 or other MICOS subunits back, together with all benefits of high Δp (cf. Section V.A.3 above) should contribute to such cristae narrowing.

## VI. Mitochondrial Morphology, Oxidative Stress, and Diseases

### A. Cristae on apoptosis and pathologies

#### 1. Apoptotic rearrangement of cristae morphology and impact on RC supercomplexes

Fission of mt network is frequently accompanied by cristae remodeling, such as dilatation, vesiculation, or even a complete CM disappearance (Knott et al., 2008; Zick et al., 2009). When the mitochondrial route of apoptosis is induced, cristae are remodeled (Scorrano et al., 2002) (Fig. 19). The crista outlets are widened or broken since cleavage of OPA1 complexes (heterotrimers or, speculatively, lattices) accompanies such induction (Cipolat et al., 2006; Frezza et al., 2006); together with a concomitant detachment of MICOS from SAM, hence detachment from OMM.

Consequently, disruption or wide opening of cristae outlets enables 2D diffusion of cytochrome ***c*** first to the IBM surface within the IMS, incoming from the internal ICS surface of cristal membranes. Thereafter, the canonical apoptotic initiation is given by the cytochrome ***c*** leakage to the cell cytosol, when concomitant changes take place in OMM, being orchestrated by the interplay between anti-apoptotic proteins and migration to IMS of pro-apoptotic BH3-only BCL-2 family members such as BID, BIM-S, or BNIP3.

This results in the creation of BAX-BAK oligomeric channels or holes for cytochrome ***c*** (Cipolat et al., 2006; Frezza et al., 2006), which colocalize with DRP1 pro-fission (Otera et al., 2016) and MFN2 pro-fusion mitochondrial proteins (Karbowski et al., 2004) and promote also apoptotic mt-network fragmentation (Wasiak et al., 2007). This simultaneously allows Ca^2+^ signals from ER to mitochondrion, which further amplify the cytochrome ***c*** release.

Concomitant changes in MICOS-SAM interactions and OPA1 structure are parallel to the recruitment of BH3-only proteins (Yamaguchi et al., 2008), which enlarge crista outlets and widen the area of CJs (Frezza et al., 2006). All these changes allow a new rearrangement of the cristae shape. Since this proceeds also in cells lacking BAX and BAK (Yamaguchi et al., 2008), cristae remodeling cannot occur downstream of caspase activation.

The exact mechanism leading to apoptotic cristae morphology changes is unknown. Interaction of pro-apoptotic BCL-2 family members with OPA1 was suggested to initiate them (Landes et al., 2010). Alternatively, ER-OMM stabilization-dependent Ca^2+^ overload of the matrix was suggested to be the initiator (Germain et al., 2005) with involved mitochondrial permeability transition (Bernardi et al., 2021; Giorgio et al., 2019) or chaperone TRAP1 (Cannino et al., 2022).

Even an inversion of the cristae curvature may exist as inferred from the observed fused cristae reticulum (Frey et al., 2002; Mannella, 2020). It is plausible that on apoptotic cristae reshaping, supercomplex structure and/or stability can be affected, which together with the lack of cytochrome ***c***, decreases in respiration and increases in superoxide formation at least at site III_Qo_. On initiation of apoptosis, IMM/CM and hence cristae are remodeled into many separate vesicular matrix compartments (Fig. 19) (Sun et al., 2007). This is accompanied by the apoptotic release of proteins. However, such remodeling is not essential for cytochrome ***c*** release. Loss of Δ*Ψ*_m_ occurred only late in apoptosis after the release of cytochrome ***c***.

The cytochrome ***c*** escape from the CMs also leads to further elevation of Complex III superoxide/hydroperoxyl radical formation, thus escalating a pro-oxidative vicious cycle. This can be regarded as pro-apoptotic redox signaling, which is correlated with the second “break” of the ICS, represented by an opening of permeability transition pores. In some of these cases, extreme cristae remodelation occurs, where the resulting fused cristae form the *cristae reticulum*. On etoposide-induced apoptosis, in *stage 1* neither cytochrome ***c*** is released, nor Δ*Ψ*_m_ is lost, and TEM-visualized cristae still maintain “textbook” morphology.

In *stage 2*, cells are releasing cytochrome ***c*** but still Δ*Ψ*_m_ is maintained and cells exhibit also many TEM-visualized mitochondria with vesicular matrix compartments. Finally, in *stage 3*, Δ*Ψ*_m_ is lost and TEM images show swollen cristae morphology and vesicular-swollen mitochondrial sections (Sun et al., 2007). Cryo-EM tomography displayed cristae lamellae in Stage 1 and inflated or cylindrical lamellae in stages 2 and 3 (Sun et al., 2007). Authors suggested that the observed apoptotic cristae morphology resulted from the extreme widening of crista outlets (CJs).

Cristae membrane could be enfolded on ICS swelling so as to attack OMM by herniation (Mannella, 2020). This was observed in late apoptosis in FAS-activated hepatocytes, when the content of herniated crista is expelled into the cytosol, including cytochrome ***c*** (Mootha et al., 2001). A permeability transition pore phenomenon was implicated in such herniation (Feldmann et al., 2000). In apoptotic mouse embryonic fibroblasts, this was preceded by cytochrome ***c*** protruding through oligomeric BAK and BAX megapores in OMM, while local megapore accumulation caused OMM ruptures (McArthur et al., 2018).

#### 2. Cristae in ferroptosis

Ferroptosis is another type of regulated cell death, distinct from apoptosis or necroptosis. Its distinct morphological characteristics include smaller mt-network sections in TEM with condensed IMM densities and OMM ruptures (Wang et al., 2020). Cristae appear more electron-dense and mt-network sections are shrunken (Zhang et al., 2020b). Ferroptosis induction involves inhibition of cystine/glutamate antiporter (its SLC7A11 subunit) in the plasma membrane, activation of MAPK pathways and OMM VDAC, and ER stress.

Since ferroptosis depends on accumulations of products of lipid peroxidation and lethal ROS production due to dysregulated iron metabolism, it can be counteracted by glutathione peroxidase 4 (GPX4), heat shock protein β-1, and nuclear factor erythroid 2-related factor 2 (NRF2). GPX4 thus attenuates lipid peroxidation whereas NRF2 decreases cellular iron uptake. Similar to the hypoxic activation of PHDs, Complex III was suggested to be the primary superoxide source on ferroptosis induced by cysteine starvation (Homma et al., 2021).

Misregulated ferroptosis besides attenuated apoptosis exists in numerous cancer cells. In contrast, one may speculate that ongoing ferroptosis alters normal cristae morphology due to disruption of normal ER-OMM contacts, by the interference of lipid peroxidation products with CM lipids and by secondary self-perpetuating effects of the increased mitochondrial superoxide formation.

### B. Cristae in diseases

Numerous pathologies are associated with irregular cristae reflecting states shifted from normal mitochondrial maintenance. These ultramorphology changes typically have multiple origins, but in numerous cases, an imbalance in cristae shaping proteins is the main cause as will be illustrated below. Accepting that a frequent hallmark of pathology is the disrupted cristae architecture; consequently, dysfunction of all related biology must be expected.

The chicken-and-egg problem in what was the initial event does not much affect the progressive consequences. Initiation may originate from mtDNA mutations to dysfuncional mtDNA-maintenance machinery, both causing inadequate Complex I (supercomplex), Complex III, and ATP-synthase function and architecture, which results immediately in disrupted cristae. Initiation could origin from altered metabolism and related changes in the redox state, a typical example being RET.

Oxidative stress conditions frequently disrupt cell homeostasis, so that it leads also to abnormal cristae. In the following sections, we exemplify how cristae morphology reflects and/or contributes to pathologies. We do not have the ambitions to write a complex review on the topic. Excellent reviews can be found in the literature (*e.g*., Fernandez-Vizarra and Zeviani, 2021; Garone et al., 2022; Gorman et al., 2016; Navaratnarajah et al., 2021; Pernas and Scorrano, 2016; Vincent et al., 2016; Zeviani and Carelli, 2021).

It could be emphasized that there is almost no disease that would not be somehow reflected by alternations of mitochondria, including cristae. Thus, for example, glomerular mitochondria from high-salt-fed Dahl salt-sensitive rats possess swollen and less defined cristae in TEM sections (Domondon et al., 2019). Disrupted cristae can be also found in cardiomyocytes of a rat model of sepsis having also complex IV deficiency (Yang et al., 2019). Other examples can be found in mitochondrial myopathies (Vincent et al., 2016).

Also, aging was found to be related to altered cristae morphology. Thus, ATP-synthase dimerization was found to be disrupted with concominant alternations in cristae morphology in aged mice (Bou-Teen et al., 2022; Daum et al., 2013) and *Podospora anserina* (Warnsmann et al., 2021). Numerous findings in lower organisms supported the idea of adjusted cristae morphology in aging. However, not always “young” morphology was causal for higher life-span (Warnsmann et al., 2022).

#### 1. Cristae in cancer

The current view of carcinogenesis and cancer metabolism extends from considerations of mixed Warburg and OXPHOS phenotype, *via* considerations of the requirement of mitochondria for the synthesis of nucleotides, to the knowledge of all details of different types of cancer and recognition of different metabolic cell types within a single solid tumor, up to the important role of a metabolic and information crosstallk between cancer-associated cells (immune cells, fibroblasts) and tumor cells.

Having these pleiotrophic situations, there is no rule on the requirement of mitochondria in their typical OXPHOS role, as well as no rule can be derived for cristae morphology in cancer cells, nor for prediction of superoxide formation. Simply there are no cancer cristae.

In numerous cases, cancer cell mitochondria appear to be small in TEM sections and lack cristae, which implicates the lack of properly organized OXPHOS machinery (Arismendi-Morillo, 2009; Arismendi-Morillo and Castellano-Ramirez, 2008). For example, a deficiency in the ATP-synthase β-subunit was reported (López-Ríos et al., 2007). Often mitochondrial biogenesis is decreased, given by the decreased PGC1 expression, such as in lung (Bellance et al., 2009) and breast cancer (Watkins et al., 2004), consequently decreasing also mitochondrial transcription factor and mtDNA nucleoid structural protein TFAM.

Decreased amounts of mtDNA have also been reported. Decreased levels of RC complexes have been associated with renal cell carcinoma (Simonnet et al., 2003). Mutations in mtDNA and consequent oxidative stress have been shown to regulate cell growth and metastatic potential (Ishikawa et al., 2008).

On the other hand, there are cancer cell types exhibiting increased mitochondrial biogenesis associated with cristae narrowing and enrichment, such as ovarian cancer (Signorile et al., 2019). When the ordered narrow cristae structure is disrupted in cancer cells relying on OXPHOS, the growth is retarded (Herkenne and Scorrano, 2020; Zamberlan et al., 2022). Likewise, angiogenesis depends on correct OXPHOS and hence on intact cristae morphology (Herkenne et al., 2020).

#### 2. Cristae in diabetes

Type 2 diabetes is currently recognized to stem from the initial pathology of pancreatic β cells and their dedifferentiation, whereas autoimmune disruption of β cells causes type 1 diabetes. Hence, pancreatic β cell ultramorphology could be altered even in prediabetic states, whereas mitochondria in peripheral tissues can be affected by inflammation of white adipose tissue and progressively developed insulin resistance (the inability of insulin receptor pathway to respond). Thus, for example, mitochondria of β cells from 10-week-old MKR diabetic mice were reduced in the apparent number and had severely swollen disordered cristae (Lu et al., 2010).

In progressed type 2 diabetes states, β cells can undergo apoptosis or other specific types of cell death such as ferroptosis with concomitant characteristic changes in cristae morphology (Li et al., 2020a).

Concerning peripheral tissues in diabetes, a profound contribution of the dysregulated mitochondrial morphology, dynamics, and function was implicated. Simulating type 1 diabetes by streptozotocin-induced diabetes in rats, it was observed that liver mitochondria had a reduced number of cristae (Welt et al., 2004). Mitochondrial cristae density was also diminished in the cardiac mitochondria of type 1 diabetic Akita mice (Bugger et al., 2009).

In the traditional view of Hackenbrock terminology, it was reported that in diabetic tissues a condensed state of cristae structure *in situ* (*i.e*., cristae expansion, matrix condensation) is found in conjunction with fragmented mt network and low respiration (Pagano et al., 2014). The role of OPA1 in cristae architecture is also reflected by experiments demonstrating an impaired GSIS, due to the decreased complex IV content and activity when OPA1 was deleted (Zhang et al., 2011). Also, correct DRP1 regulation in conjunction with Ca^2+^-homeostasis in skeletal muscle can be disrupted (Favaro et al., 2019).

Diabetic Goto Kakizaki rats, when fed by high-fat diet, exhibited ventricular myocytes with extensive mitochondrial lesions, namely loss of cristae, and reduction in mitochondrial density (Howarth et al., 2011). Gestational diabetes mellitus is associated with a heightened level of mitochondrial oxidative stress to which relations between DRP1-mediated fission and p66Shc can contribute (Huang et al., 2021). In the liver, this is mediated *via* the endothelin type A receptor (Feng et al., 2021).

In pancreatic β cells, a NOX-JNK-p66Shc signalosome pathway may also contribute (Elumalai et al., 2020). Analogously, the p66Shc protein links cytosolic and mitochondrial oxidative stress to the development of diabetic retinophathy (Mishra et al., 2019). A specific case of maturity-onset diabetes in the young (MODY), using its hyperglycemic mouse model GENA348, not developing hyperinsulinemia, was inspected in a dysfunctional left ventricular heart by serial block face SEM (Rajab et al., 2022). Subsarcolemmal mitochondria were twice larger but irregular due to MFN1, MFN2, OPA1, and PGC1-α upregulation on a stalled mitophagy due to PINK1, Parkin, and MIRO1 down-regulation, also with more irregular cristae.

Systemic inflammation in type 2 diabetes can be also reflected by cristae. Mesenchymal stem cells from healthy volunteers showed normal cristae morphology in portions of mt tubules, whereas TEM sections of samples from patients with type 2 diabetes visualized shorter and less frequent cristae in preserved mt tubules (Horiguchi et al., 2021). Obesity is associated with lowered mitochondrial function. Thus, diet-induced obese mice exhibited a pronounced decrease in OXPHOS machinery and cristae density in subcutaneous adipose tissue (Schöttl et al., 2020).

#### 3. Cristae in heart diseases

Aging is a factor in determining the onset of heart failure. Thus, heart mitochondria and cardiomyocytes from aging mice exhibited an impaired ATP-synthase dimerization, resulting in abnormal more flat cristae tip curvature (Bou-Teen et al., 2022). Palmitate-induced hypertrophy of neonatal rat cardiomyocytes was found to be attenuated by acetylcholine, which restores normal cristae morphology from fragmented and lysed cristae, presumably by increasing Mic60 and activating AMPK. The hypertrophy was associated with increased superoxide/H_2_O_2_ formation and just this was attenuated by acetylcholine (Xue et al., 2019).

Also, when cardiomyocytes were unable to upregulate mitophagic clearance on hypertrophic cardiomyopathy, a correlation was found with reduced cristae density, reduced citrate synthase activity, and coupled respiration, plus with increased superoxide/H_2_O_2_ and reduced antioxidant defense (Ranjbarvaziri et al., 2021). Similarly, Friedreich's ataxia causes fatal hypertrophic cardiomyopathy due to the deficiency of the mitochondrial protein, frataxin, required for iron homeostasis, for example, for iron-sulfur cluster and heme synthesis.

This was simulated in frataxin KO mice showing increased mt biogenesis in cardiomyocytes with more condensed cristae (Chiang et al., 2021). Induction of experimental auto-immune myocarditis promoted by lipopolysaccharide (LPS)-activation of toll-like receptors TLR4 also led to damaged cristae (Allen et al., 2020; Wu et al., 2018). TEM showed that IR caused an OMM rupture, cristae disappearance, and vacuolation, whiereas a flavonoid vitexin reduced mitochondrial damage and ultimately reduced cardiomyocyte apoptosis (Xue et al., 2020).

#### 4. Cristae in neurodegeneration

The aging of motor neurons leads to a lack of cristae at neuromuscular junctions (García et al., 2013). Clear implications are for OPA1, which was identified to be the main cause of autosomal dominant optic atrophy, that is, selective degeneration of retinal ganglion cells and optic nerve, accompanied by progressive and irreversible blindness (Alexander et al., 2000; Delettre et al., 2000). L-OPA1 protected ischemic brains, diminishing apoptosis of neurons and restoring normal cristae morphology, in contrast to excessive OPA1 cleavage after cerebral ischemia-reperfusion injury (Lai et al., 2020).

Lymphoblastoids cells of patients with autosomal dominant optic atrophy exhibited fragmented mt network and abnormal cristae morphology, lower amounts of mtDNA encoded RC complexes and ATP-synthase subunits, hence resulting lower respiration and Δψ_m_ and, consequently, the increase in accumulated ROS (Kao et al., 2015; Zhang et al., 2017). This could be simulated by OPA1 deletion in mouse embryonic fibroblasts. Also, dysregulation of aspartate metabolism was found (Bocca et al., 2018). The clinical severity is given mainly by impaired mtDNA and its consequences (Elachouri et al., 2011).

Scaffolding of PHB, otherwise stabilized by wt CHCHD10 in intact cells, is disrupted by S59L mutant of CHCHD10, inducing also aggregates of stomatin-like protein 2 with PHB complex in hippocampal neurons and spinal motoneurons (Genin et al., 2022). Aggregates also disrupt the OMA1 cascade and its OPA1 processing and incorrect OPA1/MICOS interaction, causing abnormal cristae and neuronal death. Thus, cristae pathogenesis is manifested as neuronal death in amyotrophic lateral sclerosis and frontotemporal dementia.

Impaired ER-mitochondrial connections were implicated in progressive hearing loss (Perkins et al., 2020). Pathogenesis of amyotrophic lateral sclerosis (ALS) was studied in the *Drosophilla* model (Li et al., 2020b). Patients with ALS had faint cristae and greater lysosomal bodies in platelet mitochondria (Shrivastava et al., 2011). Peripheral blood mononuclear cells of patients with multiple sclerosis exhibited deregulated OPA1 due to OMA1 inactivation and increasing PHB2, resulting in elevated superoxide/H_2_O_2_ production, speculatively providing resistance toward apoptosis (De Rasmo et al., 2020).

In a model of Huntington's disease, the R6/2 fragment mouse model, neurons of the cortex and striatum exhibited abnormal cristae due to impaired OPA1 oligomerization (Hering et al., 2017). It was assumed that Barth syndrome and mitochondrial encephalopathy are caused by mutations in TAZ and MIC13, respectively (Jimenez-Blasco et al., 2020; Madreiter-Sokolowski et al., 2020; Sies and Jones, 2020; Vicente-Gutierrez et al., 2019; Zaninello et al., 2022), where perturbations of cristae architecture manifest as cristae vesicles/sheets (Acehan et al., 2011; Acehan et al., 2007). Also, circular cristae were found in activated microglia *via* mt benzodiazepine receptor (Banati et al., 2004).

#### 5. Cristae in immune cells

The immune cells possess a smaller cytosolic compartment and hence a much smaller total volume of mitochondrion relative to the whole cell volume. This poses mitochondrial research in immune cells as rather difficult, despite mitochondrial redox signaling can participate in for example, NLRP3 inflammasome formation (Ježek et al., 2018; Jezek et al., 2010; Plecitá-Hlavatá et al., 2016; Plecitá-Hlavatá et al., 2015). Immune cells, such as macrophages, upon LPS stimulation, shift to dormant OXPHOS, while simultaneously increased succinate provides excessive superoxide/H_2_O_2_, which serves as redox signaling driving expression of pro-inflammatory genes (Mills et al., 2016).

Also, for signaling *via* toll-like receptors TLR3 and TLR4, which ensures cytokine production in macrophages, remodeling of RC is required for the required redox signals (Ahmed et al., 2019).

On maturation of eosinophils, cristae significantly increase in number and reshape to lamellar morphology. Cristae remodeling in eosinophils was induced in inflammatory conditions varying proportions of only lamellar *versus* tubular cristae (Bonjour et al., 2022). Thus, processes of eosinophilopoiesis and inflammation-induced activation are reflected or dependent on cristae remodeling.

Succinate provides signaling in immune and cancer cells (’Mills and O'Neill, 2014; Ryan et al., 2019; Tretter et al., 2016). An antimicrobial unsaturated dicarboxylic acid itaconate (methylene succinic acid) is formed by macrophages on LPS stimulation from aconitate by *cis*-aconitate decarboxylase. Since itaconate is a competitive inhibitor of SDH, it induces succinate accumulation. Parallel activation of NRF2 by itaconate in mouse and human macrophages upregulates the expression of antioxidant genes and interleukin-1β (IL-1β) production (Mills et al., 2018b). Itaconate alkylates Cys151, Cys257, Cys288, Cys273, and Cys297 on the protein KEAP1, which allows NRF2 activation.

#### 6. Cristae in mitopathies

Cultivated myoblasts from patients with mitochondrial disorders, such as chronic progressive external ophthalmoplegia (CPEO), mitochondrial encephalopathy lactic acidosis and stroke-like episodes (MELAS) and TMEM70 deficiency, had TEM section of mitochondria with fragmentation, shortening, and aberrant cristae (Sládková et al., 2015).

Fibroblasts of patients with Leigh Syndrome, that is, neonatal- or pediatric-onset sub-acute necrotizing encephalomyelopathy, a type caused by pathogenic mutation of DAPIT subunit of ATP-synthase possessed reduced cristae due to reduced ATP-synthase dimerization (Siegmund et al., 2018). Cristae appearance as cristal vesicle-sheets was observed for a patient with a mitochondriopathy (Frey and Mannella, 2000), and it was clearly observed when MICOS subunits were depleted (Rabl et al., 2009).

Also, Mic13 deficiency leads to the lethal infantile onset of mitochondrial hepato-encephalopathy characterized by neurological impairments, hyperlactatemia, 3-methylglutaconic aciduria, impaired hepatocytes cerebellar and vermis atrophy (Zeharia et al., 2016) or renal stones (Gödiker et al., 2018). The patient's fibroblast lacked Mic13 and Mic10 and hepatocyte mt spheroids contained cristal vesicle sheets due to loss of CJs and decreased OXPHOS (also in skeletal muscle), originating from impaired mtDNA expression (Kishita et al., 2020).

CJs in HeLa cells were also disrupted when Mic26 was lacking, causing also diminished respiratory capacity (Koob et al., 2015), which was explained by the instability of RC supercomplexes and diminished CL levels (Anand et al., 2020). Correspondingly, Mic26 mutations cause recessive mitochondrial myopathy (Benincá et al., 2021). Also, other cristae-shaping or influencing proteins were implicated in diseases.

Thus, mutant ATAD3A was linked to neurological syndromes (Harel et al., 2016), mutant TAZ in the Barth syndrome (Bione et al., 1996), while the Mic13 deficiency was c,orrelated to mitochondrial hepato-encephalopathy (Kishita et al., 2020). Enlarged mitochondria in sections with concentric cristae and dense mitochondrial matrix were observed in patients with mitochondrial myopathy, reversible infantile RC deficiency, associated with the homoplasmic m.14674T>C variant having a deficiency in NDUFB8 complex I subunit (Benincá et al., 2021; Roos et al., 2022).

## VII. Conclusive Remarks

Integrating yet unknown mechanisms of cristae ultramorphology changes into mitochondrial physiology and pathology and uncovering all the involved interrelationships are the aim of future studies. For cristae ultramorphology changes, the participation of ionic forces has to be solved, whether they participate or not. The ultimate goal is to recognize, when an altered cristae morphology and cristae lamellar structure is physiological and under which conditions this reflects an initiated or progressively ongoing pathology.

Also, more profound knowledge is required to characterize stages of major cell death types such as apoptosis and ferroptosis in relation to cristae. Not covered in this review, but uncovering relations of mtDNA nucleoids to cristae architecture and changes will contribute to our understanding of complex mitochondrion phenomena. Last but not least, it will be necessary to relate all of this to redox states, those physiological ones as well as to those already exceeding a threshold for oxidative stress and pathology.

Redox state should be judged according to new knowledge of structures of RC complexes and supercomplexes. For example, because of the resolved complete Complex I structure including the Q-tunnel and recent suggestions or models of the internal coupling of electron transfer to long-range conformational changes and H^+^ pumping, one must ask the question where exactly electron leaks from the semiquinone QH^•^ to oxygen? Is it at QBS_I_ or QBS_Im_?

Could it happen even inside the Q-tunnel? Is it at exactly the same locus at forward electron transfer and in conditions of RET? Also, NADH/NADPH homeostasis should be related to redox states. The recent annotation of SLC25A51 as the mitochondrial NADH importer (Girardi et al., 2020; Kory et al., 2020; Luongo et al., 2020; Ouyang et al., 2021; Ziegler et al., 2021) even further complicates analysis or prediction of NADH and NAD^+^ concentrations *in vivo*.

Future studies are required to estimate its range and maximum values of a possibly higher NADH/NAD^+^ in mitochondrion *in vivo* and to demonstrate whether superoxide is formed predominantly at site I_F_ when an optimum NADH/NAD^+^ ratio is exceeded. It should be resolved whether only cells with predominant OXPHOS would obey such a mechanism and whether cells with a dormant OXPHOS, such as cancer cells of certain cancer types, behave differently, due to their specific metabolism.

## Abbreviations Used

2D: two-dimensional
3D: three-dimensional
AA: amino acid
ALR: augmenter of liver regeneration
ANT: adenine nucleotide translocase
ATAD3A: ATPase family AAA domain containing 3A (protein)
BAT: brown adipose tissue
BCKADH, BCKDH: branched-chain 2-oxoacid dehydrogenase
CCDC51: coiled-coil domain-containing protein 51
CHDH: choline dehydrogenase
CI: complex I
CII: complex II
CIII: complex III
CIV: complex IV
CJ: crista junction
CL: cardiolipin
CM: crista membrane
CoQ: coenzyme Q
CYC1: cytochrome ***c***1 subunit
cyt ***c***: cytochrome ***c***
DH: dehydrogenase
DHODH: dihydroorotate dehydrogenase
ETF: electron-transferring flavoproteins
ETFQOR: electron-transferring flavoprotein ubiquinone oxidoreductase
FA: fatty acid
FIB/SEM: focused-ion beam/scanning electron microscopy
FIH: factor inhibiting HIF
FMN: flavin mononucleotide
GAPDH: glycerol-3-phosphate dehydrogenase
GPX4: glutathione peroxidase 4
GSIS: glucose-stimulated insulin secretion
H^−^: hydride
H_2_O_2_: hydrogen peroxide
HIF: hypoxia-inducible factor
HisH^+^: imidazolium
IBM: inner boundary membrane
ICS: intracristal space
IMAC: inner membrane anion channel
IMM: inner mitochondrial membrane
IMS: intermembrane space
IMS_p_: peripheral intermembrane space
IR: ischemia/reperfusion
ISP: iron-sulfur protein
KO: knockout
LETM1: leucine zipper and EF-hand containing transmembrane protein 1
L-OPA: long OPA
LPS: lipopolysaccharide
MAF^Gal^: galactose-containing medium mouse adult fibroblasts
MGM1: mitochondrial genome maintenance 1
MIA40: mitochondrial intermembrane space import and assembly protein 40
MICOS: mitochondrial cristae organizing system (complex)
MICU1: mitochondrial calcium uptake 1
mt: mitochondrial
mtDNA: mitochondrial DNA
mtK_ATP_: mitochondrial ATP-sensitive K^+^-channel
NNT: nicotinamide nucleotide translocase
NOX: NADPH oxidase
NRF2: nuclear factor erythroid 2-related factor 2
O_2_^•−^: superoxide
OGDH: 2-oxoglutarate dehydrogenase
OMM: outer mitochondrial membrane
OPA1: optic atrophy 1
OXPHOS: oxidative phosphorylation
PC: phosphatidylcholine
PDH: pyruvate dehydrogenase
PE: phosphatidylethanolamine
PHB: prohibitin
PHD: prolyl hydroxylase domain, that is, 2OG-dependent-prolyl hydroxylases (EGLN)
PINK1: PTEN-induced putative kinase-1
Q: ubiquinone
QBS: Q/QH_2_ (ubiquinone/ubiquinol) binding site
QH_2_: ubiquinol
RC: respiratory chain
RET: reverse electron transfer
ROS: reactive oxygen species
S1QEL: suppressor of site I_Q_ electron leak
S3QEL: suppressor of site III_Q_ electron leak
SAM: sorting and assembly machinery (complex)
SDH: succinate dehydrogenase (CII)
S-OPA: short OPA
TEM: transmission electron microscopy
TIM: translocase of the inner membrane
TOM: translocase of the outer membrane
UCP: uncoupling protein
VDAC: voltage-dependent anion channel
